# Donghaecyclinones A–C: New Cytotoxic Rearranged Angucyclinones from a Volcanic Island-Derived Marine *Streptomyces* sp.

**DOI:** 10.3390/md18020121

**Published:** 2020-02-18

**Authors:** Munhyung Bae, Joon Soo An, Seong-Heon Hong, Eun Seo Bae, Beomkoo Chung, Yun Kwon, Suckchang Hong, Ki-Bong Oh, Jongheon Shin, Sang Kook Lee, Dong-Chan Oh

**Affiliations:** 1 Natural Products Research Institute, College of Pharmacy, Seoul National University, Seoul 08826, Korea; baemoon89@snu.ac.kr (M.B.); ahnjunsoo@snu.ac.kr (J.S.A.); sung954@snu.ac.kr (S.-H.H.); ddol1289@snu.ac.kr (E.S.B.); kisi2016@snu.ac.kr (Y.K.); shinj@snu.ac.kr (J.S.); sklee61@snu.ac.kr (S.K.L.); 2 Department of Agricultural Biotechnology, College of Agriculture & Life Sciences, Seoul National University, Seoul 08826, Korea; beomkoo01@snu.ac.kr (B.C.); ohkibong@snu.ac.kr (K.-B.O.); 3 Research Institute of Pharmaceutical Sciences, College of Pharmacy, Seoul National University, Seoul 08826, Korea; schong17@snu.ac.kr

**Keywords:** molecular modeling, electronic circular dichroism, quantum mechanics-based computation, angucyclinone, *Streptomyces*, cytotoxicity

## Abstract

Chemical profiling of the *Streptomyces* sp. strain SUD119, which was isolated from a marine sediment sample collected from a volcanic island in Korea, led to the discovery of three new metabolites: donghaecyclinones A–C (**1**–**3**). The structures of **1**–**3** were found to be rearranged, multicyclic, angucyclinone-class compounds according to nuclear magnetic resonance (NMR) and mass spectrometry (MS) analyses. The configurations of their stereogenic centers were successfully assigned using a combination of quantum mechanics–based computational methods for calculating the NMR shielding tensor (DP4 and CP3) as well as electronic circular dichroism (ECD) along with a modified version of Mosher’s method. Donghaecyclinones A–C (**1**–**3**) displayed cytotoxicity against diverse human cancer cell lines (IC_50_: 6.7–9.6 μM for **3**).

## 1. Introduction

Quantum mechanics–based computation is emerging as a useful tool for elucidating the structure of natural products and complements the analysis of experimental spectroscopic data [1,2,3]. Modern computational techniques, when coupled with a nuclear magnetic resonance (NMR) shielding tensor, allow us to clarify the assignment of individual nuclei when considering experimental NMR data and establish relative configurations [3,4]. In particular, the recent development of advanced statistical analyses (for example, CP3 and DP4) has enabled us to assign the relative configurations of natural products possessing remote stereogenic centers that are located multiple bonds away from the other chiral centers with assigned configurations. These analyses utilize computed ^1^H and ^13^C NMR chemical shift values based solely on computational methods and statistically compare them with experimental NMR chemical shifts using web-based applets provided by the Smith and Goodman groups [3,5,6]. Despite the usefulness of the computational methods that utilize an NMR shielding tensor for the assignment of relative configurations, these techniques are not applicable to absolute configurations because NMR spectroscopy cannot inherently distinguish enantiomers. However, over the past few decades, electronic circular dichroism (ECD) spectra have been used to determine the absolute configurations of natural products [7,8]. Advanced ECD calculations, when coupled with time-dependent density functional theory (TD-DFT), have resulted in higher accuracy and lower computational costs and enable the assignment of absolute configurations of molecules through comparisons between experimental and calculated ECD spectra [8,9]. 

**Figure 1:**
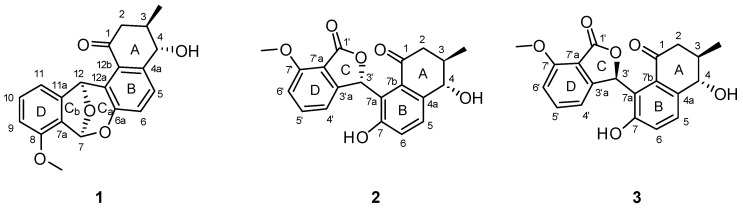
The structures of donghaecyclinones A–C (**1**–**3**).

Besides the application of computational methods for elucidating the structures of natural products, chemical investigations into understudied natural resources are also crucial in natural product research that aims to discover structurally and biologically novel compounds [10]. In this regard, we have been examining the chemical profiles of actinobacterial strains that inhabit the marine environments of volcanic islands, which may provide unique microbial habitats with volcanic minerals and salts from the surrounding seawater [11]. This has led to the successful discovery of various classes of new natural products [12,13,14,15,16,17,18] from volcanic island-derived marine actinomycetes. These include new cyclic peptides with anticancer and antituberculosis activities, ohmyungsamycins A and B [12], new anti-inflammatory linear polyketides, succinilenes A–D [17], and donghaesulfins A and B, which are dimeric benz[*a*]anthracenes linked through a sulfide bond [18]. By changing the culture conditions of the donghaesulfin-producing strain *Streptomyces* sp. SUD119, which was isolated from a marine sediment sample from a volcanic island (Ulleung Island) located in the middle of Donghae Sea of the Republic of Korea, we produced the new arranged angucyclinone metabolites donghaecyclinones A–C (**1**–**3**) (Figure 1). Large-scale fermentation of the SUD119 strain and further chromatographic purification resulted in the yield of **1**–**3** for subsequent spectroscopic analyses of these compounds. Here, we report the isolation, structural elucidation (in particular, the application of computational techniques for the establishment of configuration, including ECD calculations), and biological activities of donghaecyclinones A–C (**1**–**3**).

## 2. Results and Discussion

### 2.1. Structure Elucidation

Donghaecyclinone A (**1**) was obtained as a white powder of molecular formula C_20_H_18_O_5_ on the basis of high resolution-fast atom bombardment mass spectrometry (HR-FABMS) (obsd [M + H]^+^ at *m*/*z* 339.1227, calcd [M + H]^+^ at *m*/*z* 339.1231). The combination of ^1^H, ^13^C, and heteronuclear single quantum coherence (HSQC) NMR data (Table 1) of **1** in DMSO-*d*_6_ revealed that donghaecyclinone A (**1**) contains a carbonyl carbon (*δ*_C_ 198.9), five double-bond methine signals (*δ*_C_/*δ*_H_: 127.6/7.49, 131.5/7.27, 111.7/7.10, 122.2/7.00, and 111.5/6.94), and seven non-protonated double-bond carbons (*δ*_C_: 153.6, 148.5, 148.3, 140.4, 126.3, 125.9, and 122.9). Through further interpretation of the NMR data, we identified three *sp*^3^ oxymethines (*δ*_C_/*δ*_H_: 98.7/6.86, 77.3/6.68, and 71.4/4.22), a methoxy group (*δ*_C_/*δ*_H_: 55.6/3.85), one aliphatic methylene (*δ*_C_/*δ*_H_: 44.3/2.81 and 2.32), one aliphatic methine (*δ*_C_/*δ*_H_: 37.0/2.08), and one methyl group (*δ*_C_/*δ*_H_: 17.6/1.04). The molecular formula of **1** suggests that donghaecyclinone A (**1**) should possess 12 degrees of unsaturation. Because one carbonyl group and 12 double-bond carbons constituting six double bonds accounted for seven of the 12 degrees of unsaturation, donghaecyclinone A (**1**) must be a pentacyclic compound.

After establishing all of the ^1^H-^13^C one-bond correlations that were assigned by the analysis of the HSQC NMR spectrum, structural fragments of **1** were assembled by the interpretation of ¹H-¹H correlated spectroscopy (COSY) and heteronuclear multiple bond correlation (HMBC) NMR spectra. The COSY correlations from H_2_-2 (*δ*_H_ 2.81 and 2.32) to H-3 established the connection between C-2 (*δ*_C_ 44.3) and C-3 (*δ*_C_ 37.0). H-3 was found to be correlated with H_3_-3-Me (*δ*_H_ 1.04) and H-4 (*δ*_H_ 4.22), establishing that 3-Me (*δ*_C_ 17.6) and C-4 (*δ*_C_ 71.4) were bound to C-3. An additional COSY analysis verified the connection between H-4 and 4-OH (*δ*_H_ 5.61). The HMBC correlations from H_2_-2/H-3 to C-1 (*δ*_C_ 198.9), from H-3/H-4 to C-4a (*δ*_C_ 140.4), and from H-4 to C-12b (*δ*_C_ 126.3) established the first partial structure as a 4-hydroxy-3-methylcyclohexenone moiety (A ring). The characteristic vicinal coupling (*J* = 8.5 Hz) of H-5 and H-6 indicated that they were in a three-bond relationship in a six-membered aromatic ring, thus directly connecting C-5 and C-6. The three-bond ^1^H–^13^C couplings from H-5 to C-8a and C-12b, and from H-6 to C-4a and C-6a were assigned as a six-membered aromatic ring (B ring) composed of C-4a, C-5, C-6, C-6a, C-12a, and C-12b, which was then connected to the A ring as revealed by H-4/C-5, H-5/C-4, and H-4/C-12b HMBC correlations.

**Figure 2:**
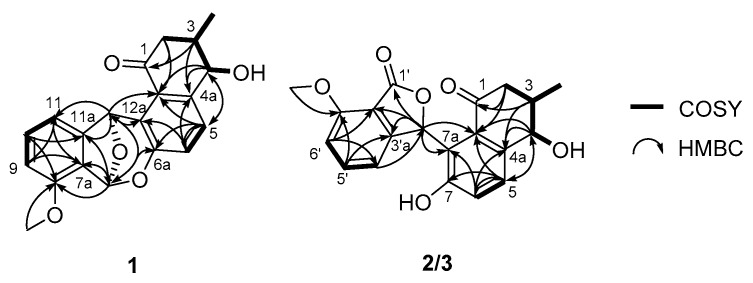
Determination of the planar structures of donghaecyclinones A–C (**1**–**3**) based on the analysis of key COSY and HMBC correlations.

An array of COSY correlations (H-9 (δ_H_ 6.94), H-10 (δ_H_ 7.27), and H-11 (δ_H_ 7.49)) and their coupling constants (*J* = 8.5 Hz each) clearly showed that C-9 (δ_C_ 111.5), C-10 (δ_C_ 131.5), and C-11 (δ_C_ 111.7) were connected in another six-membered aromatic spin system. The HMBC correlations from H-9 to C-7a (δ_C_ 122.8) and C-11, from H-10 to C-8 (δ_C_ 153.6) and C-11a (δ_C_ 148.3), and from H-11 to C-7a and C-9 constructed the D ring. The 8-OMe (δ_H_ 3.85) was shown to have an HMBC correlation with C-8, suggesting that the A ring is an anisole. Once the A, B, and D rings were constructed with 18 out of the 20 carbons in the molecular formula of donghaecyclinone A (**1**), these three rings were assembled based on HMBC correlations between the unused oxymethine protons (H-12 and H-7). H-12 exhibited ^2,3^*J*_CH_ couplings to C-11, C-11a, C-12a, and C-12b, establishing connectivity between the B and D rings through the C-12 oxymethine, which forms a seven-membered ring. The HMBC correlations from H-7 to C-6a, C-7a, C-8, and C-11a also connected the B and D rings through C-7. Due to the deshielded chemical shift (δ_C_ 98.7) of C-7, it was suggested that this carbon was dioxygenated. In addition, donghaecyclinone A, being a pentacyclic compound, requires one more ring. The last ring (C_b_ ring) was determined to be a tetrahydrofuran based on the H-7/C-12 and H-12/C-7 HMBC correlations, which also meets the requirement of two oxygen atoms for C-7, and constructed the dioxabicyclo[3.2.1]octadiene moiety. Therefore, the planar structure of donghaecyclinone A (**1**) was determined to be that of a 6/6/6/5/6 pentacyclic natural product (Figure 2).

Donghaecyclinone B (**2**) was isolated as a white powder. Its molecular formula was determined to be C_20_H_18_O_6_ based on the HR-FABMS (obsd [M − H]^−^ at *m*/*z* 353.1033, calcd [M − H]^−^ at *m*/*z* 353.1031). The 1-D and 2-D NMR spectroscopic data (Table 1) of **2** showed similar features to those of **1**, indicating that donghaecyclinone B is also a polyunsaturated aromatic natural product. Analysis of the ^1^H, ^13^C, and HSQC NMR data revealed the presence of two carbonyl carbons (δ_C_ 201.2 and 169.3), five aromatic methines (δ_C_/δ_H_: 136.2/7.54, 130.5/7.59, 122.0/7.03, 115.1/6.92, and 113.3/7.04), seven non-protonated aromatic carbons (δ_C_ 158.9, 157.5, 153.9, 139.9, 133.9, 122.9, and 116.0), two oxymethines (δ_C_/δ_H_: 76.4/ 7.43 and 74.4/4.55), one methoxy group (δ_C_/δ_H_: 56.1/3.93), one aliphatic methylene (δ_C_/δ_H_: 46.8/2.81 and 2.64), one aliphatic methine (δ_C_/δ_H_: 39.6/2.29), one methyl group (δ_C_/δ_H_: 18.9/1.20), and two hydroxy group protons (δ_H_ 8.83 and 4.69). Detailed comparison of the NMR data with those of **1** revealed that donghaecyclinone B contains an additional carbonyl carbon (δ_C_ 169.3) and hydroxy proton (δ_H_ 8.83) while it lacks the C-7 acetal methine carbon (δ_C_/δ_H_: 98.7/6.86) found in **1**. Based on its molecular formula, we determined that donghaecyclinone B bears 11 unsaturation equivalents. Two carbonyl functional groups and 12 double-bond signals comprising six double bonds correspond to seven degrees of unsaturation, indicating that donghaecyclinone B (**2**) is a tetracyclic compound. Further interpretation of the COSY and HMBC NMR spectral data of **2** established a 4-hydroxy-3-methylcyclohexenone moiety (A ring), a hydroxy benezene (B ring), and an anisole (D ring) composed of 18 carbons; the remaining two unassigned carbons were a carbonyl carbon (δ_C_ 169.3) and a oxymethine (δ_C_/δ_H_ 76.4/7.43). To accommodate these, the HMBC correlations from H-3′ (δ_H_ 7.43) to C-1′ (δ_C_ 169.3), C-3′a (δ_C_ 153.9), C-7′a (δ_C_ 116.0), C-7a (δ_C_ 122.9), and C-7b (δ_C_ 133.9) established γ-lactone as the last ring (C ring) and thus completed the tetracyclic planar structure of **2** (Figure 2).

Donghaecyclinone C (**3**) was obtained as a white powder along with **1** and **2**. The molecular formula was determined to be C_20_H_18_O_6_ on the basis of HR-FABMS (obsd [M − H]^−^ at *m/z* 353.1033, calcd [M − H]^−^ at *m/z* 353.1031). The molecular formula and the UV spectrum of **3** were identical to those of **2**, indicating that the structure of donghaecyclinone C (**3**) is similar to that of **2**. A comprehensive analysis of the 1D and 2D NMR data (Table 1) revealed that the planar structure of donghaecyclinone C (**3**) is identical to that of **2** (Figure 2). However, the optical rotations of **2** and **3** were found to have opposite signs with different absolute values (+125.6 and −156.7, respectively). This observation indicated that these two compounds were diastereomers, and not enantiomers, requiring rigorous stereochemical determination.

### 2.2. Determination of the Configurations of Donghaecyclinones A–C

Even though a number of angucycline/angucyclinone-class natural products were discovered to be representative polyketide type II metabolites from actinobacteria, mainly from the genus *Streptomyces* [19,20,21,22], they are often reported with undetermined configurations [23]. This is partly because the benzene ring in the middle of their structures makes it impossible to relate their relative configurations across all of the molecules. In addition, the absolute configurations of the rearranged angucyclinones (LS1924A and emycin D), such as donghaecyclinone A, that bear a dioxabicyclo[3.2.1]octadiene structure by rearrangement from ordinary angucyclinones have not been determined or have only been assigned by X-ray crystallography [23,24]. Furthermore, the stereochemistry of the rearranged angucyclinones that incorporate isobenzofuran, such as donghaecyclinones B and C, has not been rigorously studied [25]. Application of the DP4 or CP3 method would enable to relate the relative configurations, and utilization of electronic circular dichroism (ECD) calculations facilitates determination of the absolute configurations of angucyclinone-class compounds.

**Figure 3:**
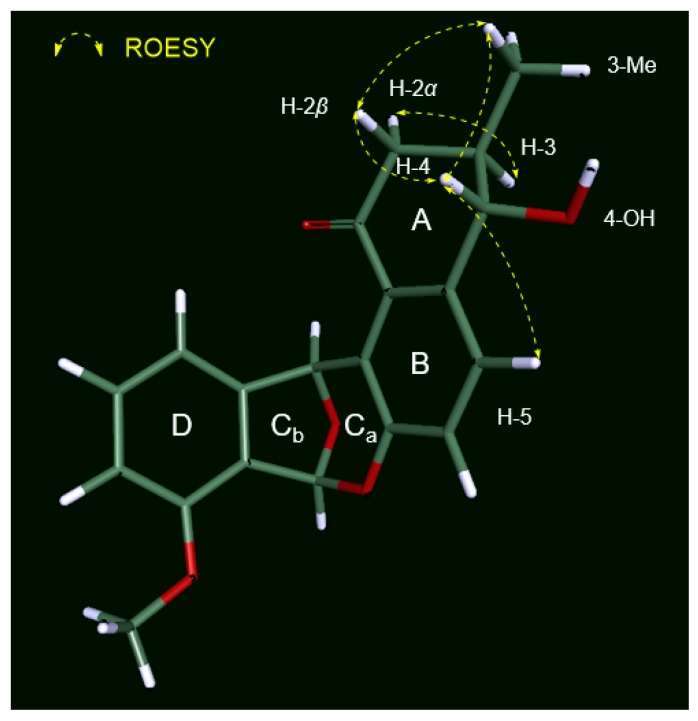
Key ROESY correlations of donghaecyclinone A (**1**).

**Figure 4:**
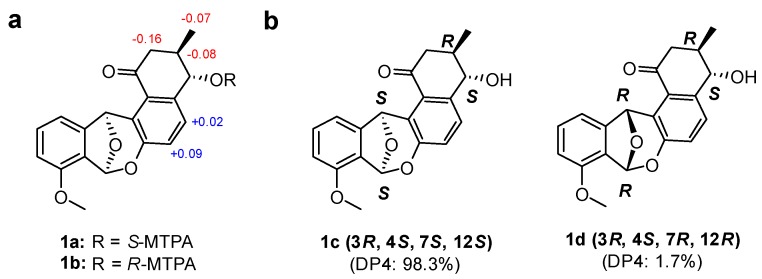
Determination of the absolute configuration of donghaecyclinone A (**1**). (**a**) Δ*δ_S-R_* values of the MTPA esters (**1a** and **1b**) in DMSO-*d*_6_. (**b**) the simulated DP4 models of the two possible diastereomers **1c**/**1d** (3*R*, 4*S*, 7*S*, and 12*S*/3*R*, 4*S*, 7*R*, and 12*R*) of **1**.

The relative configuration of **1** in ring A was established based on its ^3^*J*_HH_ values and rotating-frame Overhauser spectroscopy (ROESY) NMR spectroscopic data (Figure 3). The large ^1^H-^1^H coupling constant (7.5 Hz) between H-3 and H-4 strongly implied an *anti*-relationship between H-3 and H-4 and their axial orientation. The observed H-2*β*/H_3_-3-Me and H-2*β*/H-4 ROESY correlations assigned these protons on the same face in the A ring. The absolute configuration of the stereogenic center at C-4 was determined by a modified version of Mosher’s method by utilizing α-methoxy-α-trifluoromethylphenylacetic acid (MTPA) esterification and ^1^H NMR analysis (Figure 4a) [26]. Due to steric hindrance, additional *R*-MTPA-Cl was required to establish the *S*-MTPA ester (**1a**) in longer reaction time. Analysis of the ^1^H and 2D NMR spectroscopic data for the *S*- and *R*-MTPA esters (**1a** and **1b**) enabled us to calculate the Δ*δ_S_*_-*R*_ values; on this basis, we determined its absolute configuration to be 4*S*. Based on the relative relationship between C-3 and C-4, the absolute configuration of C-3 was also determined to be *R*.

However, the relative configuration of C-7 and C-12 at the junction of rings Ca and Cb could not be established because we did not observe any ROESY correlations between the A and Ca/Cb rings. To overcome this challenge, a quantum mechanics-based computational analysis using a DP4 statistical calculation was applied [6]. The two possible diastereomers **1c** (3*R*, 4*S*, 7*S*, and 12*S*) and **1d** (3*R*, 4*S*, 7*R*, and 12*R*) were proposed, and the ^1^H and ^13^C chemical shifts of the 12 conformers of **1c** and **1d** were calculated with their Boltzmann averaged populations. Based on a comparison of the experimental and calculated chemical shift values, our computational shielding tensor predicted diastereomer **1c** (3*R*, 4*S*, 7*S*, and 12*S* configurations) with 98.3% probability (Figure 4b). Finally, the absolute configuration of donghaecyclinone A (**1**) was proposed to be 3*R*, 4*S*, 7*S*, and 12*S*.

The relative configurations of the C-3 and C-4 positions in **2** and **3** were determined to be 3R* and 4S* based on analyses of the three-bond ^1^H-^1^H homonuclear coupling constants and ROESY correlations (Figures S23 and S24). In the analysis of NMR data, donghaecyclinones B and C (**2** and **3**) were expected to be diastereomers. Because both donghaecyclinones B and C possess the same 3R* and 4S* configurations and C-3′ in **2** and **3** is the only remaining chiral center with an undetermined relative configuration, these compounds must have opposite configurations at C-3′.

**Figure 5:**
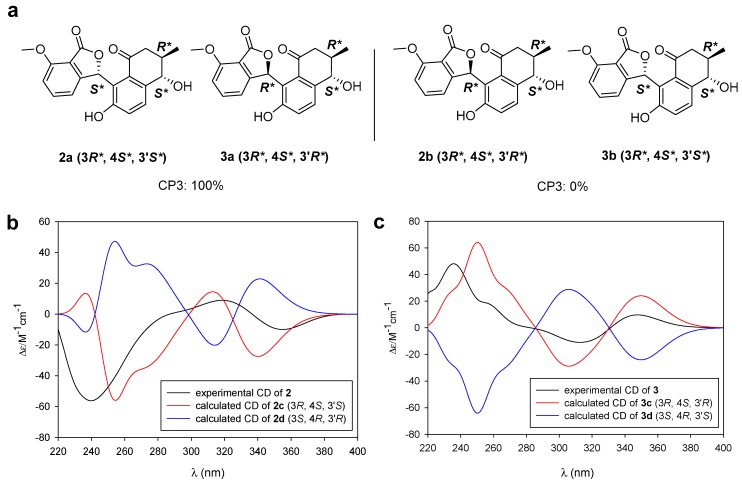
Determination of the relative and absolute configurations of donghaecyclinones B and C (**2** and **3**). (**a**) The simulated CP3 models of two possible diastereomer sets **2a**/**3a** (3R*, 4S*, and 3′S*/3R*, 4S*, and 3′R*) and **2b**/**3b** (3R*, 4S*, and 3′R*/3R*, 4S*, and 3′S*) on **2** and **3**. (**b**) Experimental and calculated ECD spectra of **2**, **2c**, and **2d**. (**c**) Experimental and calculated ECD spectra of **3**, **3c**, and **3d**.

For assignment of the relative configuration at C-3′, the two sets of possible diastereomers **2a**/**3a** and **2b**/**3b** were considered with 3R* and 4S* configurations (Figure 5a). In this case, the CP3 calculation—which was specially devised for the assignment of relative configuration to two plausible diastereomers from two sets of experimental NMR data—was applied instead of DP4 [5]. Our CP3 probability analysis of **2** and **3** along with the experimental and the calculated chemical shift values showed that the relative configurations of **2** and **3** were 3R*, 4S*, and 3′S* and 3R*, 4S*, and 3′R*, respectively, with 100% probability.

To determine the absolute configuration of **2** and **3**, we initially applied the modified version of Mosher’s method. However, during the MTPA derivatization, donghaecyclinones B and C (**2** and **3**) underwent isomerization at C-3′ at the furanone moiety, which prevented us from obtaining pure MTPA ester products. This problem was circumvented by the application of ECD calculation [27]. First, the energy-minimized conformers of **2c** (3*R*, 4*S*, and 3′*S*) and its enantiomer **2d** (3*S*, 4*R*, and 3′*R*) were calculated (Tables S6–S9). The ECD calculations of the two enantiomers (**2c** and **2d**) of **2** were performed using TD-DFT at the B3LYP/def-SVP//B3LYP/def-SVP level for all atoms. A comparison of the experimental ECD spectrum of **2** and the calculated ECD spectra of **2c** and **2d** showed that the experimental ECD spectrum of **2** is consistent with the calculated ECD spectrum of **2c**. Thus, we assigned **2** as having an absolute configuration of 3*R*, 4*S*, and 3′*S* (Figure 5b). The absolute configuration of **3** was determined to be 3*R*, 4*S*, and 3′*R* through a comparison of its experimental ECD spectrum with the calculated ECD spectra of two enantiomers (**3c** and **3d**) using the same procedure (Figure 5c).

### 2.3. Proposed Biosynthesis of Donghaecyclinones A–C

**Figure 6:**
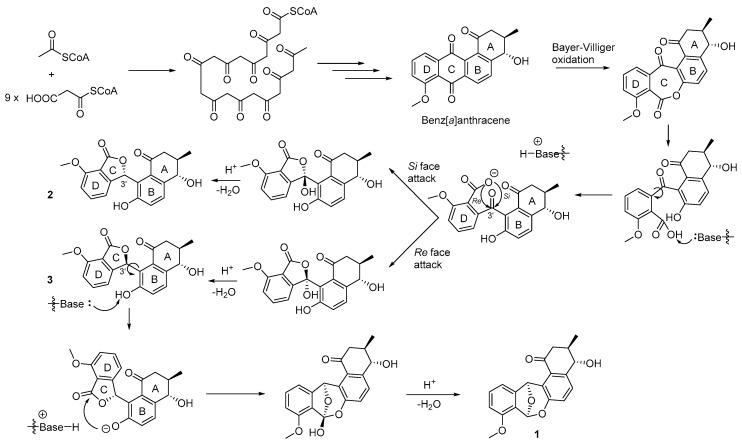
Proposed biosynthetic pathway of donghaecyclinones A–C (**1**–**3**).

The typical benz[*a*]anthracene structure of the angucycline/angucyclinone class metabolites is biosynthesized from acetyl CoA and nine malonyl CoA extender units through a type II polyketide synthase (PKS II) pathway [19,20,21,22]. From the common benz[*a*]anthracene precursor, the biosynthesis of the rearranged angucyclinones, donghaecyclinones A–C (**1**–**3**), was proposed (Figure 6). The C ring of benz[*a*]anthracene can be cleaved through Bayer-Villiger oxidation and hydrolysis of the ester linkage. Then the deprotonated carboxylate anion in the D ring attacks the carbonyl carbon at C-3′ position of donghaecyclinones B and C (**2** and **3**) followed by dehydration to furnish **2** and **3**. During the C ring formation, both *Si* and *Re* face attacks may occur, resulting in the production of the 3′-epimeric structures of donghaecyclinones B and C (Figure 6). These compounds were obviously observed as natural products in the bacterial culture of before fractionation (Figure S29). Donghaecyclinone A (**1**) could be derived from **3** to form the dioxabicylo[3.2.1]octadiene structure by the nucleophilic attack to the ester carbonyl group in the C ring by the phenolic oxygen in the B ring and dehydration (Figure 6).

### 2.4. Biological Activities of Donghaecyclinones A–C

The biological activities of the angucycline/angucyclinone-class natural products are known to include cytotoxicity against various cancer cell lines and antibacterial activity [15,16,17]. Donghaecyclinones A–C (**1**–**3**) were evaluated for antibacterial activity against Gram-positive *Staphylococcus aureus* ATCC 25923, *Enterococcus faecalis* ATCC 19433, *Enterococcus faecium* ATCC 19434, Gram-negative *Klebsiella pneumoniae* ATCC 10031, *Salmonella enterica* ATCC 14028, and *Escherichia coli* ATCC 25922 using ampicillin as a positive control. In this assay, none of the donghaecyclinones displayed significant inhibitory activity [MIC > 100 μg/mL]. The antifungal activity of **1**–**3** against *Aspergillus fumigatus* HIC 6094, *Trichophyton rubrum* NBRC 9185, *Trichophyton mentagrophytes* IFM 40996, and *Candida albicans* ATCC 10231 was measured, and the donghaecyclinones were not found to inhibit the growth of these pathogenic fungi [MIC > 100 μg/mL]. In the cytotoxicity assay against five cancer cell lines—HCT116 (a colon cancer cell line), MDA-MB231 (a breast cancer cell line), SNU638 (a gastric carcinoma cell line), A549 (a lung cancer cell line), and SK-HEP1 (a liver cancer cell line)—donghaecyclinone C displayed significant inhibitory activity (IC_50_ = 6.0–9.6 μM) while donghaecyclinones A and B exhibited lower cytotoxicity (IC_50_ = 9.6–28.9 μM) (Table 2, Figure S30). Donghaesulfins A and B, the dimeric-angucyclinones reported by our previous work with the donghaecyclinone-producing bacterial strain (*Streptomyces* sp. SUD119), did not show remarkable cytotoxicity [14], possibly indicating that dimerization provides negative effects on the angucyclinone class compounds. Based on the difference of the cytotoxicity between donghaecyclinones B and C and their structures, the C-3′ stereogenic center apparently plays a significant role in the cytotoxicity of isobenzofuran-bearing rearranged angucyclinone metabolites. However, the mechanism causing the difference in cytotoxicity is unknown and requires further studies.

## 3. Experimental

### 3.1. General Experimental Procedures

Optical rotations were obtained using a JASCO P-200 polarimeter (JASCO, Easton, PA, USA) with a sodium light source and a 1 cm cell. UV spectra were acquired on a Perkin Elmer Lambda 35 UV/Vis spectrophotometer (Perkin Elmer, Waltham, MA, USA). IR spectra were acquired using a Thermo Nicolet iS10 detector (Thermo, Madison, CT, USA). ECD spectra were recorded on an Applied Photophysics Chirascan-plus circular dichroism spectrometer. ^1^H, ^13^C, and 2D NMR spectra were acquired on Bruker Avance 600 MHz and 850 MHz spectrometers (Bruker, Billerica, MA, USA) at the National Center for Inter-University Research Facilities (NCIRF) at Seoul National University. High-resolution fast atom bombardment (HR-FAB) mass spectra were recorded using a Jeol JMS-600W high-resolution mass spectrometer (Jeol, München, Germany) at the NCIRF. LC/MS data were obtained on an Agilent Technologies 6130 quadrupole mass spectrometer (Agilent Technologies, Santa Clara, CA, USA) coupled with an Agilent Technologies 1200 series HPLC.

### 3.2. Cultivation and Extraction

The isolation and phylogenetic analysis of the *Streptomyces* sp. bacterial strain SUD119 were previously reported [18]. Strain SUD119 was inoculated in 50 mL of YEME medium (4 g of yeast extract, 10 g of malt extract, and 4 g of glucose in 1 L of artificial seawater) in a 125 mL Erlenmeyer flask. The bacterial strain was cultivated for 3 days on a rotary shaker at 160 rpm at 30 °C. The liquid culture (10 mL) was inoculated directly into 1 L of A1 liquid medium (4 g of yeast extract, 10 g of starch, and 4 g of peptone in 1 L of artificial seawater) in 2.8 L Fernbach flasks (1 L in each of 12 flasks for a total volume of 12 L). After cultivation for eight days, the 12 L culture of the SUD119 strain was extracted using 18 L of EtOAc. The EtOAc layer was separated from the water layer. To remove residual water, anhydrous sodium sulfate was added to the EtOAc layer. The cultivation and extraction procedures were repeated 12 times (total culture volume: 144 L). The EtOAc extract was dried in vacuo, and 12 g of dry extract material was obtained.

### 3.3. Isolation of Donghaecyclinones A–C (***1***–***3***)

The dried extract of the SUD119 strain was redissolved in MeOH and filtered through a syringe filter (PVDF). The filtered extract was then injected directly onto a semipreparative reversed-phase high performance liquid chromatography (HPLC) column (Kromasil C_18_ (2): 250 mm × 10 mm, 5 μm), and the material was separated with a gradient solvent system (20% MeOH/H_2_O to 80% MeOH/H_2_O over 40 min and 80% MeOH/H_2_O for 40 min, UV detection at 280 nm, flow rate: 2 mL/min). Two major peaks at retention times of 20.4 and 38.6 min were observed. These major fractions were analyzed by liquid chromatography/mass spectrometry (LC/MS). The fraction collected at 20.4 min contained donghaecyclinones B and C (**2** and **3**), and the other major fraction obtained at 38.6 min was composed of pure donghaecyclinone A (**1**, 4.2 mg). The mixture of donghaecyclinones B and C (**2** and **3**) was further purified using an isocratic HPLC solvent system (48% MeOH/H_2_O, UV detection at 280 nm, flow rate: 2 mL/min) using a reversed-phase C_18_ HPLC column (Kromasil C_18_ (2): 250 mm × 10 mm, 5 μm). Donghaecyclinones B and C (**2** and **3**) were acquired at retention times of 28.2 min (3.5 mg) and 31.6 min (4.1 mg), respectively.

Donghaecyclinone A (**1**): White powder; ${\left[\alpha \right]}_{D}^{25}$ −6.9 (*c* 0.5, MeOH); UV (MeOH) λ_max_ (log *ε*) 210 (4.32), 260 (3.86), 325 (3.58) nm; IR (neat) *ν*_max_ 3282, 2971, 1682, 1577, 1517 cm^−1^; ^1^H and ^13^C NMR data, Table 1; HR-FABMS *m*/*z* 339.1227 [M + H]^+^ (calcd for C_20_H_19_O_5_, 339.1231).

Donghaecyclinone B (**2**): White powder; ${\left[\alpha \right]}_{D}^{25}$ −125.6 (*c* 0.5, MeOH); UV (MeOH) λ_max_ (log ε) 210 (4.21), 300 (3.62), 320 (3.46) nm; ECD (*c* 4.2 × 10^−4^ M, MeOH) λ_max_ (Δ*ε*) 238 (−56.4) nm, 286 (+0.7) nm, 318 (+8.6) nm, 354 (−9.9) nm; IR (neat) *ν*_max_ 3338, 2961, 1752, 1672, 1607, 1487 cm^−1^; ^1^H and ^13^C NMR data, Table 1; HR-FABMS *m*/*z* 353.1033 [M − H]^−^ (calcd for C_20_H_17_O_6_, 353.1031).

Donghaecyclinone C (**3**) White powder; ${\left[\alpha \right]}_{D}^{25}$ +156.7 (*c* 0.5, MeOH); UV (MeOH) λ_max_ (log ε) 210 (4.27), 300 (3.65), 320 (3.44) nm; ECD (*c* 4.2 × 10^−4^ M, MeOH) λ_max_ (Δ*ε*) 235 (+48.0) nm, 255 (+18.8) nm, 288 (−1.7) nm, 315 (−11.2) nm, 345 (+9.6) nm; IR (neat) *ν*_max_ 3363, 2964, 1752, 1673, 1607, 1485 cm^−1^; ^1^H and ^13^C NMR data, Table 1; HRFABMS *m*/*z* 353.1033 [M − H]^−^ (calcd for C_20_H_17_O_6_, 353.1031).

### 3.4. MTPA Esterification of Donghaecylinone A (***1***)

Donghaecyclinone A (**1**) was placed into two 40 mL vials (1 mg for each) and dried for 18 h under high vacuum. A catalytic amount of crystalline dimethylaminopyridine (DMAP) was then added to each of the vials containing **1**. Distilled anhydrous pyridine (1 mL) was added to each vial under Ar. The reaction mixtures were stirred at room temperature for 5 min. Then, 20 µL of *S*- or *R*-α-methoxy trifluoromethylphenylacetic acid (MTPA) chloride was added to each vial. The reaction with *S*-MTPA-Cl was maintained with stirring for 3 h at room temperature and then quenched by the addition of 50 µL of MeOH, furnishing the *R*-MTPA ester (**1b**) of **1**. To yield the *S*-MTPA ester (**1a**), the reaction mixture was stirred at room temperature for 5 h with an additional amount of *R*-MTPA-Cl (20 µL) to facilitate esterification, and then quenched by the addition of 50 µL of MeOH. The reaction products (**1a** and **1b**) were isolated by HPLC using gradient elution conditions (40% to 100% aqueous CH_3_CN over 20 min, a reversed-phase C_18_ column (Kromasil C_18_ (2): 250 mm × 10 mm, 5 μm), flow rate: 2 mL/min, UV detection at 280 nm). The *S-* and *R*-MTPA esters (**1a** and **1b**) of **1** eluted at 33.4 and 32.6 min, respectively. The Δδ*_S_*_-*R*_ values of the signals around stereogenic centers of the MTPA esters were assigned based on analysis of the ^1^H and ^1^H–^1^H COSY NMR spectra.

#### 3.4.1. The *S*-MTPA Ester (**1a**) of Donghaecyclinone A (**1**)

^1^H NMR (800 MHz, DMSO-*d*_6_) *δ* 7.50–7.44 (m, 5H), 7.31 (t, *J* = 8.0, 1H), 7.30 (d, *J* = 8.0, 1H), 7.12 (d, *J* = 8.0, 1H), 7.06 (d, *J* = 8.0, 1H), 6.96 (d, *J* = 8.0, 1H), 6.90 (s, 1H), 6.65 (s, 1H), 6.04 (d, *J* = 5.0, 1H), 3.85 (s, 3H), 3.45 (s, 3H), 2.76 (dd, *J* = 16.0, 4.5, 1H), 2.58 (m, 1H), 2.44 (dd, *J* = 16.0, 6.0, 1H), 0.87 (d, *J* = 6.5, 3H). The molecular formula of **1a** was determined to be C_30_H_25_F_3_O_7_ ([M + Na]^+^ at *m*/*z* 577).

#### 3.4.2. The *R*-MTPA Ester (**1b**) of Donghaecyclinone A (**1**)

^1^H NMR (800 MHz, DMSO-*d*_6_) *δ* 7.48–7.43 (m, 5H), 7.29 (t, *J* = 8.0, 1H), 7.13 (d, *J* = 8.0, 1H), 7.11c (d, *J* = 8.0, 1H), 6.97 (d, *J* = 8.0, 1H), 6.96 (d, *J* = 8.0, 1H), 6.89 (s, 1H), 6.63 (s, 1H), 6.04 (d, *J* = 5.0, 1H), 3.85 (s, 3H), 3.50 (s, 3H), 2.98 (dd, *J* = 16.0, 4.5, 1H), 2.66 (m, 1H), 2.52 (dd, *J* = 16.0, 6.0, 1H), 0.94 (d, *J* = 6.5, 3H). The molecular formula of **1b** was determined to be C_30_H_25_F_3_O_7_ ([M + Na]^+^ at *m*/*z* 577).

### 3.5. DP4 and CP3 Analyses

A conformational search was performed using the MacroModel (Version 9.9, Schrödinger LLC, New York, NY, USA) program in Maestro (Version 9.9, Schrödinger LLC) with a mixed torsional/low-mode sampling method. Conformers of diastereomers within 10 kJ/mol, as calculated by the MMFF force field, were selected. The geometries of the conformers were calculated for optimization at the B3LYP/6-31G++ level in gas phase. The shielding tensor values of the optimized conformers were calculated on the basis of the equation below, where $\delta_{calc}^{x}$ is the calculated NMR chemical shift for nucleus x, and $\sigma^{o}$ is the shielding tensor for the proton and carbon nuclei calculated at the B3LYP/6-31++ level. These values were averaged via the Boltzmann population with the associated Gibbs free energy and utilized for the DP4 and CP3 analyses, which were facilitated by an Excel sheet provided by the original authors.

(1) $$\delta_{calc}^{x}=\frac{\sigma^{o}-\sigma^{x}}{1-\sigma^{o}/10^{6}}$$

### 3.6. ECD Calculation

The ground-state geometries were computed with density functional theory (DFT) calculations using Turbomole 6.5, the basis set def-SVP for all atoms, and the B3LYP/DFT functional level. The ground states were further confirmed using a harmonic frequency calculation. The calculated ECD data corresponding to the optimized structures were acquired with TD-DFT at the B3LYP/DFT functional level using the basis set def-SVP for all atoms. The CD spectra were simulated by overlapping for each transition according to the equation below, where *σ* is the width of the band at the height of 1/*e* and Δ*E_i_* and *R_i_* are the excitation energies and rotatory strengths for transition *i*, respectively. In the present work, the value of *σ* was taken to be 0.10 eV.

(2) $$\Delta \epsilon \left(E\right)=\frac{1}{2.297\times 10^{-39}}\frac{1}{\sqrt{2\pi \sigma }}\overset{A}{\underset{i}{\sum }}\Delta E_{i}R_{i}e^{[-{\left(E-\Delta E_{i}\right)}^{2}/\left(2\sigma {)}^{2}\right]}$$

### 3.7. Antibacterial Activity Bioassay

The inhibitory activities of donghaecyclinones A–C (**1**–**3**) were evaluated against Gram-positive bacteria (*Staphylococcus aureus* ATCC 25923, *Bacillus subtilis* ATCC 6633, *Streptococcus pyogenes* ATCC 19615, and *Kocuria rhizophila* NBRC 12708) and Gram-negative bacteria (*Klebsiella pneumoniae* ATCC 10031, *Salmonella enterica* ATCC 14028, *Escherichia coli* ATCC 25922, and *Proteus hauseri* NBRC) using the previously reported method [18].

### 3.8. Antifungal Activity Bioassay

*Trichophyton mentagrophytes* IFM 40996, *Trichophyton rubrum* NBRC 9185, *Aspergillus fumigatus* HIC 6094, and *Candida albicans* ATCC 10231 strains were used to measure the antifungal activities of donghaecyclinones A–C (**1**–**3**) by following the previously reported procedure [18].

### 3.9. Cytotoxicity Assay

The cytotoxicity of donghaecyclinones A–C (**1**–**3**) were evaluated using a sulforhodamine B (SRB) assay as previously reported [14]. The five human cancer cell lines A549, MDA-MB231, HCT116, SNU638, and SK-HEP1 were tested with etoposide as a positive control.

## 4. Conclusions

We discovered donghaecyclinones A–C (**1**–**3**) in *Streptomyces* sp. strain SUD119, which was isolated from a sample of marine sediment collected from the volcanic island (Ulleung Island) in the Republic of Korea. Donghaecyclinone A possesses a pentacyclic skeleton with a dioxabicyclo[3.2.1]octadiene structure derived from benz[*a*]anthracene, the typical structure of angucyclinone-class natural products. Donghaecyclinones B and C possess an isobenzofuran moiety that is also a rearrangement of benz[*a*]anthracene. The absolute configurations of **1**–**3** were fully established by computational methods utilizing NMR shielding tensor and ECD along with a modified version of Mosher’s method. To date, the configurations of angucyclinones with a dioxabicyclo[3.2.1]octadiene moiety have remained undetermined or have been established occasionally by X-ray crystallography. The stereochemistry of rearranged angucyclinones that bear isobenzofuran has not been rigorously examined. This configurational analysis of these rearranged angucyclinone-class compounds by quantum mechanics-based computational tools constitutes a general method for the structural characterization of rearranged angucyclinones and related natural products.

## Figures and Tables

**Table 1:** ^1^H and ^13^C NMR data for **1**–**3**.

	1 ^a^				2 ^b^			3 ^b^		
C/H	δ_H_^c^	mult (*J* in Hz)	δ_C_^c^	C/H	δ_H_^c^	mult (*J* in Hz)	δ_C_^c^	δ_H_^c^	mult (*J* in Hz)	δ_C_^c^
**1**			198.9, s	**1**			201.2, s			200.4, s
**2*α***	2.81	dd (16.0, 4.5), 1H	44.3, t	**2*α***	2.81	dd (15.0, 4.5), 1H	46.8, t	2.99	dd (15.0, 4.5), 1H	45.6, t
**2*β***	2.32	dd (16.0, 12.0), 1H		**2*β***	2.64	dd (15.0, 10.0), 1H		2.38	dd (15.0, 10.0), 1H	
**3**	2.08	m, 1H	37.0, d	**3**	2.29	m, 1H	39.6, d	2.24	m, 1H	38.2, d
**3-**	1.04	d (6.5), 3H	17.6, q	**3-**	1.20	d (6.5), 3H	18.9, q	1.18	d (6.5), 3H	18.0, q
**Me**				**Me**						
**4**	4.22	dd (7.5, 6.0), 1H	71.4, d	**4**	4.55	d (6.5), 1H	74.4, d	4.43	d (6.5), 1H	73.4, d
**4-**	5.61	d (6.0), 1H		**4-**	4.69	brs, 1H		4.74	brs, 1H	
**OH**				**OH**						
**4a**			140.4, s	**4a**			139.9, s			140.1, s
**5**	7.49	d (8.5), 1H	127.6, d	**5**	7.59	d (8.5), 1H	130.5, d	7.63	d (8.5), 1H	129.4, d
**6**	7.00	d (8.5), 1H	122.2, d	**6**	7.03	d (8.5), 1H	122.0, d	7.05	d (8.5), 1H	121.9, d
**6a**			148.5, s	**7**			157.5, s			157.3, s
**7**	6.86	s, 1H	98.7, d	**7-**	8.83	brs, 1H		8.80	brs, 1H	
**7a**			122.8, s	**OH**						
**8**			153.6, s	**7a**			122.9, s			122.8, s
				**7b**			133.9, s			132.7, s
**8-**	3.85	s, 3H	55.6, q	**1′**			169.3, s			169.3, s
**OMe**				**3′**	7.43	s, 1H	76.4, d	7.41	s, 1H	76.5, d
**9**	6.94	t (8.5), 1H	111.5, d	**3′a**			153.9, s			153.8, s
**10**	7.27	t (8.5), 1H	131.5, d	**4′**	6.92	d (8.5), 1H	115.1, d	6.93	d (8.5), 1H	115.0, d
**11**	7.10	d (8.5), 1H	111.7, d	**5′**	7.54	t (8.5), 1H	136.2, d	7.54	t (8.5), 1H	136.1, d
**11a**			148.3, s	**6′**	7.04	d (8.5), 1H	111.3, d	7.04	d (8.5), 1H	111.2, d
**12**	6.68	s, 1H	77.3, d	**7′**			158.9, s			158.8, s
**12a**			125.9, s	**7′a**			116.0, s			115.8, s
**12b**			126.3, s	**7′-**	3.93	s, 3H	56.1, q	3.94	s, 3H	56.0, q
				**OMe**						

^a^ DMSO-*d*_6_, ^b^ acetone-*d*_6_, ^c 1^H, and ^13^C NMR spectra were recorded at 600 and 150 MHz, respectively.

**Table 2:** The cytotoxicity assay for donghaecyclinones A–C (**1**–**3**).

	Cytotoxicity (IC_50_ μM)				
	HCT116	MDA-MB231	SNU638	A549	SK-HEP1
**1**	28.9	20.0	16.1	22.9	14.2
**2**	27.3	19.3	19.6	19.0	9.6
**3**	8.0	6.7	9.5	9.6	6.0
Etoposide	0.4	0.5	0.4	0.5	0.6

## Supplementary Materials

The following are available online at https://www.mdpi.com/1660-3397/18/2/121/s1. Figures S1–S6. 1D and 2D NMR spectra of donghaecyclinone A (**1**); Figure S7. IR spectrum of donghaecyclinone A (**1**); Figure S8: ^1^H NMR spectrum (800 MHz) of *S*-MTPA ester (**1a**); Figure S9: COSY spectrum (800 MHz) of *S*-MTPA ester (**1a**); Figure S10. ^1^H NMR spectrum (800 MHz) of *R*-MTPA ester (**1b**); Figure S11. COSY spectrum (800 MHz) of *R*-MTPA ester (**1b**); Figures S12–S18. 1D and 2D spectra of donghaecyclinone B (**2**); Figure S19. IR spectrum of donghaecyclinone B (**2**); Figures S20–S25: 1D and 2D NMR spectra of donghaecyclinone B (**3**); Figure S26: IR spectrum of donghaecyclinone C (**3**); Figure S27. Key ROESY correlations of donghaecyclinone B (**2**); Figure S28: Key ROESY correlations of donghaecyclinone C (**3**); Figure S29: LC/MS chromatogram of the extract of the bacterial strain *Streptomyces* sp. SUD119 and purified donghaecyclinones A–C (**1**–**3**). Figure S30: Cytotoxicity result of donghaecyclinones A–C (**1**–**3**); Tables S1–S3. The major conformers of diastereomers **1c**–**1d**, **2a**, **2b**, **3a**, and **3b** identified by conformational searches in MMFF94 force field using MacroModel; Table S4. Experimental (Exp.) and calculated (Cal.) chemical shift values (CS, δ) of diastereomers **1c**–**1d** on donghaecyclinone A (**1**); Table S5. Experimental (Exp.) and calculated (Cal.) chemical shift values (CS, δ) of diastereomers **2a/3b**–**2b**/**3a** on donghaecyclinones B and C (**2** and **3**): Cartesian coordinates of the conformers shown in Tables S4 and S5; Tables S6–S9. ECD calculation of **2c**, **2d**, **3c**, and **3d**.
