# Supplementary material for: Donghaecyclinones A–C: New Cytotoxic Rearranged Angucyclinones from a Volcanic Island-Derived Marine Streptomyces sp
Source: Mar Drugs. 2020 Feb 18;18(2):121. doi: 10.3390/md18020121 (PMC7073551; doi:10.3390/md18020121)
Supplement: Supplementary file 1 [file marinedrugs-18-00121-s001.pdf]

Supplementary Materials

Donghaecyclinones A–C: New Cytotoxic Rearranged Angucyclinones from a Volcanic Island-Derived Marine *Streptomyces* sp.

Munhyung Bae[1], Joon Soo An[1], Seong-Heon Hong[1], Eun Seo Bae[1], Beomkoo Chung[2], Yun Kwon[1], Suckchang Hong[3], Ki-Bong Oh[2], Jongheon Shin[1], Sang Kook Lee[1], and Dong-Chan Oh[1,*]

[1] Natural Products Research Institute, College of Pharmacy, Seoul National University, Seoul 08826, Republic of Korea; baemoon89@snu.ac.kr (M.B.); ahnjunsoo@snu.ac.kr (J.S.A.); sung954@snu.ac.kr (S.-H.H.); ddol1289@snu.ac.kr (E.S.B.); kisi2016@snu.ac.kr (Y.K.); sklee61@snu.ac.kr (S.K.L.); shinj@snu.ac.kr (J.S.)

[2] Department of Agricultural Biotechnology, College of Agriculture & Life Sciences, Seoul National University, Seoul 08826, Republic of Korea; beomkoo01@snu.ac.kr (B.C.); ohkibong@snu.ac.kr (K.-B.O.)

[3] Research Institute of Pharmaceutical Sciences, College of Pharmacy, Seoul National University, Seoul 08826, Republic of Korea; schong17@snu.ac.kr (S.H.)

* Correspondence: dongchanoh@snu.ac.kr; Tel.: +82-2880-2491

Table of Contents

Figure S1. ^{1}H NMR spectrum (600 MHz) of donghaecyclinone A (**1**) in DMSO–d_6.
Figure S2. ^{13}C NMR spectrum (150 MHz) of donghaecyclinone A (**1**) in DMSO–d_6.
Figure S3. COSY spectrum (600 MHz) of donghaecyclinone A (**1**) in DMSO–d_6.
Figure S4. HSQC spectrum (600 MHz) of donghaecyclinone A (**1**) in DMSO–d_6.
Figure S5. HMBC spectrum (600 MHz) of donghaecyclinone A (**1**) in DMSO–d_6.
Figure S6. ROESY spectrum (600 MHz) of donghaecyclinone A (**1**) in DMSO–d_6.
Figure S7. IR spectrum of donghaecyclinone A (**1**).
Figure S8. ^{1}H NMR spectrum (800 MHz) of *S*-MTPA ester (**1a**) of **1** in DMSO–d_6.
Figure S9. COSY spectrum (800 MHz) of *S*-MTPA ester (**1a**) of **1** in DMSO–d_6.
Figure S10. ^{1}H NMR spectrum (800 MHz) of *R*-MTPA ester (**1b**) of **1** in DMSO–d_6.
Figure S11. COSY spectrum (800 MHz) of *R*-MTPA ester (**1b**) of **1** in DMSO–d_6.
Figure S12. ^{1}H NMR spectrum (600 MHz) of donghaecyclinone B (**2**) in acetone–d_6.
Figure S13. ^{13}C NMR spectrum (150 MHz) of donghaecyclinone B (**2**) in acetone–d_6.
Figure S15. COSY spectrum (600 MHz) of donghaecyclinone B (**2**) in acetone–d_6.
Figure S16. HSQC spectrum (600 MHz) of donghaecyclinone B (**2**) in acetone–d_6.
Figure S17. HMBC spectrum (600 MHz) of donghaecyclinone B (**2**) in acetone–d_6.
Figure S18. ROESY spectrum (600 MHz) of donghaecyclinone B (**2**) in acetone–d_6.
Figure S19. IR spectrum of donghaecyclinone B (**2**).
Figure S20. ^{1}H NMR spectrum (600 MHz) of donghaecyclinone B (**3**) in acetone–d_6.
Figure S21. ^{13}C NMR spectrum (150 MHz) of donghaecyclinone C (**3**) in acetone–d_6.
Figure S22. COSY spectrum (600 MHz) of donghaecyclinone C (**3**) in acetone–d_6.
Figure S23. HSQC spectrum (600 MHz) of donghaecyclinone C (**3**) in acetone–d_6.
Figure S24. HMBC spectrum (600 MHz) of donghaecyclinone C (**3**) in acetone–d_6.
Figure S25. ROESY spectrum (600 MHz) of donghaecyclinone C (**3**) in acetone–d_6.
Figure S26. IR spectrum of donghaecyclinone C (**3**).
Figure S27. Key ROESY correlations of donghaecyclinone B (**2**).
Figure S28. Key ROESY correlations of donghaecyclinone C (**3**).
Figure S29. LC/MS chromatogram of the extract of the bacterial strain Streptomyces sp. SUD119 and purified donghaecyclinones A-C (**1**-**3**).
Figure S30. Cytotoxicity result of donghaecyclinones A-C (**1**-**3**) againt five cancer cell lines.
Table S1. The major conformers of diastereomers **1c**–**1d** on donghaecyclinone A (**1**) identified by conformational searches in MMFF94 force field using MacroModel.
Table S2. The major conformers of diastereomers **2a**/**3b** identified by conformational searches in MMFF94 force field using MacroModel.
Table S3. The major conformers of diastereomers **2b**/**3a** identified by conformational searches in MMFF94 force field using MacroModel.
Table S4. Experimental (Exp.) and calculated (Cal.) chemical shift values (CS, δ) of diastereomers **1c**–**1d** on donghaecyclinone A (**1**).
Table S5. Experimental (Exp.) and calculated (Cal.) chemical shift values (CS, δ) of

diastereomers **2a/3b**–**2b**/**3a** on donghaecyclinones B and C (**2** and **3**).
Cartesian coordinates of the conformers shown in Table S4.
Cartesian coordinates of the conformers shown in Table S5.
Table S6. ECD calculation of donghaecyclinone B (**2c**).
Table S7. ECD calculation of *ent*-donghaecyclinone B (**2d**).
Table S8. ECD calculation of donghaecyclinone C (**3c**).
Table S9. ECD calculation of *ent*-donghaecyclinone C (**3d**).

Figure S1. ^{1}H NMR spectrum (600 MHz) of donghaecyclinone A (**1**) in DMSO–d_6.

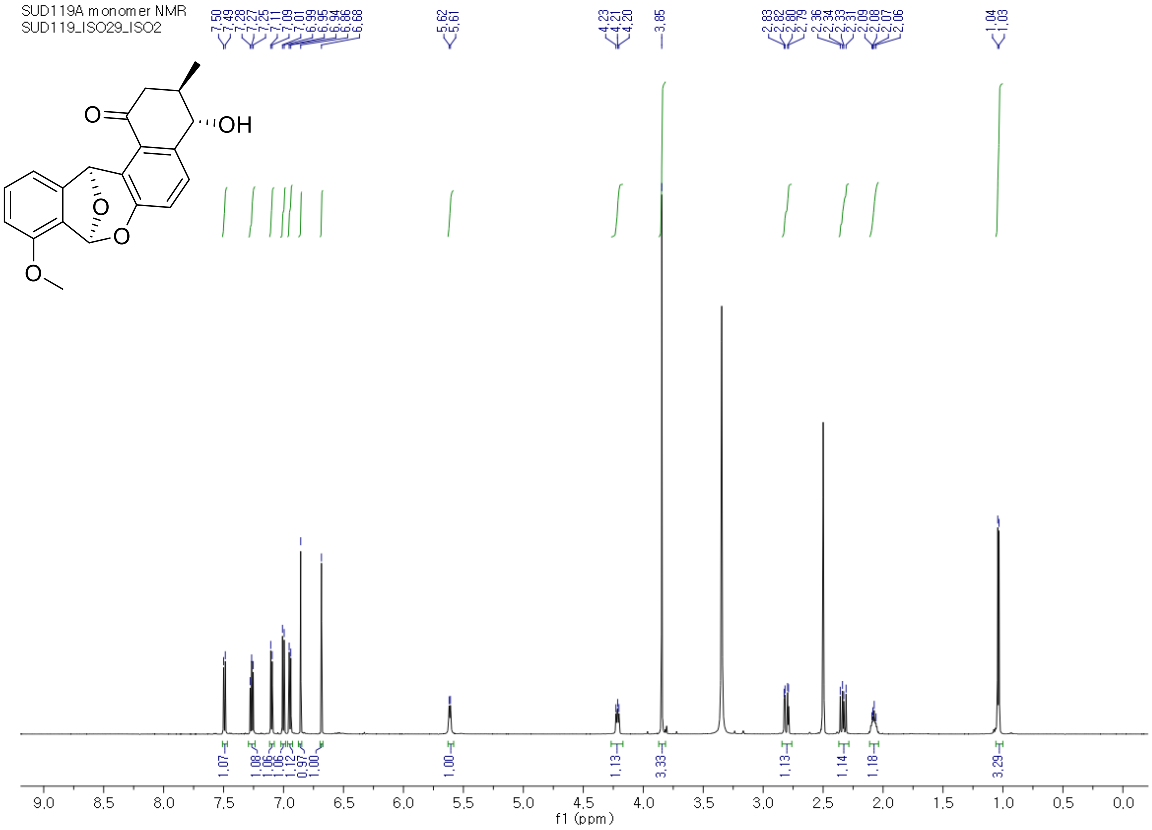

Figure S2. ^{13}C NMR spectrum (150 MHz) of donghaecyclinone A (**1**) in DMSO–d_6.

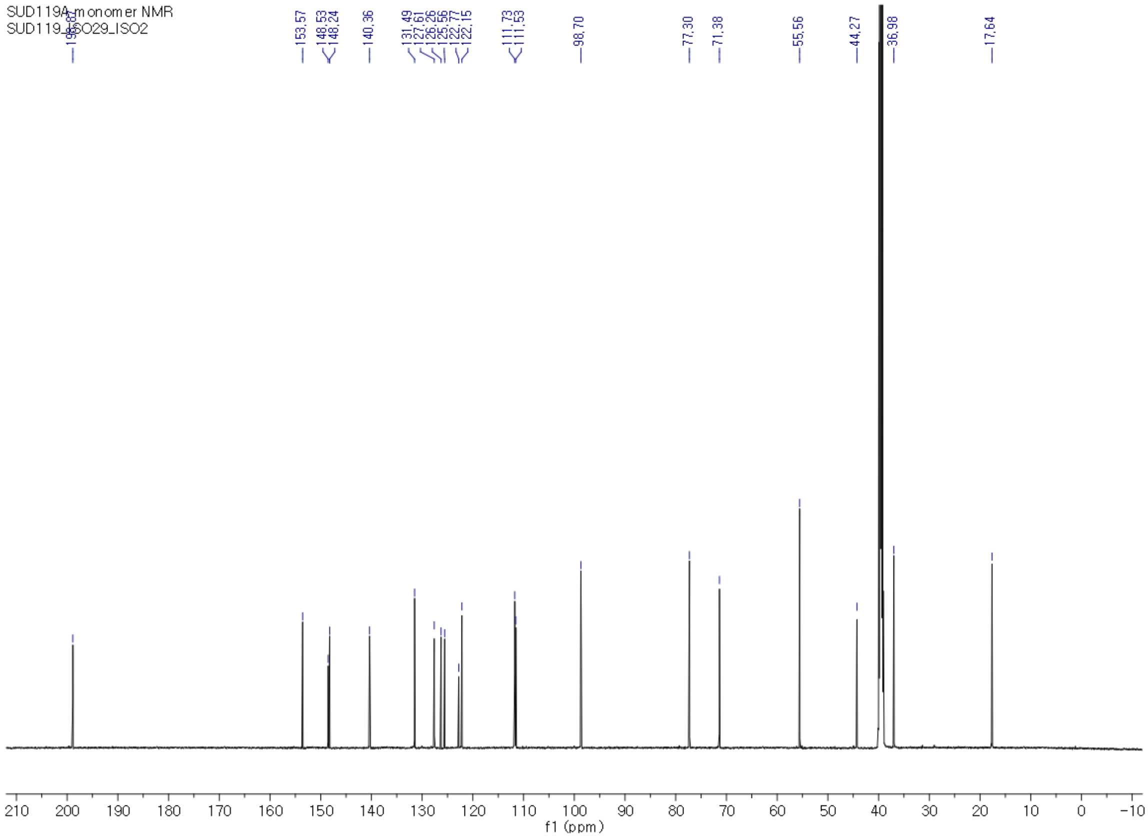

Figure S3. COSY spectrum (600 MHz) of donghaecyclinone A (**1**) in DMSO–d_6.

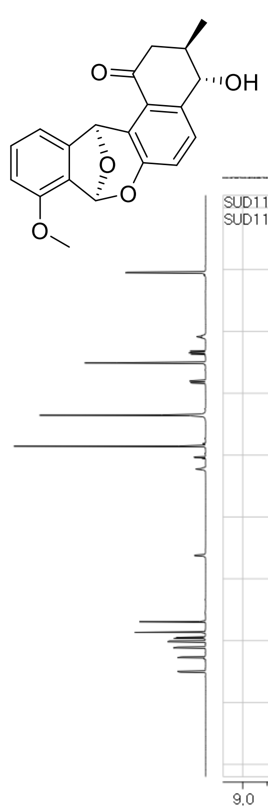

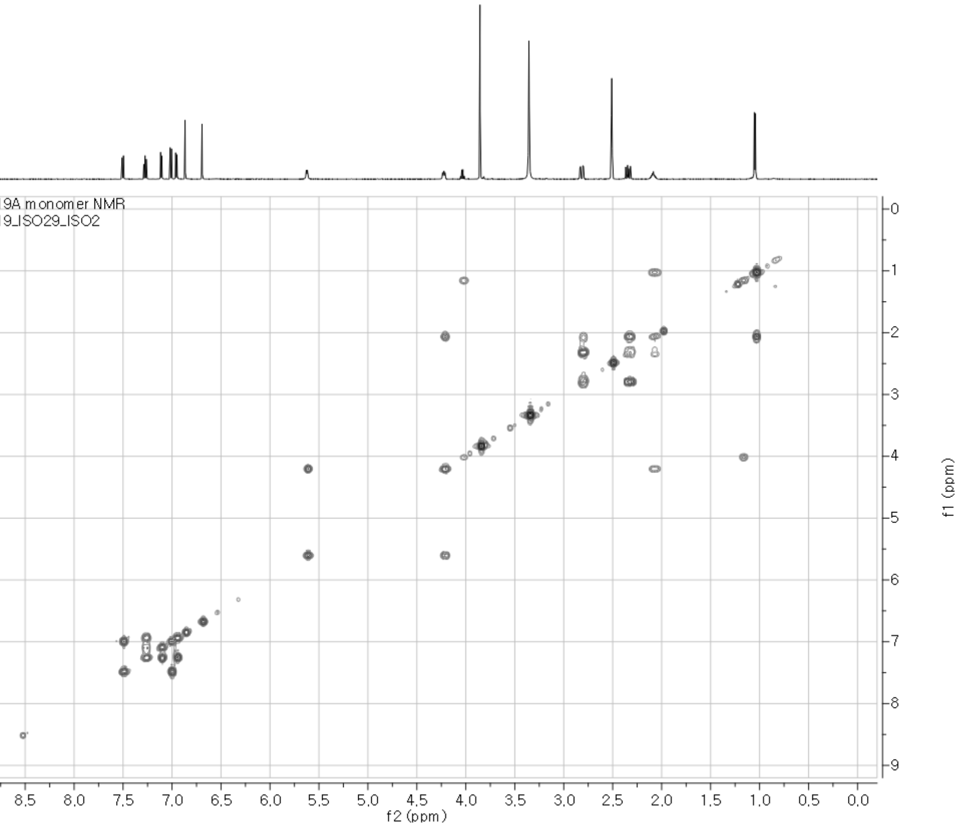

Figure S4. HSQC spectrum (600 MHz) of donghaecyclinone A (**1**) in DMSO–d_6.

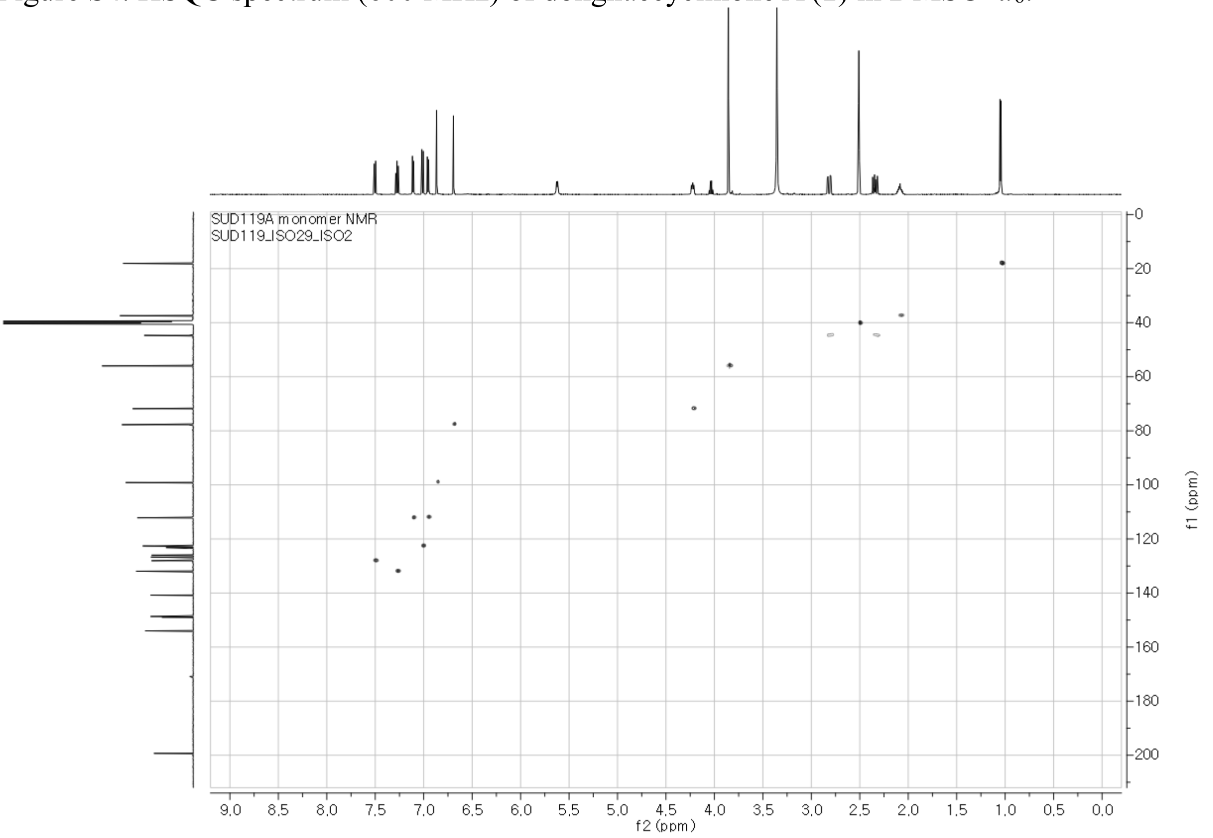

Figure S5. HMBC spectrum (600 MHz) of donghaecyclinone A (**1**) in DMSO–d_6.

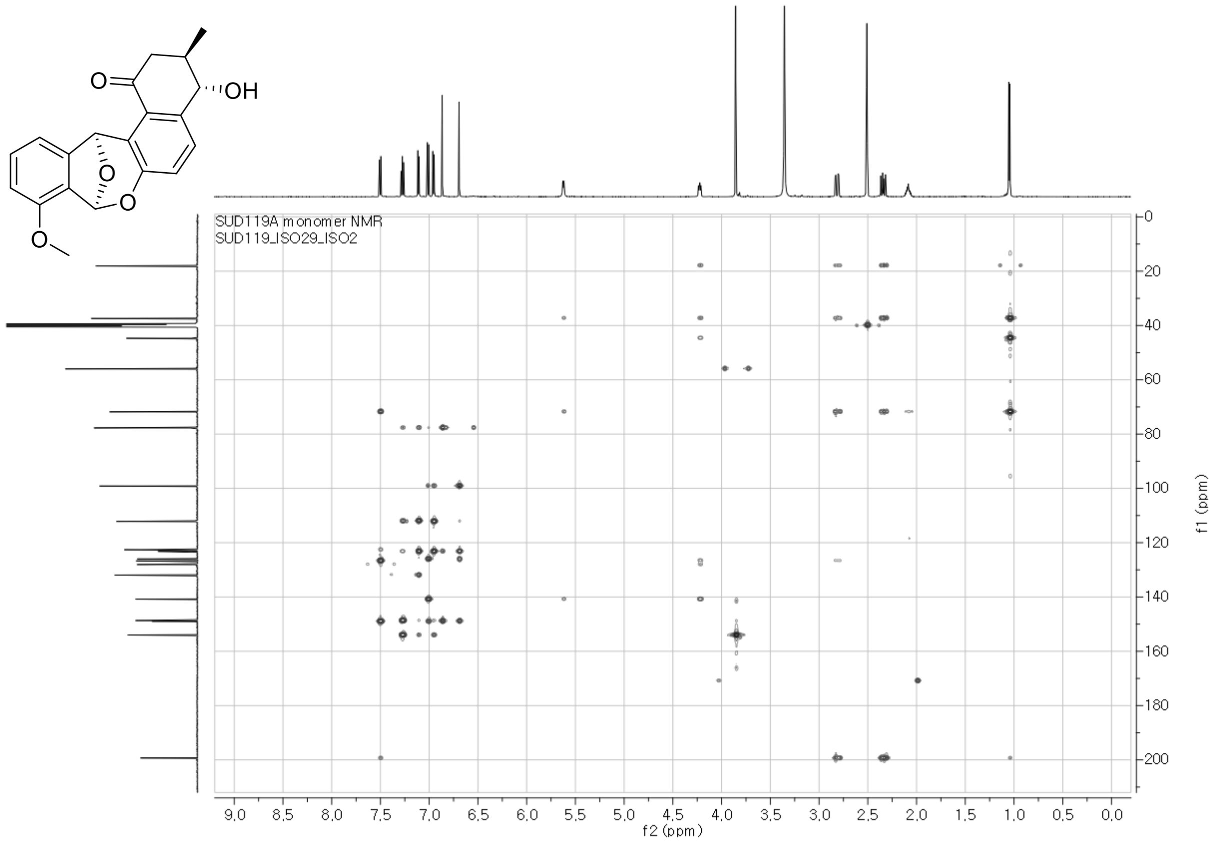

Figure S6. ROESY spectrum (600 MHz) of donghaecyclinone A (**1**) in DMSO–d_6.

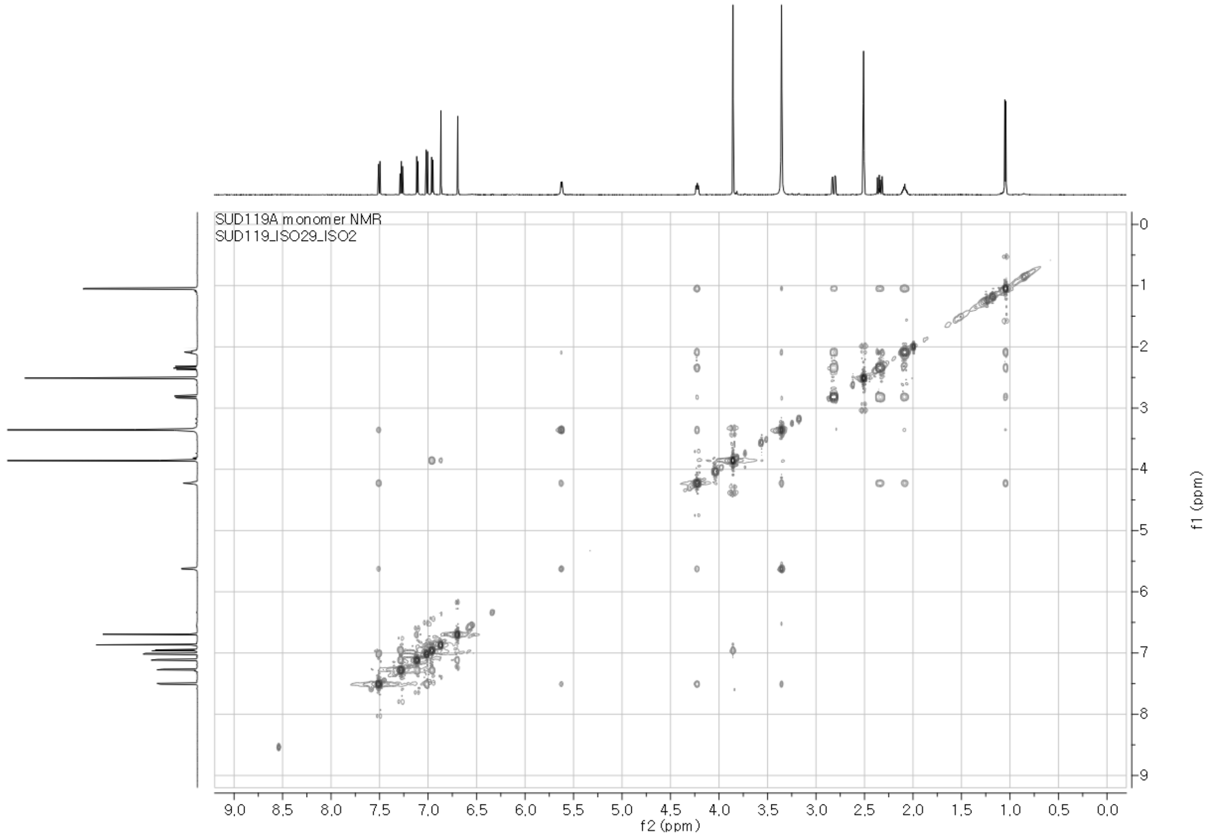

Figure S7. IR spectrum of donghaecyclinone A (**1**).

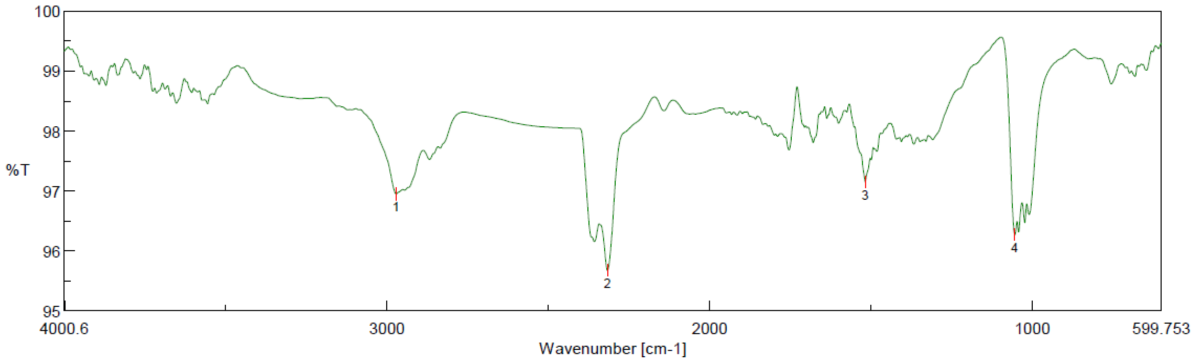

Figure S8. ^{1}H NMR spectrum (800 MHz) of *S*-MTPA ester (**1a**) of **1** in DMSO–d_6.

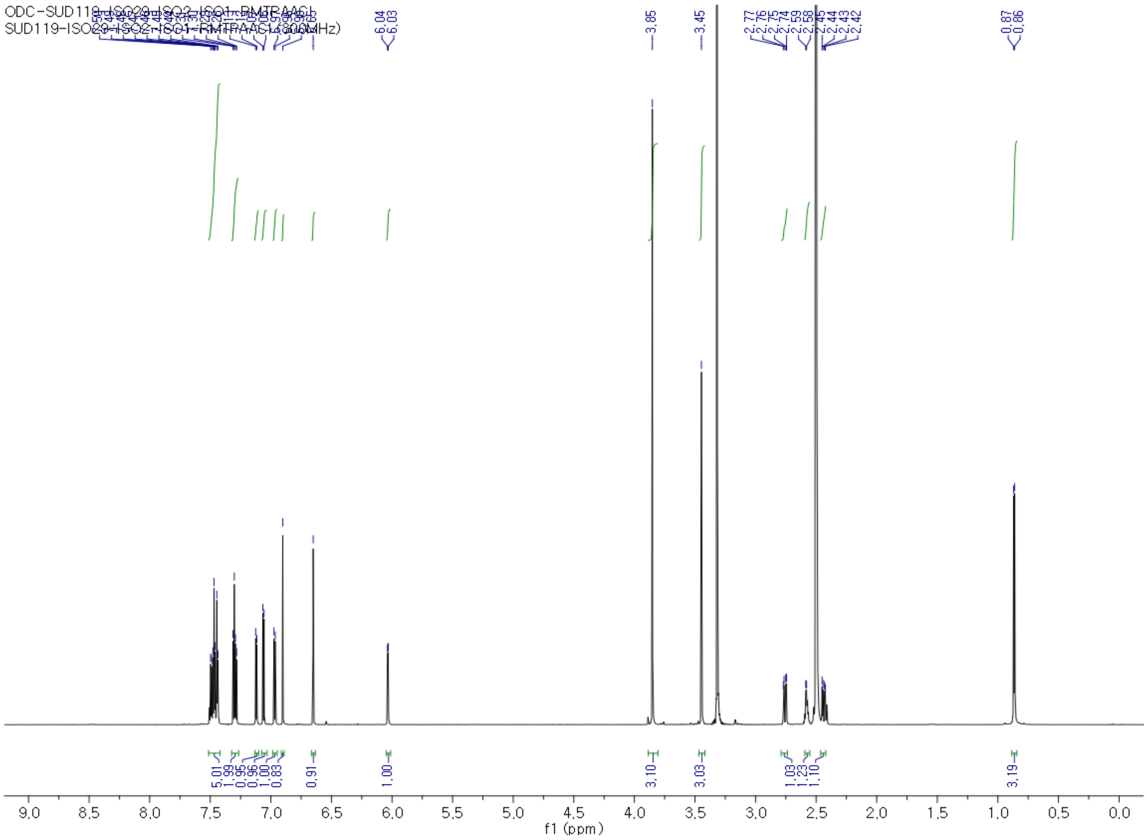

Figure S9. COSY spectrum (800 MHz) of *S*-MTPA ester (**1a**) of **1** in DMSO–d_6.

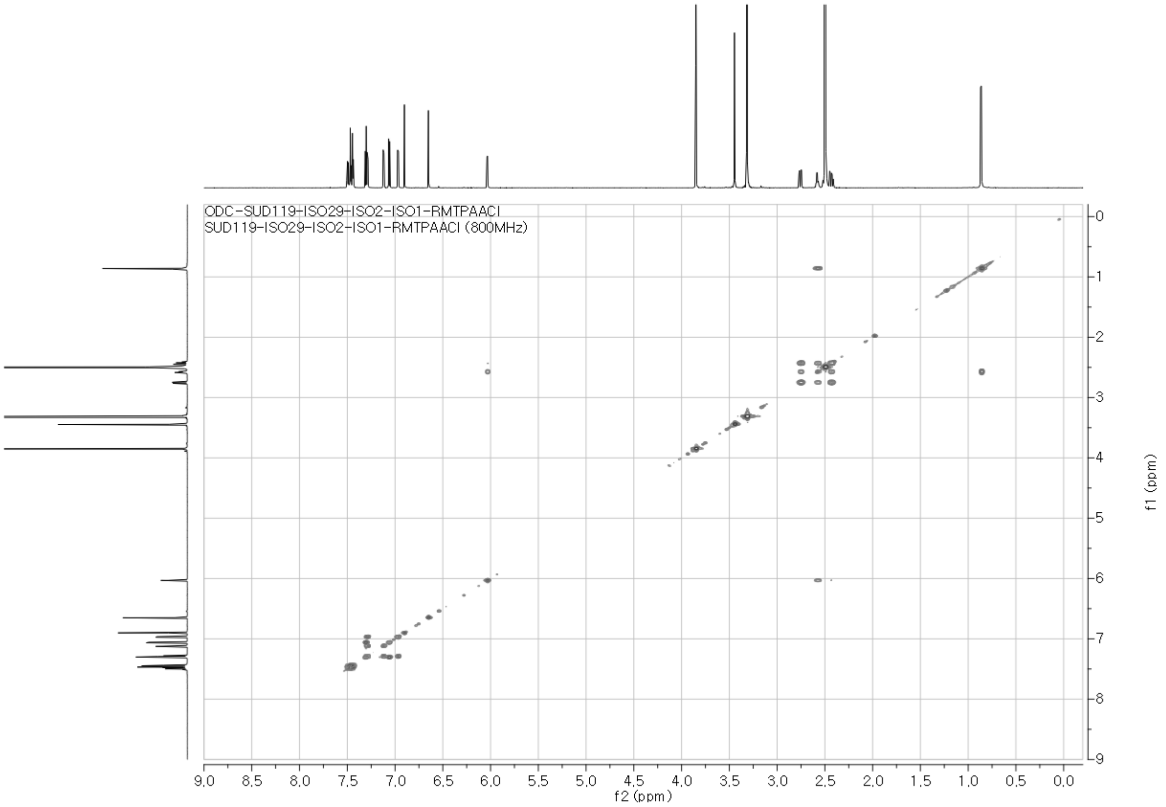

Figure S10. ^{1}H NMR spectrum (800 MHz) of *R*-MTPA ester (**1b**) of **1** in DMSO–d_6.

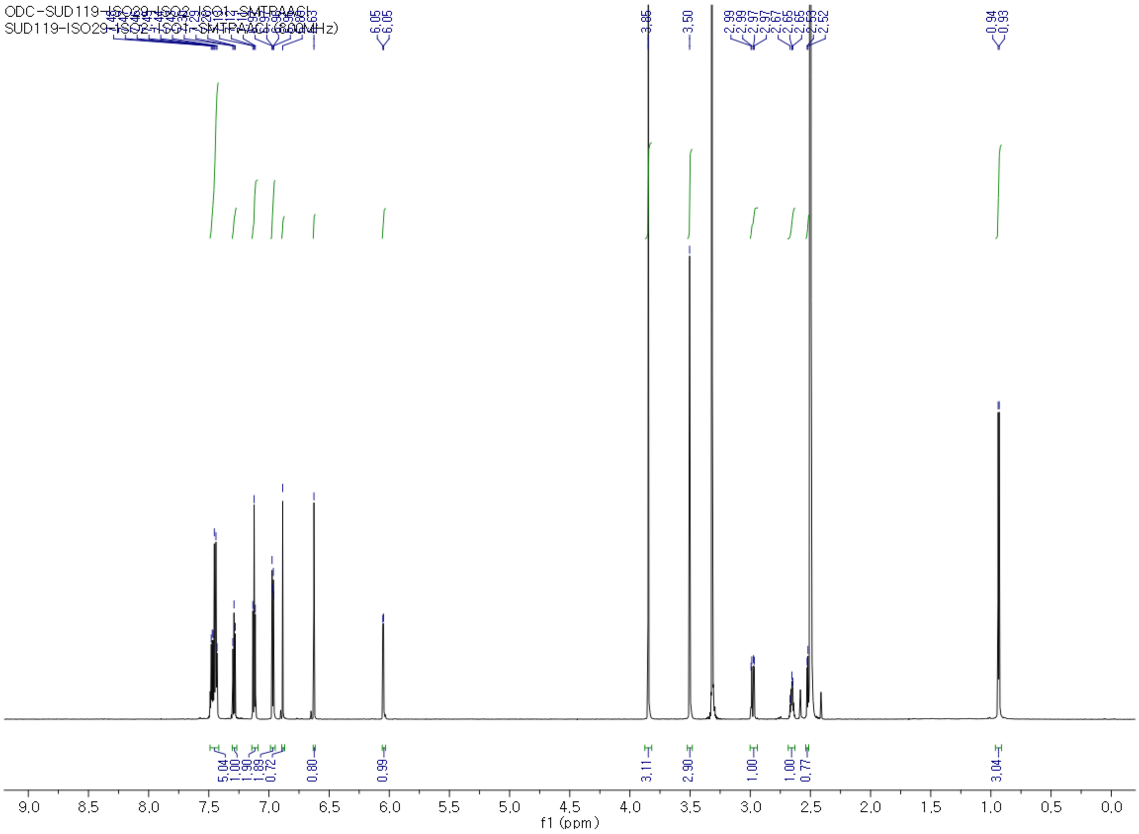

Figure S11. COSY spectrum (800 MHz) of *R*-MTPA ester (**1b**) of **1** in DMSO–d_6.

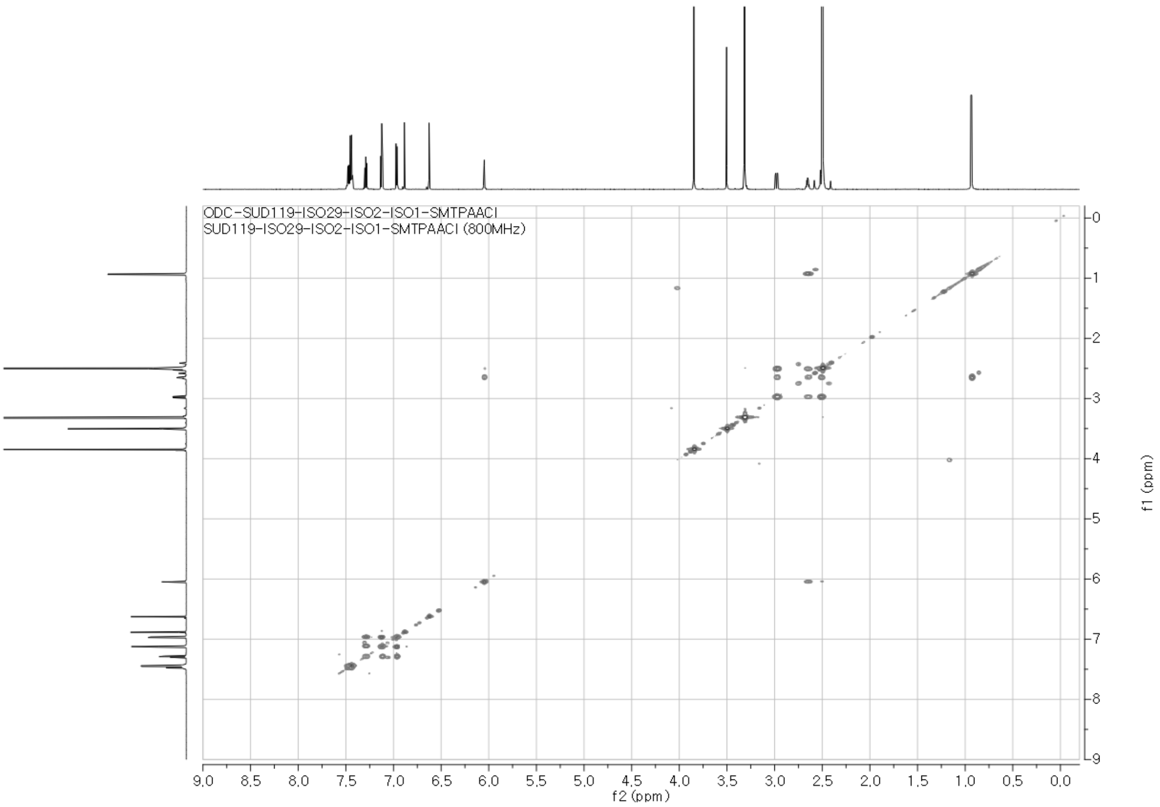

Figure S12. ^{1}H NMR spectrum (600 MHz) of donghaecyclinone B (**2**) in acetone–d_6.

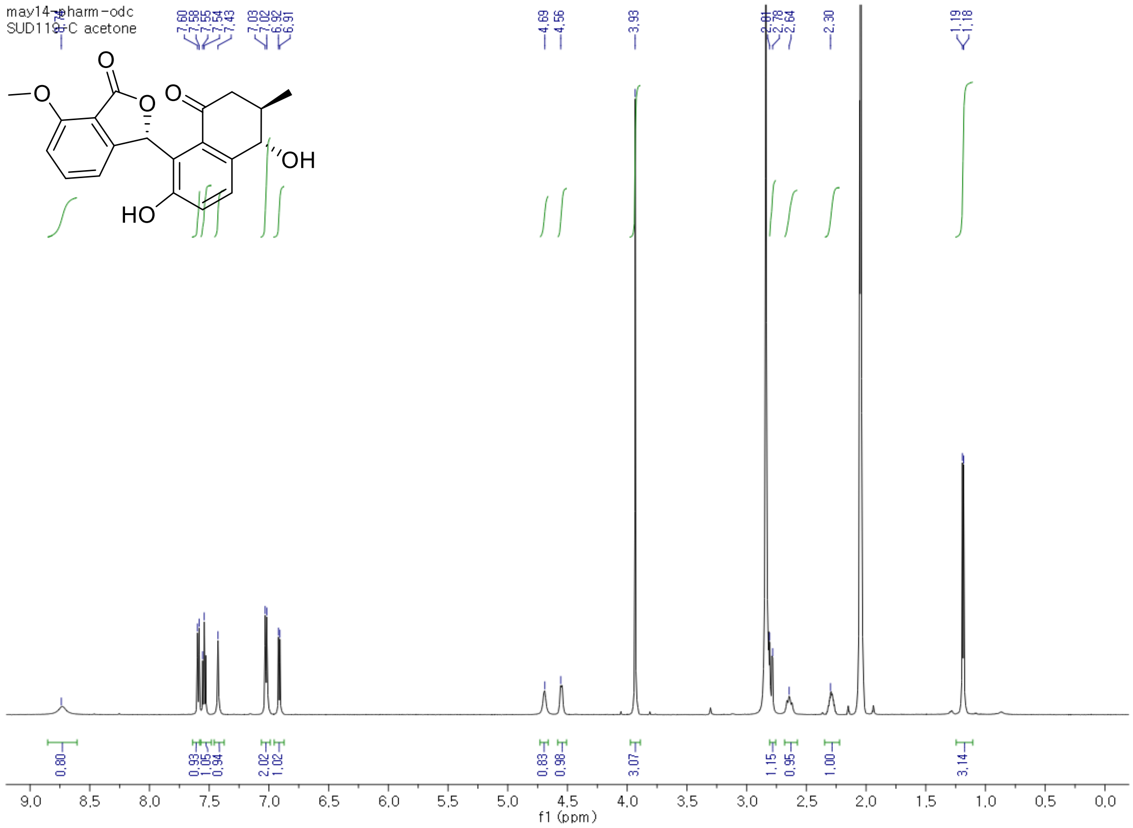

Figure S13. ^{13}C NMR spectrum (150 MHz) of donghaecyclinone B (**2**) in acetone–d_6.

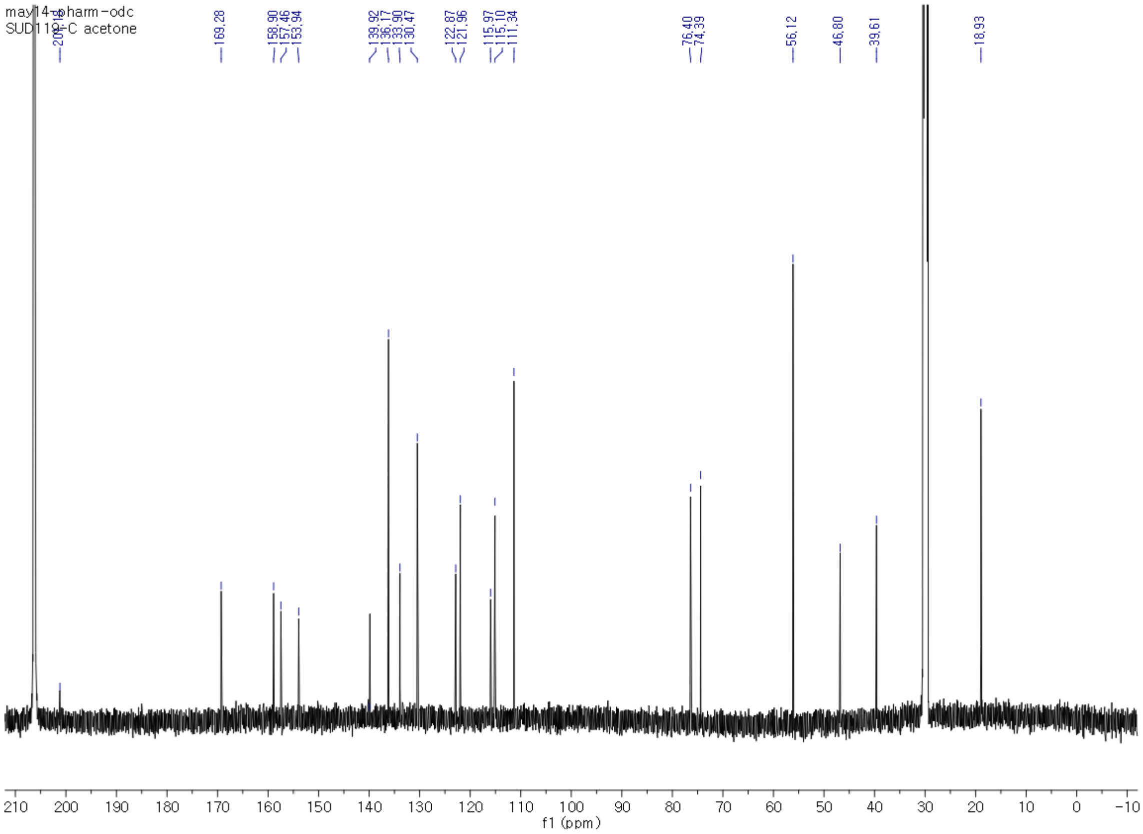

Figure S14. COSY spectrum (600 MHz) of donghaecyclinone B (**2**) in acetone–d_6.

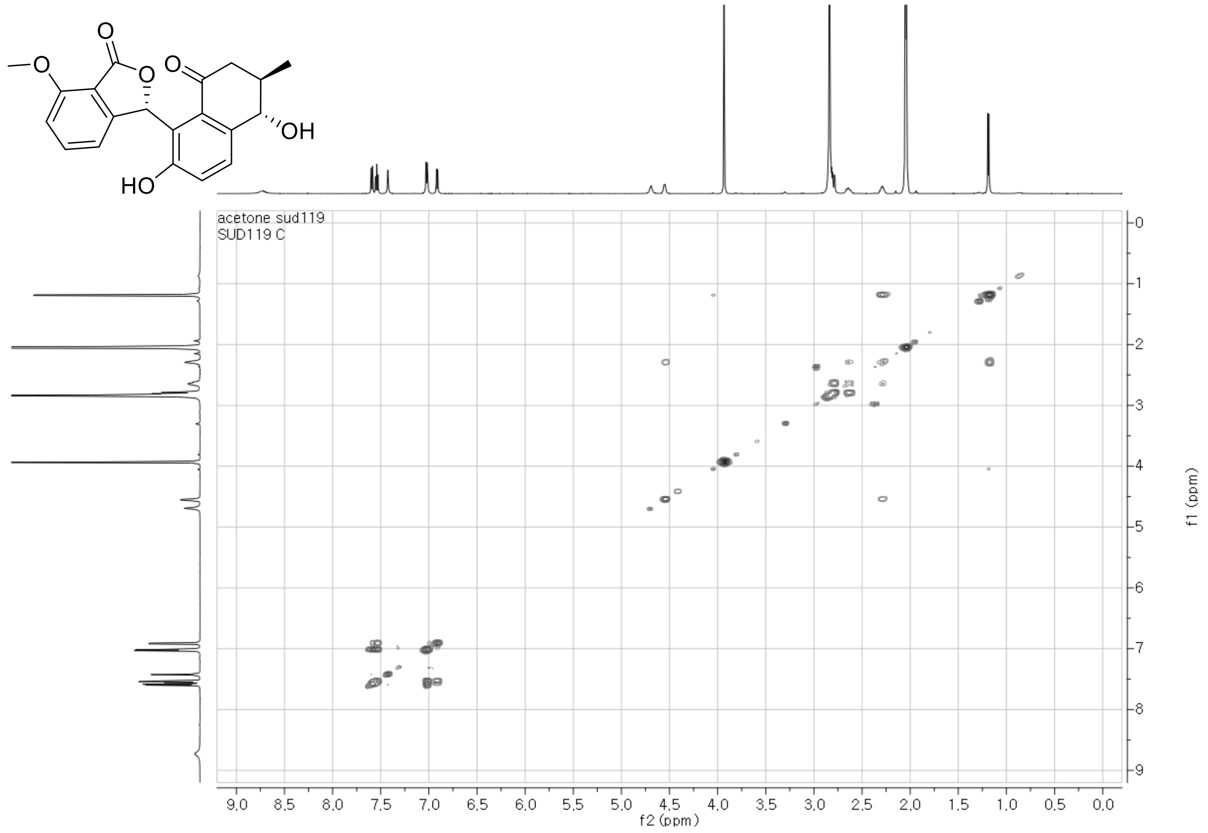

Figure S15. HSQC spectrum (600 MHz) of donghaecyclinone B (**2**) in acetone–d_6.

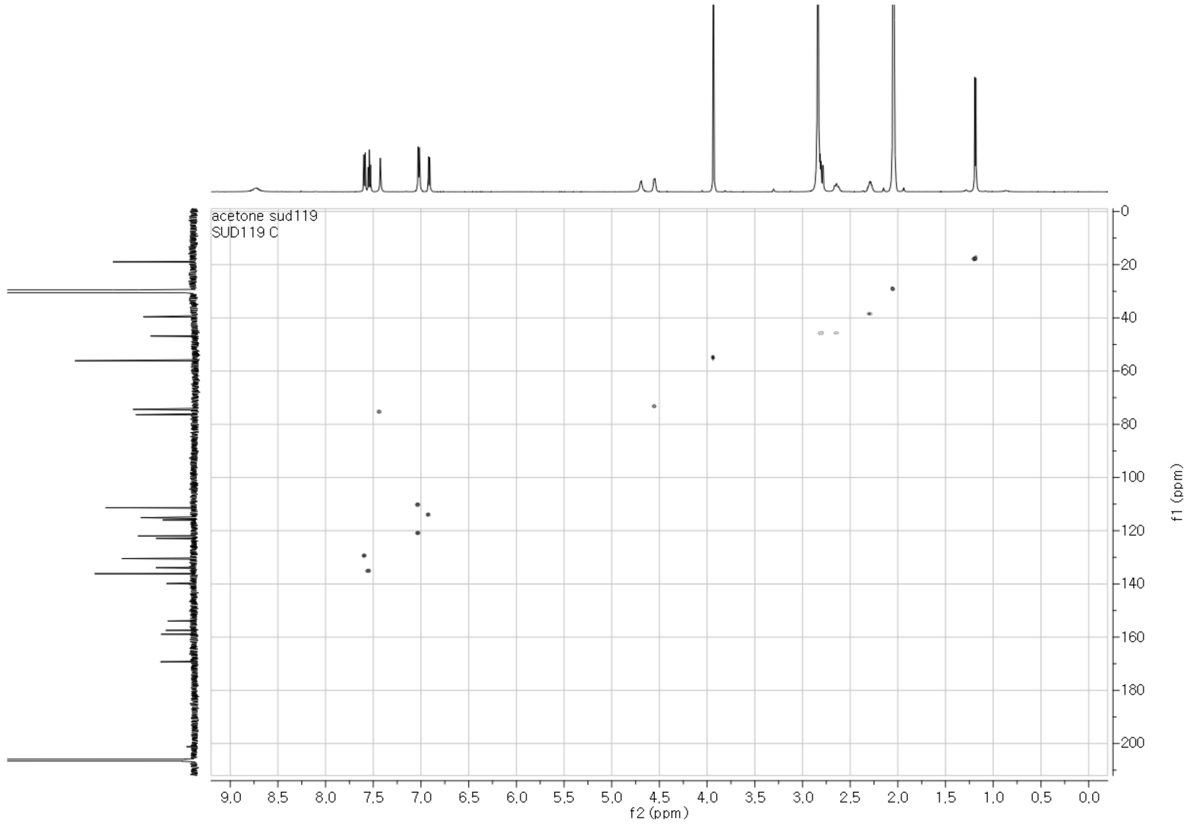

Figure S17. HMBC spectrum (600 MHz) of donghaecyclinone B (**2**) in acetone–d_6.

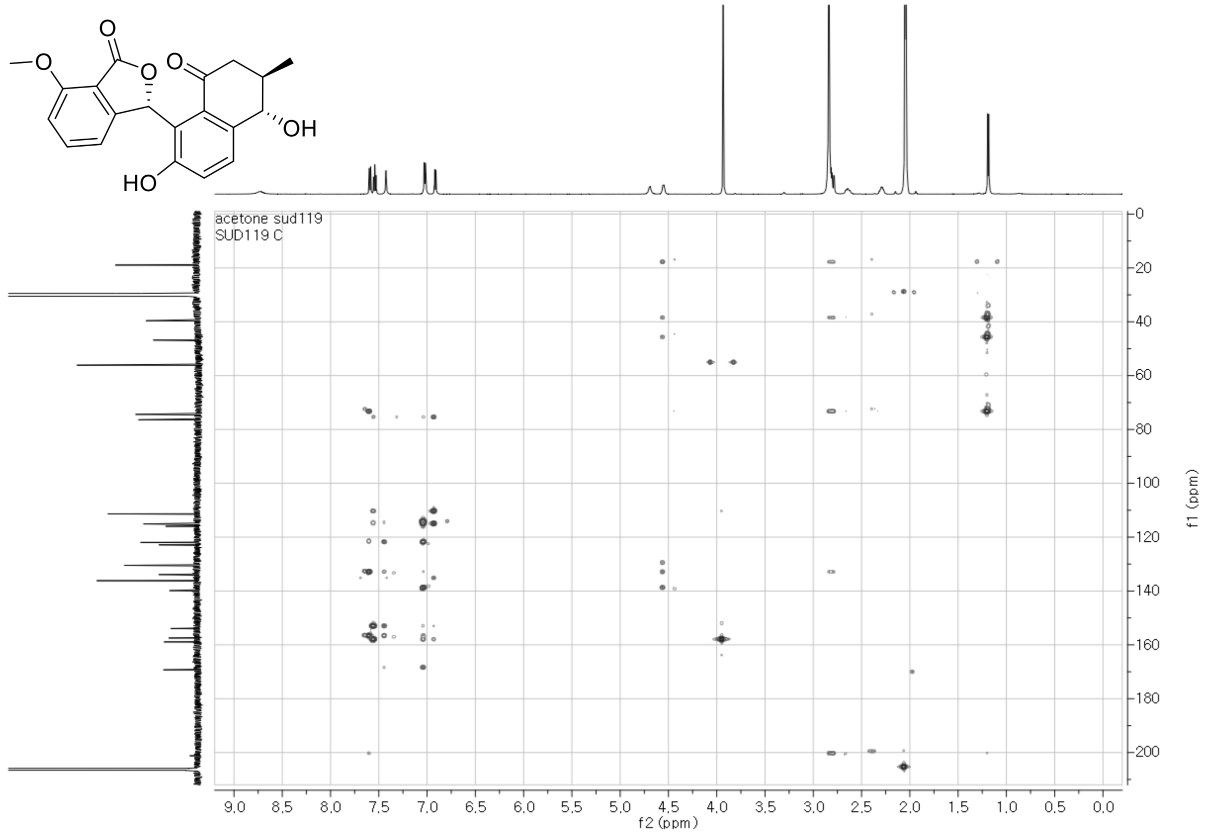

Figure S18. ROESY spectrum (600 MHz) of donghaecyclinone B (**2**) in acetone–d_6.

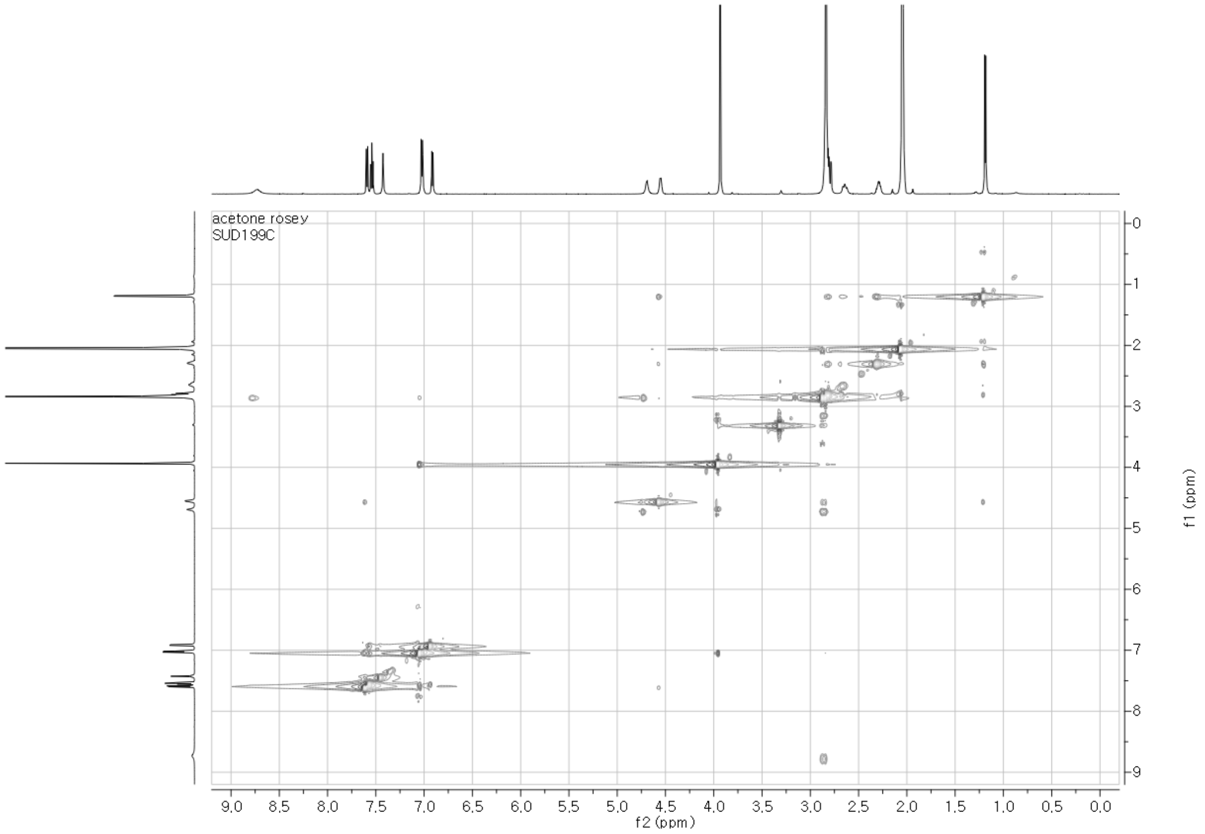

Figure S19. IR spectrum of donghaecyclinone B (**2**).

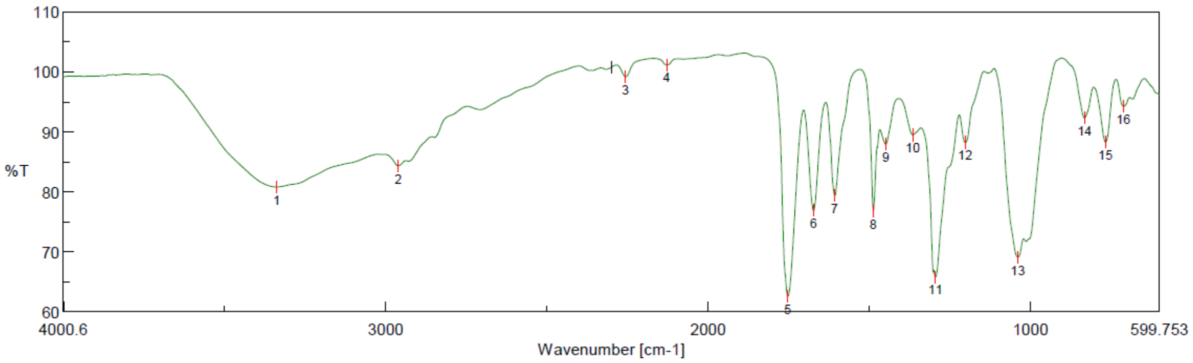

Figure S20. ^{1}H NMR spectrum (600 MHz) of donghaecyclinone C (**3**) in acetone–d_6.

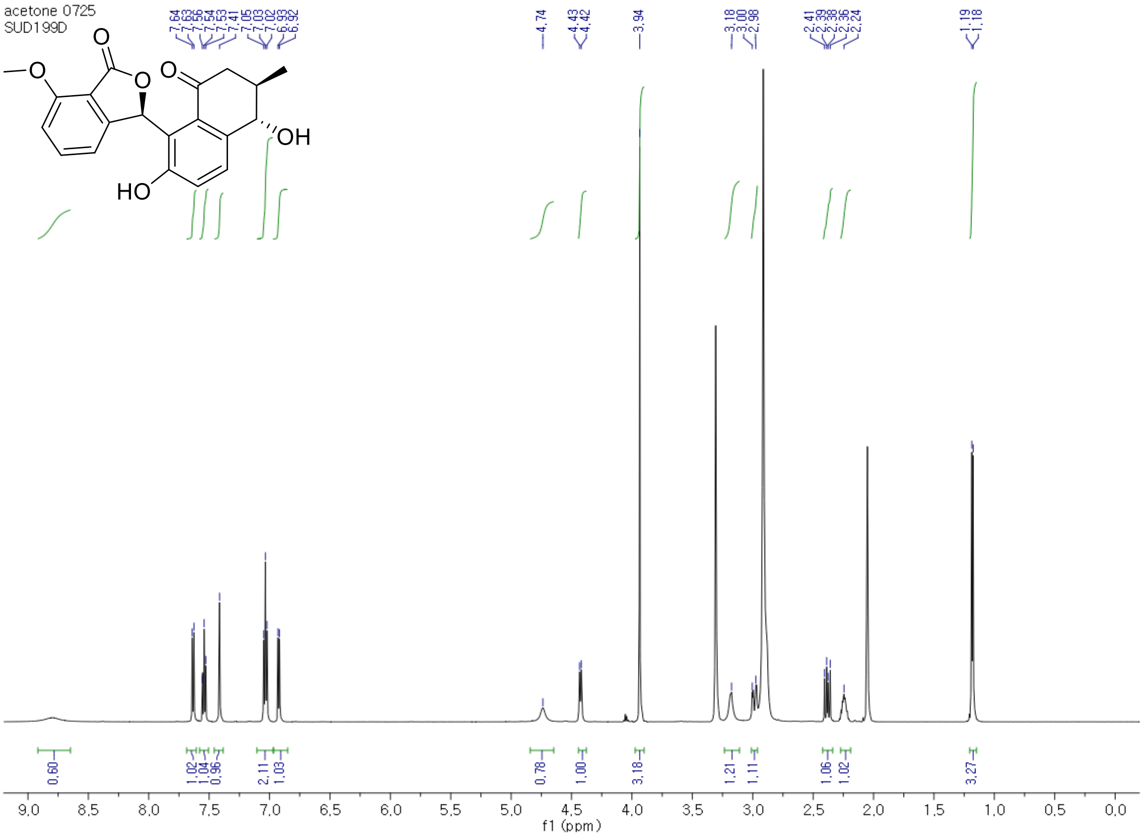

Figure S21. ^{13}C NMR spectrum (150 MHz) of donghaecyclinone C (**3**) in acetone–d_6.

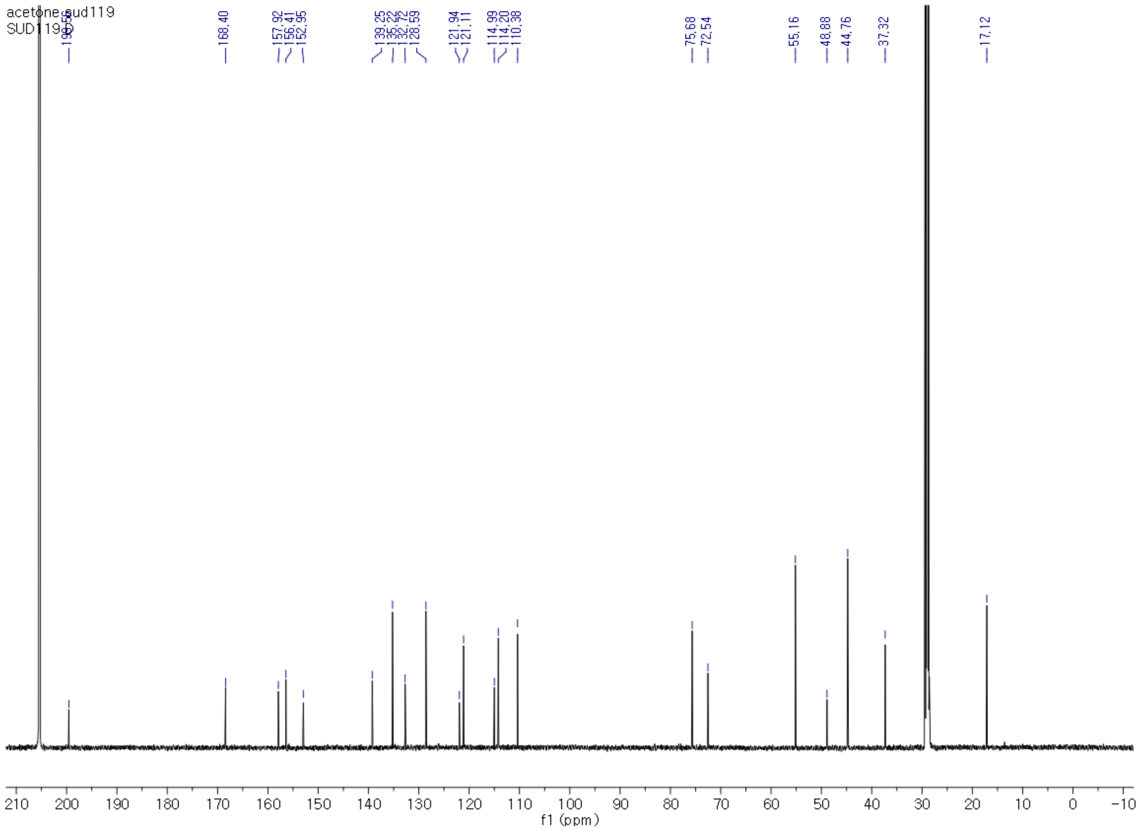

Figure S22. COSY spectrum (600 MHz) of donghaecyclinone C (**3**) in acetone–d_6.

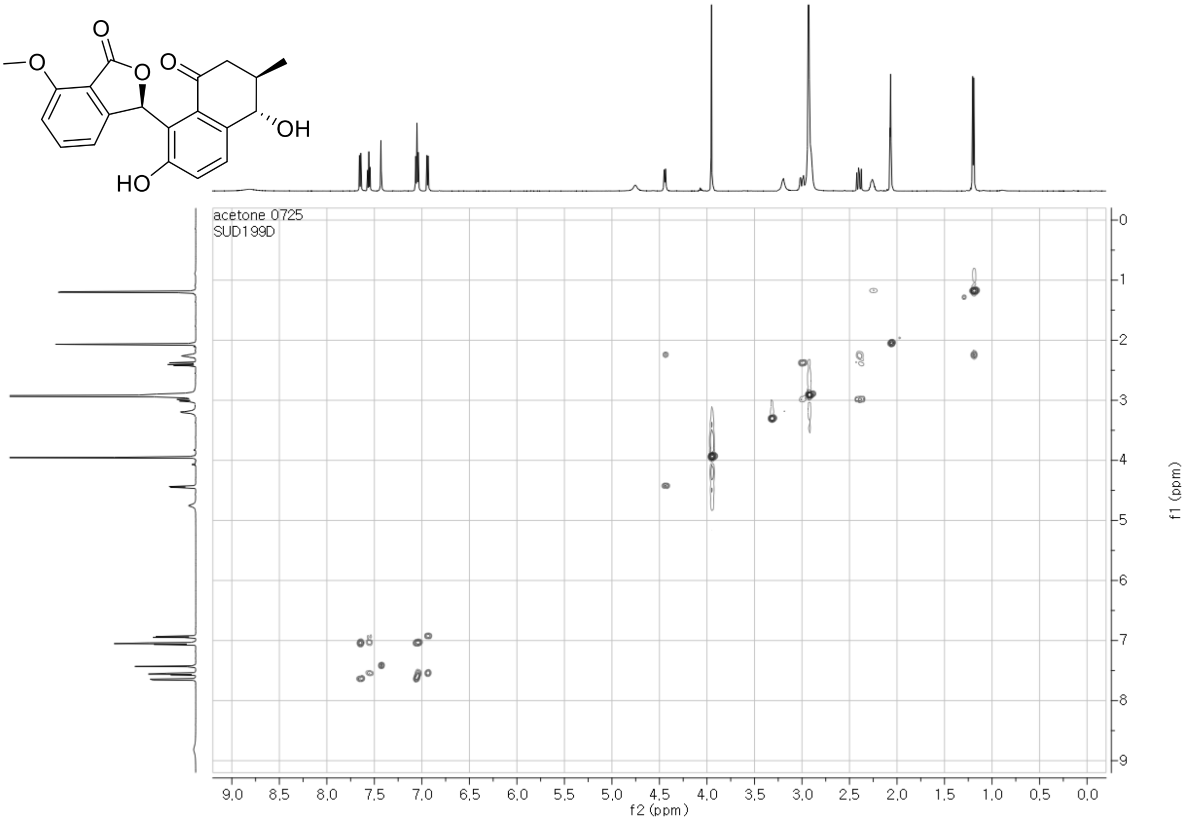

Figure S23. HSQC spectrum (600 MHz) of donghaecyclinone C (**3**) in acetone–d_6.

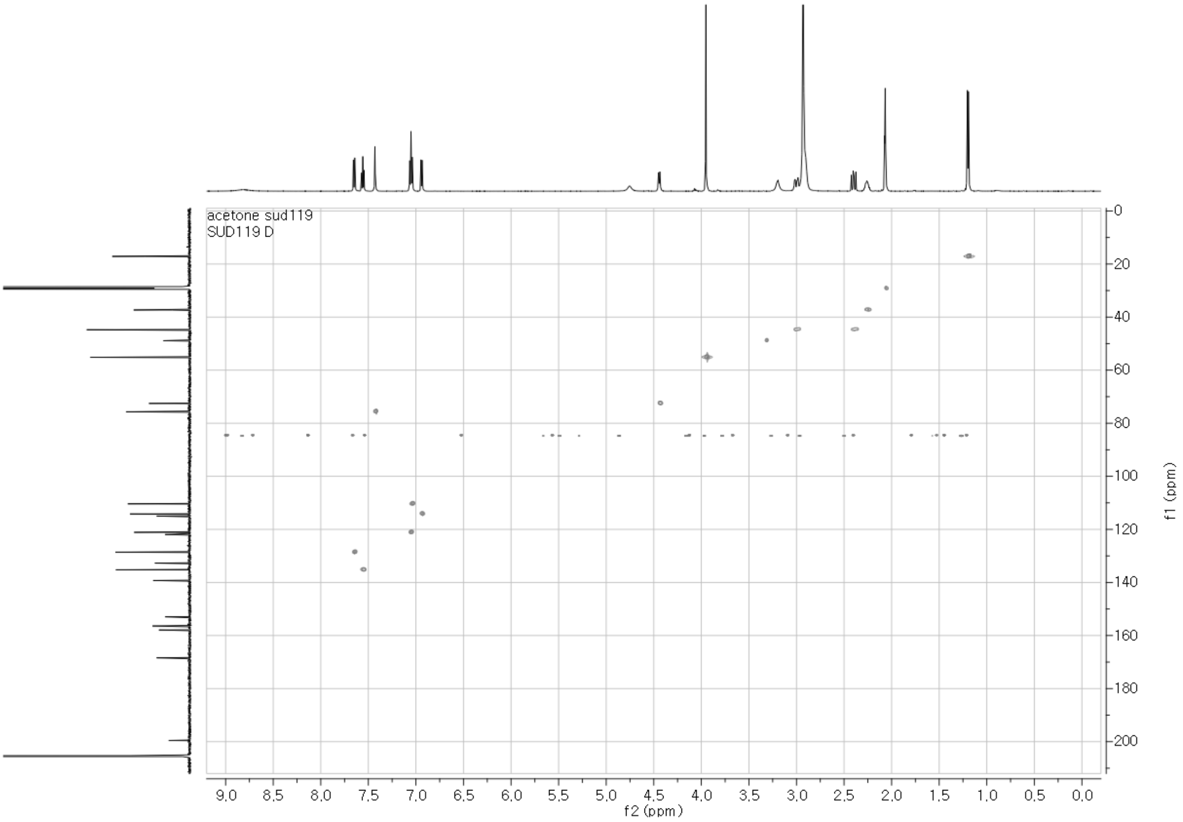

Figure S24. HMBC spectrum (600 MHz) of donghaecyclinone C (**3**) in acetone–d_6.

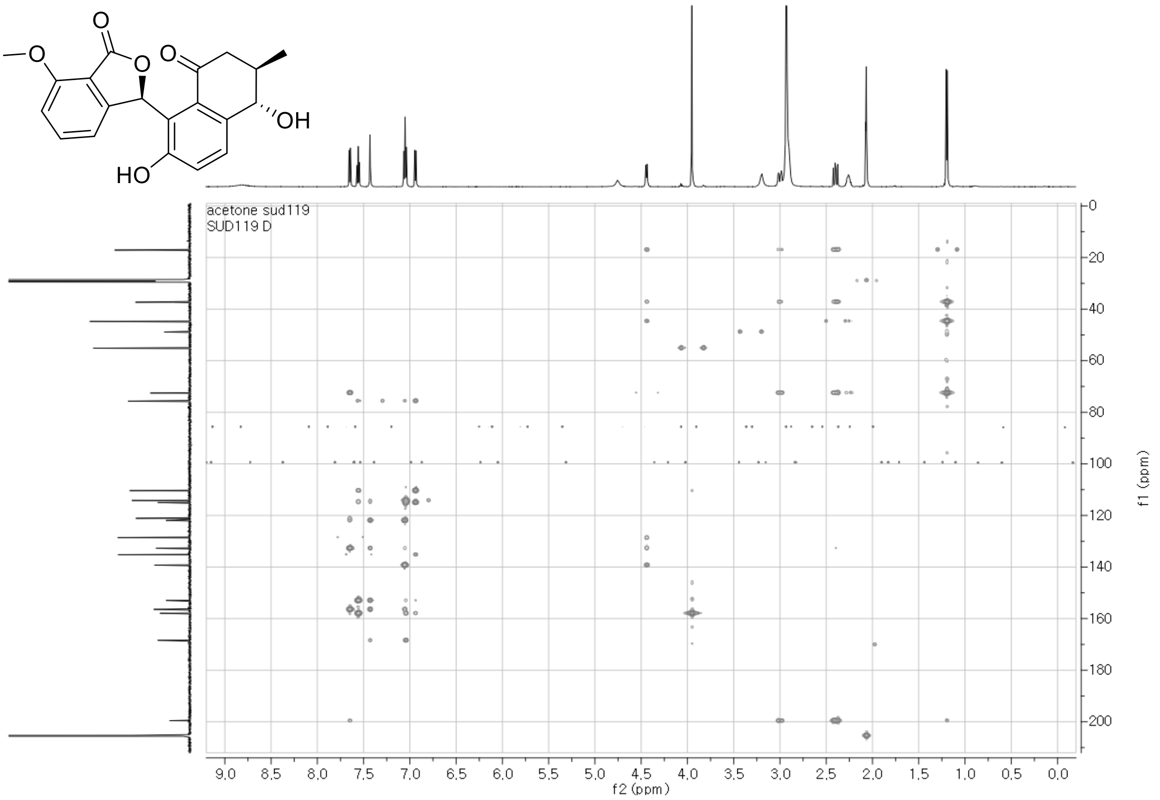

Figure S25. ROESY spectrum (600 MHz) of donghaecyclinone C (**3**) in acetone–d_6.

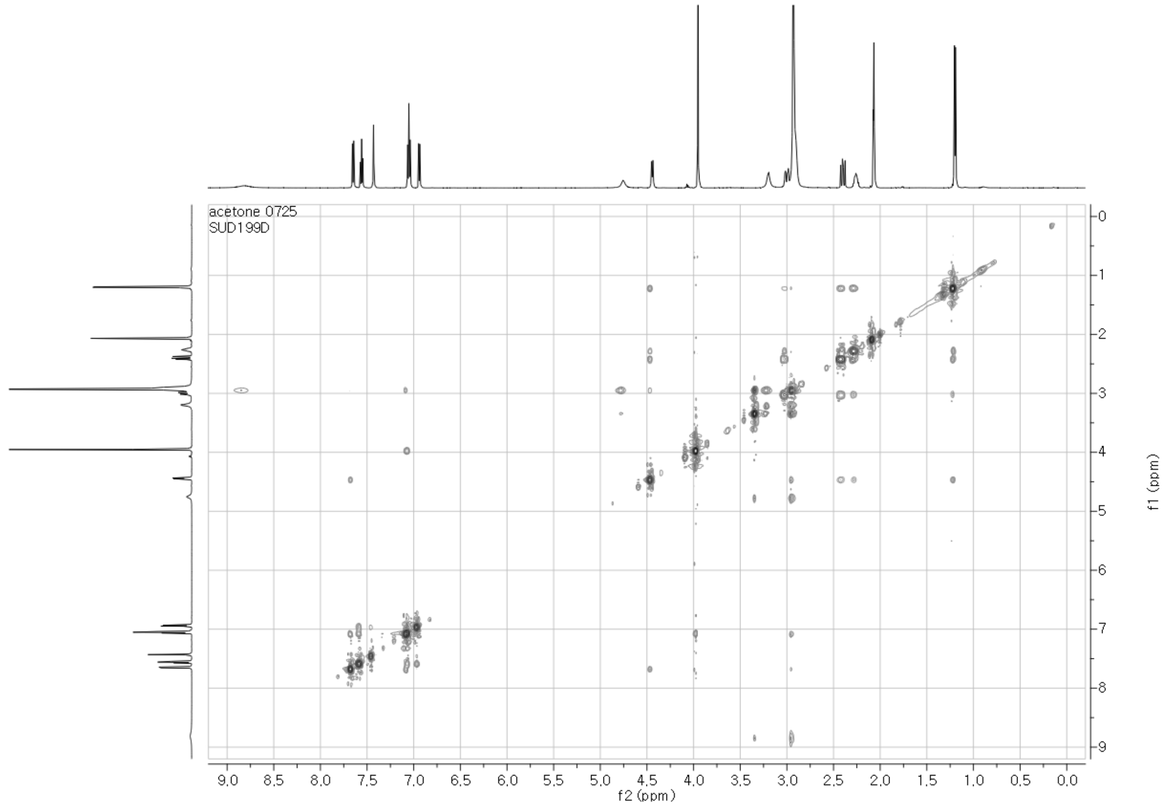

Figure S26. IR spectrum of donghaecyclinone C (**3**).

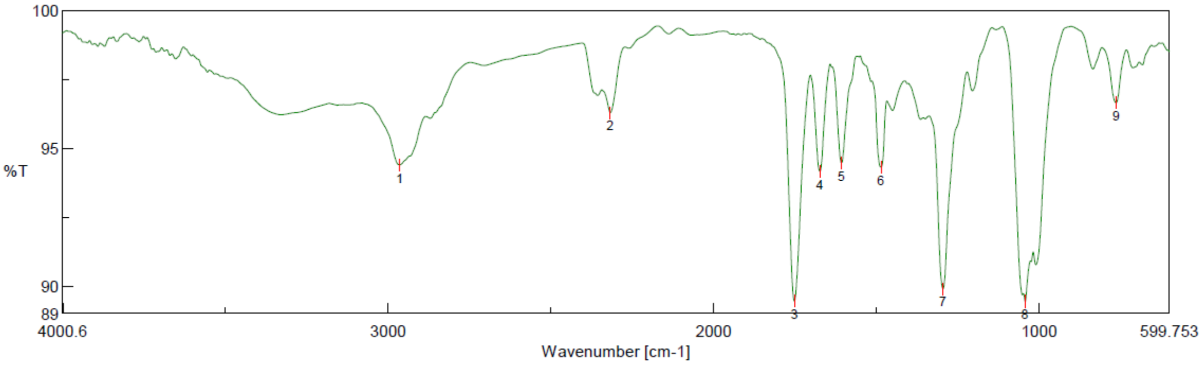

Figure S27. Key ROESY correlations of donghaecyclinone B (**2**).

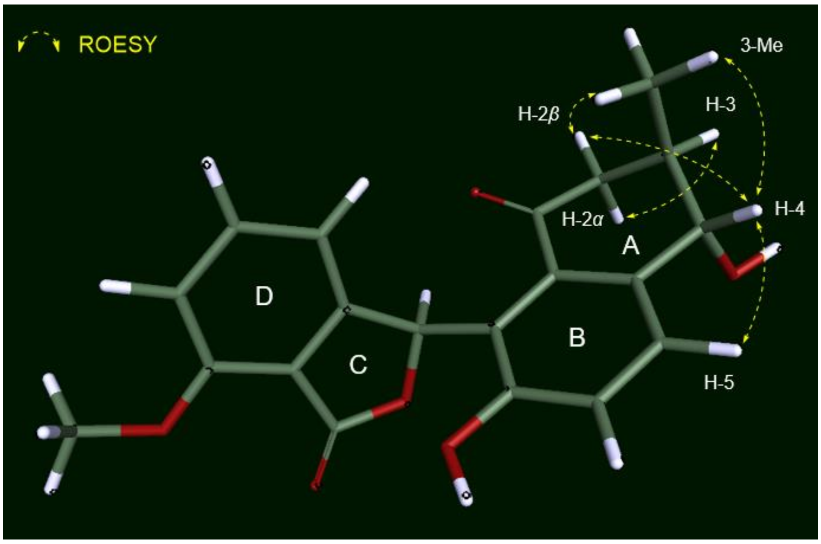

Figure S28. Key ROESY correlations of donghaecyclinone C (**3**).

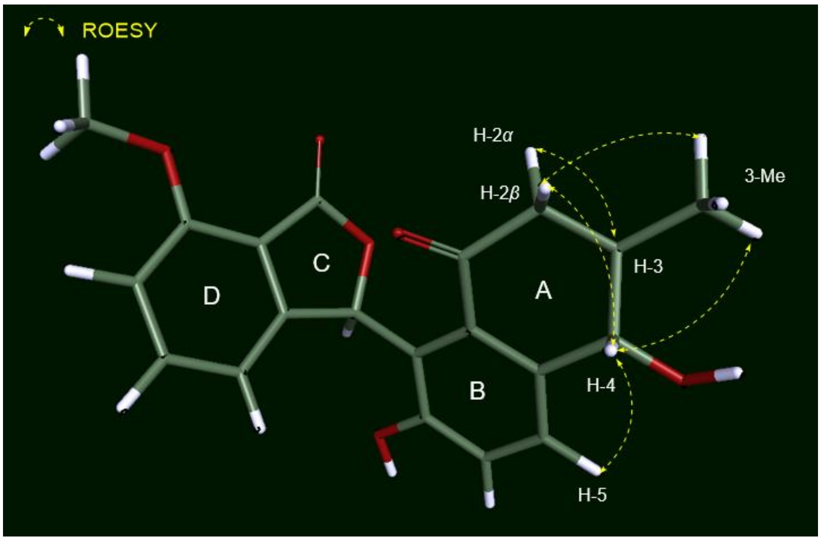

Figure S29. LC/MS chromatograms of the extract of the bacterial strain *Streptomyces* p. SUD119 and donghaecyclinones A-C (**1**-**3**).

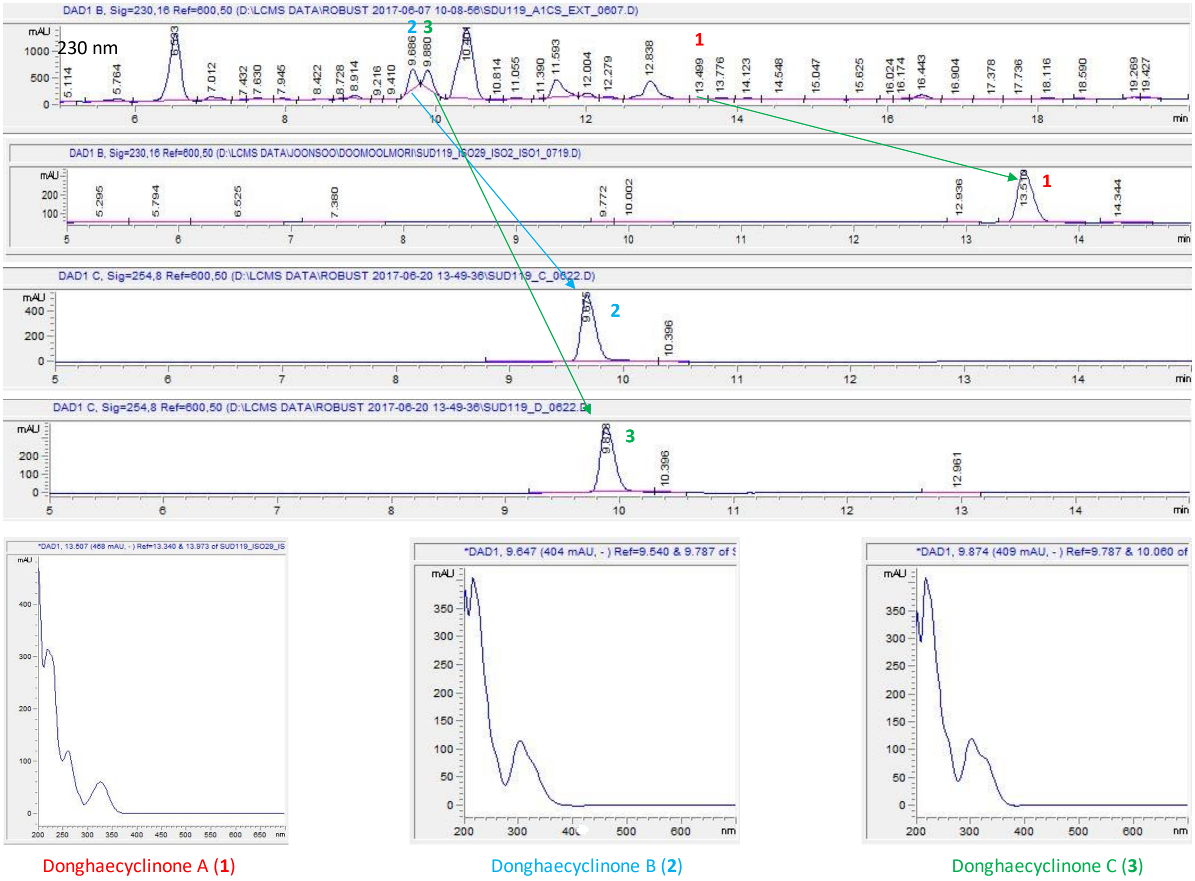

Figure S30. Cytotoxicity result of donghaecyclinones A-C (**1**-**3**) against five cancer cell lines.

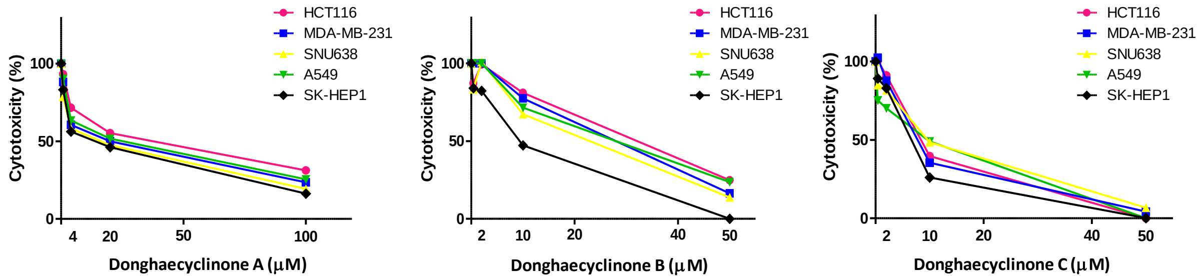

Table S1. The major conformers of diastereomers **1c**–**1d** on donghaecyclinone A (**1**) identified by conformational searches in MMFF94 force field using MacroModel.

Conformers[a]	Boltzmann population (%)[b]
Diastereomer **1c**_1	85.817
Diastereomer **1c**_2	14.183
Diastereomer **1d**_1	84.414
Diastereomer **1d**_2	13.385
Diastereomer **1d**_3	2.201

[a]See Computational Details regarding the procedures for conformational search processes.
[b]Calculated populations based on the relative MMFF94 potential energy of each conformer.

Table S2. The major conformers of diastereomers **2a/3b** identified by conformational searches in MMFF94 force field using MacroModel.

Conformers[a]	Boltzmann population (%)[b]
Diastereomer **2a/3b**_1	25.811984
Diastereomer **2a/3b**_2	25.644898
Diastereomer **2a/3b**_3	12.316803
Diastereomer **2a/3b**_4	11.402585
Diastereomer **2a/3b**_5	10.142626
Diastereomer **2a/3b**_6	9.613082
Diastereomer **2a/3b**_7	1.126579
Diastereomer **2a/3b**_8	0.921017
Diastereomer **2a/3b**_9	0.761389
Diastereomer **2a/3b**_10	0.624365
Diastereomer **2a/3b**_11	0.58827
Diastereomer **2a/3b**_12	0.542915
Diastereomer **2a/3b**_13	0.503487

[a]See Computational Details regarding the procedures for conformational search processes.
[b]Calculated populations based on the relative MMFF94 potential energy of each conformer.

Table S3. The major conformers of diastereomers **2b/3a** identified by conformational searches in MMFF94 force field using MacroModel.

Conformers[a]	Boltzmann population (%)[b]
Diastereomer **2b/3a**_1	26.310415
Diastereomer **2b/3a**_2	25.447397
Diastereomer **2b/3a**_3	12.349908
Diastereomer **2b/3a**_4	11.003817
Diastereomer **2b/3a**_5	9.558681
Diastereomer **2b/3a**_6	8.84461
Diastereomer **2b/3a**_7	1.070525
Diastereomer **2b/3a**_8	0.883364
Diastereomer **2b/3a**_9	0.873651
Diastereomer **2b/3a**_10	0.831517
Diastereomer **2b/3a**_11	0.824524
Diastereomer **2b/3a**_12	0.754517
Diastereomer **2b/3a**_13	0.652495
Diastereomer **2b/3a**_14	0.59458

[a]See Computational Details regarding the procedures for conformational search processes.
[b]Calculated populations based on the relative MMFF94 potential energy of each conformer.

Table S4. Experimental (Exp.) and calculated (Cal.) chemical shift values (CS, δ) of diastereomers **1c**–**1d** on donghaecyclinone A (**1**).

	Exp. CS of donghaecyclinone A (**1**)	Cal. CS of **1c**	Cal. CS of **1d**
C-1	111.7	113.92	114.27
C-2	148.3	151.55	151.81
C-3	131.5	132.12	131.93
C-4	111.5	110.41	110.30
C-5	153.6	152.27	152.22
C-6	122.8	126.55	126.38
C-11	55.6	57.24	57.41
C-15	77.3	82.22	82.32
C-16	98.7	102.97	102.91
C-17	125.9	130.91	130.86
C-20	148.5	152.51	152.40
C-23	126.3	129.09	129.39
C-24	140.4	133.85	134.12
C-25	127.6	132.67	132.59
C-26	122.2	123.11	122.95
C-29	198.9	198.54	200.12
C-30	44.3	45.8	47.07
C-31	37.0	44.55	46.57
C-34	71.4	79.36	79.23
C-35	17.6	24.85	23.89
H-7	7.10	6.89	6.97
H-8	7.27	6.72	6.74
H-9	6.94	5.96	5.91
H-12	3.85	3.83	3.82
H-13	3.85	3.37	3.38
H-14	3.85	3.36	3.37
H-19	6.68	6.32	6.14
H-22	6.86	6.09	6.07
H-27	7.00	6.46	6.40
H-28	7.49	6.84	6.85
H-32	2.32	2.04	1.89
H-33	2.81	3.11	3.42
H-36	2.08	1.71	1.75
H-37	4.22	4.38	4.31
H-40	1.04	1.01	0.98
H-41	1.04	0.89	0.81
H-42	1.04	0.83	0.62

Table S5. Experimental (Exp.) and calculated (Cal.) chemical shift values (CS, δ) of diastereomers **2a/3b**–**2b/3a** on donghaecyclinones B and C (**2** and **3**).

	Exp. CS of **2**	Exp. CS of **3**	Cal. CS of **2a/3b**	Cal. CS of **2b/3a**
C-1	201.2	200.4	196.12	195.14
C-2	169.3	169.3	159.27	159.44
C-3	158.9	158.8	152.17	152.15
C-4	157.5	157.3	152.08	151.91
C-5	153.9	153.8	141.95	142.09
C-6	139.9	140.1	132.42	132.5
C-7	136.2	136.1	130.36	130.48
C-8	133.9	132.7	128.64	128.7
C-9	130.5	129.4	127.69	127.91
C-10	122.9	122.8	121.3	121.01
C-11	122.0	121.9	120.49	120.99
C-12	116.0	115.8	114.31	114.71
C-13	115.1	115.0	112.63	112.51
C-14	111.3	111.2	111.84	111.82
C-15	76.4	76.5	79.96	79.58
C-16	74.4	73.4	73.79	74.08
C-17	56.1	56.0	56.22	56.16
C-18	46.8	45.6	42.26	40.96
C-19	39.6	38.2	39.05	37.19
C-20	18.9	18.0	18.58	19.18
H-1	7.59	7.63	7.93	7.73
H-2	7.54	7.54	7.50	7.54
H-3	7.43	7.41	7.44	7.39
H-4	7.04	7.04	7.21	7.19
H-5	7.03	7.05	6.91	6.94
H-6	6.92	6.93	6.86	6.91
H-7	4.55	4.43	4.72	4.69
H-8	3.93	3.94	3.98	3.96
H-9	3.93	3.94	3.87	3.76
H-10	3.93	3.94	3.57	3.72
H-11	2.81	2.99	3.48	3.39
H-12	2.64	2.38	2.34	2.33
H-13	2.29	2.24	2.21	2.2
H-14	1.20	1.18	1.11	1.0
H-15	1.20	1.18	1.1	0.97
H-16	1.20	1.18	0.9	0.85

Cartesian coordinates of the conformers shown in Table S4.

Diastereomer **1c**_1

atomic	coordinates	atom	charge	isotop	
8.04904438	7.57134054	11.6482947	c	6	0
5.76741775	8.81679779	11.2297086	c	6	0
10.0581498	9.02259018	12.5808613	c	6	0
9.81959807	11.6243324	13.0336871	c	6	0
7.51722018	12.8687763	12.5444329	c	6	0
5.50196195	11.407105	11.6609928	c	6	0
8.24820252	5.5505781	11.2779516	h	1	0
11.8733578	8.11275037	12.9812124	h	1	0
11.4390155	12.6658527	13.774549	h	1	0
7.08770348	15.3718552	12.8908063	o	8	0
9.05876352	16.9268326	13.7852417	c	6	0
10.6983263	16.9219041	12.4837206	h	1	0
9.69999435	16.3450439	15.6913965	h	1	0
8.28173427	18.8546684	13.8899826	h	1	0
3.23361207	7.95783048	10.1659433	c	6	0
2.82842963	12.0905278	10.89886	c	6	0
3.25337784	8.37675939	7.31217451	c	6	0
1.51450446	9.82495284	11.1742793	o	8	0
2.59844869	6.08063764	10.7388919	h	1	0
2.93422619	10.9257599	6.57118656	c	6	0
2.73602287	12.8419736	8.2732343	o	8	0
1.89991391	13.6190339	11.9459179	h	1	0
3.53612444	6.5010769	5.43310961	c	6	0
3.33504024	7.14974509	2.84497427	c	6	0
2.97242195	9.68174115	2.17649822	c	6	0
2.7913402	11.5631283	4.00713028	c	6	0
2.52162989	13.5476687	3.50025972	h	1	0
2.83701879	10.1899322	0.1758449	h	1	0
4.10280948	3.82646868	6.15680285	c	6	0
4.17832566	1.85238893	4.06188062	c	6	0
2.67869355	2.55325058	1.68445633	c	6	0
3.57795192	0.03342008	4.88561826	h	1	0
6.19737783	1.65315083	3.55751702	h	1	0
3.57138835	5.17440924	0.78503185	c	6	0
-0.1925866	2.41186066	2.07047637	c	6	0
3.17759523	1.19002994	0.1748801	h	1	0
2.38105327	5.78080043	-0.8322196	h	1	0
6.15881659	5.1151929	0.01687074	o	8	0
4.60789386	3.21384234	8.3225251	o	8	0
-1.2153234	2.94251628	0.32733238	h	1	0
-0.8397765	3.6857237	3.59345215	h	1	0
-0.7701846	0.47114256	2.57941173	h	1	0
6.30601616	3.94749441	-1.3964544	h	1	0

Diastereomer **1c**_2

atomic	coordinates	atom	charge	isotop	
8.03488077	7.52076917	11.6051695	c	6	0
5.76546608	8.7860543	11.1801408	c	6	0
10.0688989	8.96640971	12.4917454	c	6	0
9.86606643	11.5800279	12.8908185	c	6	0
7.57525976	12.8431917	12.3940024	c	6	0
5.53460493	11.3879597	11.5588759	c	6	0
8.20516121	5.49054054	11.274627	h	1	0
11.8749664	8.04223493	12.9003825	h	1	0
11.504622	12.6164671	13.5958357	h	1	0
7.18010562	15.3578949	12.6896739	o	8	0
9.17561695	16.9057967	13.5423328	c	6	0
10.8074013	16.8544503	12.2320636	h	1	0
9.82018358	16.3537302	15.4560802	h	1	0
8.42305574	18.844782	13.6129005	h	1	0
3.21229556	7.93588557	10.1568715	c	6	0
2.86145154	12.0870567	10.8100114	c	6	0
3.21647005	8.29792734	7.2956914	c	6	0
1.52427833	9.84325363	11.1410589	o	8	0
2.56028339	6.07737675	10.7711966	h	1	0
2.90968268	10.8376175	6.50489198	c	6	0
2.7509486	12.7881396	8.16936508	o	8	0
1.96086744	13.6470072	11.8346383	h	1	0
3.4778451	6.38521782	5.45607954	c	6	0
3.28039928	6.98377713	2.85297817	c	6	0
2.89805353	9.50102496	2.13653364	c	6	0
2.7373899	11.4226424	3.93178536	c	6	0
2.45553044	13.3955821	3.3874539	h	1	0
2.71750042	9.97094306	0.12800718	h	1	0
3.9926201	3.70878714	6.22506715	c	6	0
3.95532448	1.6762724	4.18670837	c	6	0
2.57154641	2.38993758	1.73913195	c	6	0
3.19427563	-0.062425	5.05199317	h	1	0
5.96146301	1.29446026	3.74586958	h	1	0
3.57108147	4.95854927	0.84367731	c	6	0
-0.3140679	2.35030678	2.01697922	c	6	0
3.10628181	1.00875919	0.26825351	h	1	0
2.50345298	5.56116324	-0.8582213	h	1	0
6.16613434	4.59091208	0.18961917	o	8	0
4.53057379	3.14467658	8.39629987	o	8	0
-1.2533447	2.88211453	0.22805311	h	1	0
-0.9800693	3.66632523	3.49635283	h	1	0
-0.9698877	0.43629612	2.53201421	h	1	0
6.95286009	6.2388515	-0.001092	h	1	0

Diastereomer **1d**_1

atomic	coordinates	atom	charge	isotop	
3.90080026	4.41707271	4.69247707	c	6	0
1.8704143	6.09060356	4.79949333	c	6	0
3.33220526	1.85926098	4.31019208	c	6	0
0.84107515	1.00580465	3.99629528	c	6	0
-1.1881576	2.72916492	4.05245338	c	6	0
-0.6192617	5.27270485	4.48473798	c	6	0
5.84320006	5.07846497	4.9133564	h	1	0
4.86517973	0.4697935	4.26174243	h	1	0
0.49985778	-1.0091636	3.71324107	h	1	0
-3.6577419	2.11384976	3.74908751	o	8	0
-4.3316216	-0.4317436	3.33783929	c	6	0
-6.4030433	-0.4638993	3.13876688	h	1	0
-3.7822528	-1.6483605	4.95080513	h	1	0
-3.4665452	-1.1873672	1.5878089	h	1	0
1.70295688	8.95195479	5.07478844	c	6	0
-2.2676919	7.61039743	4.61050319	c	6	0
1.70817453	10.1158678	2.4363174	c	6	0
-0.830079	9.29741957	6.03339479	o	8	0
3.04064043	9.81906661	6.38555053	h	1	0
-0.6125974	9.89485313	1.12803392	c	6	0
-2.6404759	8.66192343	2.11682119	o	8	0
-4.166934	7.39549993	5.41150712	h	1	0
3.75681305	11.3531523	1.25544524	c	6	0
3.45495851	12.4464826	-1.167439	c	6	0
1.1209114	12.2312129	-2.3919005	c	6	0
-0.8944351	10.9594901	-1.2774851	c	6	0
-2.7149891	10.7634004	-2.2344382	h	1	0
0.88237631	13.071621	-4.2670642	h	1	0
6.29943761	11.4069412	2.49786572	c	6	0
8.26862359	13.1475718	1.34349364	c	6	0
8.20408704	13.1087334	-1.5595236	c	6	0
10.1293503	12.6093876	2.10902488	h	1	0
7.83459684	15.0891552	1.98797707	h	1	0
5.55336831	13.9373696	-2.4294357	c	6	0
8.99848367	10.5487797	-2.6685043	c	6	0
9.5509109	14.5501318	-2.2605456	h	1	0
5.40227165	13.657072	-4.5022602	h	1	0
5.10871759	16.5377307	-1.8478121	o	8	0
6.80356248	10.102125	4.33139048	o	8	0
8.9657107	10.5929249	-4.7568295	h	1	0
7.73540266	9.00444635	-2.0507173	h	1	0
10.9376889	10.0562702	-2.0725868	h	1	0
6.34298045	17.5456386	-2.7662548	h	1	0

Diastereomer **1d**_2

atomic	coordinates	atom	charge	isotop	
3.86411369	4.40824332	4.74398295	c	6	0
1.86887011	6.1261845	4.78764748	c	6	0
3.24565705	1.85682149	4.39732952	c	6	0
0.74207281	1.0513395	4.05662823	c	6	0
-1.2500151	2.81819969	4.04796398	c	6	0
-0.6323396	5.35615382	4.44510567	c	6	0
5.81632686	5.03191751	4.98551847	h	1	0
4.74856545	0.43416156	4.39962072	h	1	0
0.36201728	-0.9606657	3.80368225	h	1	0
-3.7268821	2.25203504	3.71379312	o	8	0
-4.4513327	-0.2862394	3.34240346	c	6	0
-6.5194686	-0.2762194	3.11022513	h	1	0
-3.9550519	-1.4811045	4.9883021	h	1	0
-3.575618	-1.095927	1.62222888	h	1	0
1.75845786	8.99425226	5.01610778	c	6	0
-2.2318021	7.72863127	4.50450517	c	6	0
1.83143324	10.1226547	2.36345763	c	6	0
-0.7838686	9.41062259	5.92217968	o	8	0
3.09209083	9.8529464	6.33655738	h	1	0
-0.4713592	9.92080116	1.01287186	c	6	0
-2.5387555	8.74535615	1.98473085	o	8	0
-4.1493019	7.56848609	5.2737924	h	1	0
3.91868828	11.3145176	1.21139475	c	6	0
3.67861489	12.394424	-1.2278852	c	6	0
1.37497277	12.1661473	-2.5103616	c	6	0
-0.684058	10.9385785	-1.4185211	c	6	0
-2.4804316	10.7350051	-2.4185569	h	1	0
1.19275286	12.9475904	-4.4195198	h	1	0
6.45635191	11.3045437	2.4758808	c	6	0
8.48971355	12.9788963	1.33912623	c	6	0
8.43112264	13.0351132	-1.5673254	c	6	0
10.3281577	12.3428123	2.0835123	h	1	0
8.14995571	14.9202778	2.03797713	h	1	0
5.80493195	13.9157448	-2.4136703	c	6	0
9.19199376	10.5019423	-2.7543085	c	6	0
9.77140315	14.4981146	-2.2137563	h	1	0
5.66170656	13.7479555	-4.499065	h	1	0
5.60828086	16.5195581	-1.714771	o	8	0
6.89307938	9.9897346	4.3198962	o	8	0
9.16851672	10.6123543	-4.8400462	h	1	0
7.91037436	8.95164072	-2.1886055	h	1	0
11.1234699	9.96931438	-2.1683264	h	1	0
3.8429417	17.0145744	-1.8162567	h	1	0

Diastereomer **1d**_3

atomic	coordinates	atom	charge	isotop	
3.70278838	3.95469034	4.56487606	c	6	0
1.8201426	5.77810366	4.81915644	c	6	0
2.9147827	1.46543646	4.12339709	c	6	0
0.35192362	0.82376011	3.89267811	c	6	0
-1.5272865	2.69910111	4.09644655	c	6	0
-0.7382603	5.17196925	4.58824952	c	6	0
5.69989902	4.45218363	4.71971166	h	1	0
4.32752133	-0.0382577	3.96112526	h	1	0
-0.1622925	-1.1460758	3.55895166	h	1	0
-4.048899	2.29319966	3.88139505	o	8	0
-4.9425715	-0.1730937	3.41255945	c	6	0
-7.0161483	-0.0331551	3.30252559	h	1	0
-4.4285544	-1.4898803	4.95716126	h	1	0
-4.2131029	-0.92821	1.60162809	h	1	0
1.89483488	8.63227697	5.19525632	c	6	0
-2.1873103	7.62622988	4.86426868	c	6	0
1.88085374	9.88517959	2.59788169	c	6	0
-0.5593171	9.14100646	6.27804203	o	8	0
3.35290689	9.34249459	6.47183923	h	1	0
-0.5001882	9.89733141	1.39036696	c	6	0
-2.580145	8.79338306	2.42843393	o	8	0
-4.0611377	7.53123517	5.74445414	h	1	0
3.9720371	10.9996914	1.36422198	c	6	0
3.65278825	12.2147179	-1.0008217	c	6	0
1.25916434	12.2383815	-2.1264733	c	6	0
-0.7951509	11.0823398	-0.9565131	c	6	0
-2.6696121	11.0821506	-1.8265247	h	1	0
1.02240276	13.1902653	-3.9404027	h	1	0
6.54683308	10.8300771	2.50009528	c	6	0
8.64653406	12.3975191	1.29978373	c	6	0
7.74842396	14.5587417	-0.4001389	c	6	0
9.81768102	11.063334	0.18219847	h	1	0
9.87448588	13.0684387	2.84719403	h	1	0
5.87233083	13.458541	-2.3328588	c	6	0
9.96233533	15.9435631	-1.6495134	c	6	0
6.67884263	15.9342381	0.75556504	h	1	0
6.89804068	11.9828939	-3.433835	h	1	0
4.9193703	15.3091114	-4.0218168	o	8	0
7.03664704	9.44777731	4.28118758	o	8	0
11.0471135	14.6707175	-2.9084491	h	1	0
11.2865579	16.6772677	-0.2119752	h	1	0
9.30233416	17.5763208	-2.7703687	h	1	0
6.24477262	15.7499636	-5.2088285	h	1	0

Cartesian coordinates of the conformers shown in Table S5.

Diastereomer **2a**/**3b**_1

atomic coordinates			atom	charge	isotop
-4.29116984	2.04424302	0.26822877	c	6.000	0
-6.56263503	0.98774449	-0.56319812	c	6.000	0
-1.98263994	0.89043330	-0.41724914	c	6.000	0
-6.55369991	-1.19160142	-2.03450840	c	6.000	0
-1.93420105	-1.26744907	-1.99128525	c	6.000	0
-4.26443221	-2.30879986	-2.74996821	c	6.000	0
-4.28595906	4.44411131	1.87327920	c	6.000	0
-2.14062876	6.19386673	0.98656185	c	6.000	0
0.37729314	4.79395486	1.33159342	c	6.000	0
-2.14736999	8.74647466	2.34194529	c	6.000	0
-2.46056360	6.52698513	-1.04964755	h	1.000	0
0.35958389	1.96976438	0.76154472	c	6.000	0
2.14421953	0.65819649	1.35541595	o	8.000	0
0.43483734	-2.45496828	-3.11563779	c	6.000	0
-4.20567380	-4.43060192	-4.21221396	o	8.000	0
-8.33554769	-2.02202664	-2.68113676	h	1.000	0
-8.34134682	1.90842755	-0.07658661	h	1.000	0
-5.89973849	-4.86657850	-4.73547862	h	1.000	0
-1.87928774	8.50337931	4.39670340	h	1.000	0
-3.94725426	9.73756061	2.03981479	h	1.000	0
-0.60131047	9.95608552	1.64660222	h	1.000	0
2.28984529	-0.61994214	-3.81205176	o	8.000	0
-0.17684873	-3.39516072	-4.87251050	h	1.000	0
1.86849879	-4.37969757	-1.55365532	c	6.000	0
4.41272179	-3.72473991	-1.62878884	c	6.000	0
4.68570879	-1.32332351	-3.04938370	c	6.000	0
6.50642687	-0.08624730	-3.57275529	o	8.000	0
1.01583550	-6.53540830	-0.30510909	c	6.000	0
2.83444402	-8.01793142	0.91063822	c	6.000	0
6.24975045	-5.21656014	-0.40864554	c	6.000	0
5.40958242	-7.39125770	0.87116312	c	6.000	0
-0.98269738	-7.04584676	-0.27338353	h	1.000	0
2.24868025	-9.72031134	1.92626492	h	1.000	0
8.67349357	-4.46498275	-0.54877181	o	8.000	0
6.75094008	-8.61040935	1.84963576	h	1.000	0
10.56991878	-5.87983569	0.67976219	c	6.000	0
12.36115613	-4.89898399	0.31085335	h	1.000	0
10.69452608	-7.82544625	-0.06942572	h	1.000	0
10.25630856	-5.96745774	2.74247665	h	1.000	0
1.06689655	4.98458946	3.29269162	h	1.000	0
1.87034569	5.62105153	0.13175772	h	1.000	0
-3.88726774	3.93038390	3.86872004	h	1.000	0
-6.63375660	5.72997243	1.73296556	o	8.000	0
-7.63830440	5.29443517	3.18867823	h	1.000	0

Diastereomer **2a**/**3b**_2

atomic coordinates			atom	charge	isotop
-2.26578163	-0.94599690	-3.87639522	o	8.000	0
-2.40070808	-2.81342427	-2.50728863	c	6.000	0
-0.60225572	-3.19552689	-0.33202488	c	6.000	0
-1.01138143	-5.32846078	1.24022726	c	6.000	0
-3.06948216	-7.23935185	0.73340271	c	6.000	0
-2.03202251	-9.23584751	-0.75229997	o	8.000	0
-5.32524825	-6.12535829	-0.67652196	c	6.000	0
-6.92055505	-4.38114107	0.99456286	c	6.000	0
-4.39361326	-4.79026678	-3.04907312	c	6.000	0
0.56616195	-5.75610580	3.32535108	c	6.000	0
2.52958740	-4.10939845	3.85088392	c	6.000	0
2.96063393	-2.03580196	2.29204883	c	6.000	0
5.00267199	-0.61264921	2.98671215	o	8.000	0
1.42296378	-1.48570269	0.18481522	c	6.000	0
1.86629353	0.88836026	-1.38781487	c	6.000	0
4.51455573	1.63971537	-1.33357973	o	8.000	0
4.81332144	4.16892482	-0.85717977	c	6.000	0
6.83324970	5.25306071	-0.87853368	o	8.000	0
1.53389070	0.55368976	-3.39470403	h	1.000	0
0.57428777	3.21518004	-0.47526612	c	6.000	0
2.31510349	5.15536187	-0.22657816	c	6.000	0
1.65199859	7.57912461	0.54480804	c	6.000	0
3.53548862	9.32371977	0.73207990	o	8.000	0
2.86765941	11.80077279	1.52538694	c	6.000	0
-0.90026553	8.01735210	1.06580554	c	6.000	0
-2.68926926	6.07206801	0.80615717	c	6.000	0
-1.95870114	3.66002158	0.02626719	c	6.000	0
-3.72616199	-8.07404388	2.51333576	h	1.000	0
-6.55432612	-7.68211468	-1.28936014	h	1.000	0
-0.29857673	-9.52346382	-0.21032652	h	1.000	0
-7.62296625	-5.40707339	2.64920707	h	1.000	0
-5.85815102	-2.74104776	1.67127379	h	1.000	0
-8.56348295	-3.67948576	-0.05083363	h	1.000	0
-5.98948698	-3.86165535	-3.99015673	h	1.000	0
-3.58443253	-6.14028713	-4.39644785	h	1.000	0
0.27608899	-7.38145925	4.55272820	h	1.000	0
3.75772042	-4.44539176	5.46508798	h	1.000	0
5.72171279	0.16365028	1.47455330	h	1.000	0
1.56544913	12.68743229	0.18387035	h	1.000	0
2.07416340	11.78300936	3.43627800	h	1.000	0
4.59430218	12.93763203	1.57130728	h	1.000	0
-1.57508673	9.85681151	1.67448633	h	1.000	0
-4.67008019	6.45624934	1.20413349	h	1.000	0
-3.37164937	2.19038156	-0.19955508	h	1.000	0

Diastereomer **2a**/**3b**_3

atomic coordinates			atom	charge	isotop
-2.26653753	-1.00627917	-3.85390748	o	8.000	0
-2.22987684	-2.89354866	-2.50709966	c	6.000	0
-0.45296735	-3.12126066	-0.29895467	c	6.000	0
-0.79368498	-5.21715591	1.33584740	c	6.000	0
-2.76221269	-7.23330472	0.88892717	c	6.000	0
-1.50970221	-9.24813073	-0.38607105	o	8.000	0
-4.99038877	-6.33303919	-0.70883627	c	6.000	0
-6.77958148	-4.61074279	0.77894511	c	6.000	0
-3.99866050	-5.05917481	-3.09102504	c	6.000	0
0.78801580	-5.50798476	3.44251410	c	6.000	0
2.66545871	-3.75450788	3.94310255	c	6.000	0
3.01619188	-1.71209188	2.32663082	c	6.000	0
4.97810555	-0.16383926	2.98671215	o	8.000	0
1.48475782	-1.30504487	0.18368138	c	6.000	0
1.84550654	1.02914485	-1.46415981	c	6.000	0
4.47468251	1.86024641	-1.45244351	o	8.000	0
4.70258348	4.39833758	-0.99664156	c	6.000	0
6.68604003	5.54899182	-0.98794882	o	8.000	0
1.50535584	0.62984572	-3.45800985	h	1.000	0
0.49076188	3.34046889	-0.60206675	c	6.000	0
2.18395649	5.32090188	-0.38285851	c	6.000	0
1.43090063	7.71858639	0.35167803	c	6.000	0
3.05776586	9.71054671	0.49888770	o	8.000	0
4.24583668	9.78651370	2.92113866	c	6.000	0
-1.13100109	8.14131813	0.80615717	c	6.000	0
-2.86709249	6.15275932	0.58222462	c	6.000	0
-2.06017943	3.73731138	-0.12528884	c	6.000	0
-3.45385245	-7.96255004	2.70230837	h	1.000	0
-6.08964247	-7.98881723	-1.30731254	h	1.000	0
-2.65034090	-10.68866896	-0.40213372	h	1.000	0
-7.53641679	-5.60643949	2.42829808	h	1.000	0
-5.84737958	-2.89203688	1.45301042	h	1.000	0
-8.38320308	-4.03947858	-0.39835427	h	1.000	0
-5.58999888	-4.32237059	-4.19538099	h	1.000	0
-2.98142092	-6.40088036	-4.29780415	h	1.000	0
0.57750031	-7.12823595	4.69162307	h	1.000	0
3.89453659	-3.99469207	5.57242442	h	1.000	0
5.74986971	0.46808516	1.43316830	h	1.000	0
2.85254160	10.15066393	4.40873107	h	1.000	0
5.28235146	8.04248545	3.32818567	h	1.000	0
5.59906956	11.34950619	2.91376873	h	1.000	0
-1.78484633	10.01951693	1.32072959	h	1.000	0
-4.86264329	6.50103585	0.93352471	h	1.000	0
-3.43249855	2.22590841	-0.33259180	h	1.000	0

Diastereomer **2a**/**3b**_4

atomic coordinates			atom	charge	isotop
-2.23025478	-1.05484513	-3.98051913	o	8.000	0
-2.36933863	-2.86558071	-2.53790220	c	6.000	0
-0.58675996	-3.14866168	-0.33467050	c	6.000	0
-0.97717738	-5.23605317	1.30221028	c	6.000	0
-3.00579839	-7.18965205	0.84187299	c	6.000	0
-1.93337881	-9.21203696	-0.58184668	o	8.000	0
-5.26987927	-6.14614528	-0.60830284	c	6.000	0
-6.89145327	-4.37055860	1.00363355	c	6.000	0
-4.34693703	-4.87379267	-3.01808161	c	6.000	0
0.58978353	-5.57412518	3.41152259	c	6.000	0
2.52505206	-3.88300926	3.89944988	c	6.000	0
2.93682338	-1.85363236	2.27900972	c	6.000	0
4.95164939	-0.37624447	2.93984695	o	8.000	0
1.40709008	-1.39216124	0.14437508	c	6.000	0
1.82075113	0.94618588	-1.48986008	c	6.000	0
4.45710806	1.75177613	-1.42787707	o	8.000	0
4.70352835	4.28457606	-0.95223300	c	6.000	0
6.69983504	5.41104181	-0.89497430	o	8.000	0
1.51669420	0.55633537	-3.49089109	h	1.000	0
0.47394331	3.26865929	-0.64420764	c	6.000	0
2.18357855	5.23038399	-0.38361441	c	6.000	0
1.44280590	7.63392666	0.34506399	c	6.000	0
3.08913531	9.60642280	0.52874537	o	8.000	0
4.22240407	9.67067349	2.97745250	c	6.000	0
-1.12211938	8.08349251	0.75078819	c	6.000	0
-2.87540729	6.11534274	0.48395886	c	6.000	0
-2.08153334	3.69365870	-0.21788542	c	6.000	0
-3.65945466	-7.98239216	2.64202611	h	1.000	0
-6.47835913	-7.73559393	-1.17616555	h	1.000	0
-0.20409042	-9.47036252	-0.01285014	h	1.000	0
-7.58422687	-5.35227133	2.68889132	h	1.000	0
-5.85191492	-2.69588330	1.62951085	h	1.000	0
-8.54080623	-3.72389432	-0.06689631	h	1.000	0
-5.94942479	-3.98637728	-3.98751112	h	1.000	0
-3.52622897	-6.25745015	-4.32369339	h	1.000	0
0.31293865	-7.16414075	4.68765465	h	1.000	0
3.74505925	-4.15059448	5.53236223	h	1.000	0
5.69884710	0.30972611	1.39707453	h	1.000	0
5.22849427	7.91455099	3.40831006	h	1.000	0
5.59415627	11.21741433	2.99994024	h	1.000	0
2.80019619	10.05239817	4.43273059	h	1.000	0
-1.76576010	9.96698255	1.25874658	h	1.000	0
-4.87379267	6.48535112	0.79500778	h	1.000	0
-3.46915924	2.20209786	-0.46147112	h	1.000	0

Diastereomer **2a**/**3b**_5

atomic coordinates			atom	charge	isotop
-2.22269588	-0.83280231	-3.84370296	o	8.000	0
-2.38011007	-2.71988282	-2.50350918	c	6.000	0
-0.58033490	-3.15886621	-0.33939481	c	6.000	0
-0.97698841	-5.34168886	1.16577205	c	6.000	0
-3.03376634	-7.23859596	0.60641312	c	6.000	0
-2.00367662	-9.17310860	-0.96319341	o	8.000	0
-5.30370537	-6.07546952	-0.73944984	c	6.000	0
-6.87860313	-4.38718819	1.00684608	c	6.000	0
-4.39814860	-4.66271026	-3.07647415	c	6.000	0
0.60924771	-5.83056101	3.23010888	c	6.000	0
2.56870473	-4.19443613	3.80137309	c	6.000	0
2.98520037	-2.06830525	2.31075712	c	6.000	0
5.02364795	-0.66064826	3.04604956	o	8.000	0
1.43713672	-1.45565604	0.22865686	c	6.000	0
1.85665593	0.97453177	-1.26177014	c	6.000	0
4.49490258	1.76953955	-1.16388233	o	8.000	0
4.74302362	4.28627682	-0.61094846	c	6.000	0
6.72648017	5.43598620	-0.61794045	o	8.000	0
1.54655187	0.69938764	-3.28132046	h	1.000	0
0.51967469	3.25146279	-0.28629351	c	6.000	0
2.23044376	5.20373885	0.03269226	c	6.000	0
1.49893077	7.57213262	0.87645498	c	6.000	0
3.17530682	9.47792142	1.31695014	o	8.000	0
3.63413233	10.87990924	-0.94278437	c	6.000	0
-1.04123910	7.96273901	1.45962447	c	6.000	0
-2.79887338	6.01008500	1.11871787	c	6.000	0
-2.02427463	3.64395890	0.23054659	c	6.000	0
-3.67306069	-8.14188505	2.35894513	h	1.000	0
-6.54147599	-7.60992714	-1.38989357	h	1.000	0
-0.27533310	-9.49738560	-0.42481043	h	1.000	0
-7.55871556	-5.46565490	2.63711282	h	1.000	0
-5.80882916	-2.76788187	1.72116256	h	1.000	0
-8.53513706	-3.65435240	0.00548021	h	1.000	0
-6.00139226	-3.69006822	-3.95935420	h	1.000	0
-3.62128219	-5.97153458	-4.48224142	h	1.000	0
0.32900132	-7.49522076	4.40627443	h	1.000	0
3.80364076	-4.57786156	5.39970346	h	1.000	0
5.72209073	0.18727186	1.56355940	h	1.000	0
1.91051312	11.83781142	-1.57357495	h	1.000	0
5.05067104	12.32403795	-0.51551729	h	1.000	0
4.37698367	9.68068904	-2.45664397	h	1.000	0
-1.65955749	9.78311220	2.18357855	h	1.000	0
-4.78119609	6.33984221	1.55354385	h	1.000	0
-3.41397923	2.16052389	-0.04629829	h	1.000	0

Diastereomer **2a**/**3b**_6

atomic coordinates			atom	charge	isotop
-2.24650643	-0.79368498	-3.71728028	o	8.000	0
-2.23951444	-2.74955152	-2.47062795	c	6.000	0
-0.44144002	-3.13505566	-0.30046646	c	6.000	0
-0.78896066	-5.32562619	1.20356657	c	6.000	0
-2.79169242	-7.28243760	0.65951442	c	6.000	0
-1.59133838	-9.22280840	-0.77138621	o	8.000	0
-5.03404145	-6.24611179	-0.83223539	c	6.000	0
-6.76503059	-4.59430218	0.79727546	c	6.000	0
-4.06593475	-4.83921068	-3.14809477	c	6.000	0
0.81636169	-5.76914491	3.26469087	c	6.000	0
2.72233947	-4.07349365	3.84899419	c	6.000	0
3.07817490	-1.93489059	2.36291356	c	6.000	0
5.06692268	-0.45825859	3.09990675	o	8.000	0
1.52444207	-1.37458679	0.27155365	c	6.000	0
1.89123791	1.05238848	-1.23266836	c	6.000	0
4.52154772	1.87763189	-1.18277959	o	8.000	0
4.74793691	4.40079422	-0.65119963	c	6.000	0
6.71551976	5.57695977	-0.70335607	o	8.000	0
1.54655187	0.76666189	-3.24560464	h	1.000	0
0.54443010	3.31722526	-0.24320775	c	6.000	0
2.23630191	5.29236701	0.03231432	c	6.000	0
1.48967111	7.65603646	0.87626601	c	6.000	0
3.14714990	9.58752554	1.27310850	o	8.000	0
3.55381897	10.97326171	-1.00665711	c	6.000	0
-1.04426266	8.01640723	1.50648967	c	6.000	0
-2.78205481	6.03861986	1.21207034	c	6.000	0
-1.99366107	3.67797397	0.32219831	c	6.000	0
-3.46197828	-8.12298779	2.43245548	h	1.000	0
-6.17241247	-7.84274140	-1.51253680	h	1.000	0
-2.74709488	-10.64936266	-0.84962087	h	1.000	0
-7.50466940	-5.67976087	2.39692863	h	1.000	0
-5.79238855	-2.93474469	1.55675639	h	1.000	0
-8.38263616	-3.92703988	-0.30840331	h	1.000	0
-5.66502100	-4.00225098	-4.16684613	h	1.000	0
-3.10293031	-6.12025603	-4.46088751	h	1.000	0
0.60112188	-7.46196159	4.41269950	h	1.000	0
3.96918077	-4.43084087	5.44335613	h	1.000	0
5.78350683	0.34052865	1.59908625	h	1.000	0
1.80827894	11.90262903	-1.62025119	h	1.000	0
4.95750754	12.43968919	-0.61453894	h	1.000	0
4.28930038	9.76969514	-2.52051672	h	1.000	0
-1.67202968	9.83262302	2.23233348	h	1.000	0
-4.75946424	6.34381063	1.68544674	h	1.000	0
-3.36579122	2.17129533	0.08295898	h	1.000	0

Diastereomer **2a**/**3b**_7

atomic coordinates			atom	charge	isotop
-2.91735921	-0.34128454	-3.26998210	o	8.000	0
-2.68265522	-2.42999884	-2.28713554	c	6.000	0
-0.71034805	-2.91169003	-0.30235618	c	6.000	0
-0.97207512	-5.13778741	1.16860664	c	6.000	0
-3.05984456	-7.03885190	0.67141970	c	6.000	0
-2.26275807	-9.48812595	1.45735679	o	8.000	0
-3.69876096	-7.11330711	-2.14824067	c	6.000	0
-5.75421608	-9.05292202	-2.76788187	c	6.000	0
-4.53420889	-4.49320183	-2.97745250	c	6.000	0
0.69390744	-5.55371613	3.18815696	c	6.000	0
2.64164816	-3.87318268	3.68175343	c	6.000	0
2.96365750	-1.75631147	2.16071286	c	6.000	0
5.01098679	-0.30367899	2.77185029	o	8.000	0
1.30958021	-1.18088986	0.15571343	c	6.000	0
1.64406174	1.23096760	-1.37779932	c	6.000	0
4.30895353	1.86497072	-1.64519557	o	8.000	0
4.77307027	4.36980271	-1.16879561	c	6.000	0
6.81359655	5.37797160	-1.44620741	o	8.000	0
1.03103458	0.99059444	-3.33290998	h	1.000	0
0.58846072	3.57214931	-0.23319220	c	6.000	0
2.42659733	5.43579722	-0.18065782	c	6.000	0
1.97608662	7.85710332	0.73680422	c	6.000	0
3.93686645	9.52478663	0.70278915	o	8.000	0
3.49523746	11.98880054	1.67448633	c	6.000	0
-0.46014831	8.37280958	1.61911735	c	6.000	0
-2.34552808	6.50273660	1.56563810	c	6.000	0
-1.82925490	4.09050119	0.63116853	c	6.000	0
-4.70900855	-6.50254763	1.80865688	h	1.000	0
-2.00103100	-7.65830413	-3.21499107	h	1.000	0
-3.76112192	-10.53616806	1.63083365	h	1.000	0
-5.11076433	-10.97911987	-2.37557472	h	1.000	0
-7.46914254	-8.70861392	-1.66144722	h	1.000	0
-6.25933987	-8.96882920	-4.77344821	h	1.000	0
-6.35684974	-3.99866050	-2.12235142	h	1.000	0
-4.76362164	-4.46731258	-5.03687604	h	1.000	0
0.50436791	-7.22347815	4.37547189	h	1.000	0
3.94555920	-4.23374243	5.22868324	h	1.000	0
5.71339799	0.38172468	1.20904678	h	1.000	0
2.05753381	12.98033984	0.56465017	h	1.000	0
2.97745250	11.91944759	3.67665117	h	1.000	0
5.25891886	13.05668478	1.51707214	h	1.000	0
-0.96848464	10.21604845	2.36272459	h	1.000	0
-4.23355346	6.94549943	2.25009691	h	1.000	0
-3.31250094	2.67396248	0.57258702	h	1.000	0

Diastereomer **2a**/**3b**_8

atomic coordinates			atom	charge	isotop
-2.80397564	-0.43520393	-3.45063992	o	8.000	0
-2.82910900	-2.38407849	-2.19491690	c	6.000	0
-0.84319580	-2.91905996	-0.22903481	c	6.000	0
-1.16407130	-5.11511070	1.27858870	c	6.000	0
-3.30418615	-6.97535711	0.85812464	c	6.000	0
-2.48517884	-9.49171643	1.38063391	o	8.000	0
-4.26681264	-6.89750039	-1.86534867	c	6.000	0
-6.50783886	-8.68310261	-2.26313602	c	6.000	0
-5.00645145	-4.20293989	-2.51125706	c	6.000	0
0.49284058	-5.55484997	3.30210745	c	6.000	0
2.50993425	-3.94744892	3.74543720	c	6.000	0
2.90375318	-1.87441935	2.18036601	c	6.000	0
5.01457727	-0.50172229	2.75918913	o	8.000	0
1.25137665	-1.26252603	0.18179165	c	6.000	0
1.65653393	1.12533191	-1.37817727	c	6.000	0
4.33370894	1.73420167	-1.57867721	o	8.000	0
4.80973096	4.23903366	-1.11456047	c	6.000	0
6.86877655	5.22131331	-1.33981583	o	8.000	0
1.10643465	0.85869156	-3.34746087	h	1.000	0
0.58997250	3.49315876	-0.29876570	c	6.000	0
2.44511664	5.33904325	-0.21164933	c	6.000	0
1.99290518	7.77735688	0.65932545	c	6.000	0
3.97239331	9.42293039	0.66669538	o	8.000	0
3.52622897	11.90659745	1.58510228	c	6.000	0
-0.46392777	8.33085766	1.45660090	c	6.000	0
-2.36744890	6.48119372	1.36513816	c	6.000	0
-1.84871908	4.05119489	0.47866763	c	6.000	0
-4.83202972	-6.52635818	2.18509033	h	1.000	0
-2.74974050	-7.51751953	-3.14261456	h	1.000	0
-0.85831361	-9.73775877	0.55879202	h	1.000	0
-5.97172356	-10.64879574	-1.90503292	h	1.000	0
-8.07952409	-8.20745854	-1.00306663	h	1.000	0
-7.19569917	-8.57009699	-4.21201058	h	1.000	0
-6.57246749	-3.53794527	-1.32866644	h	1.000	0
-5.61815580	-4.11657941	-4.48980032	h	1.000	0
0.22412152	-7.17396732	4.54554724	h	1.000	0
3.80534152	-4.32048086	5.29728030	h	1.000	0
5.68920950	0.21883029	1.19997610	h	1.000	0
2.94343743	11.87447211	3.57025958	h	1.000	0
5.30559509	12.95369470	1.46831721	h	1.000	0
2.13595745	12.89530217	0.41403900	h	1.000	0
-0.97528766	10.18940331	2.15920108	h	1.000	0
-4.27172593	6.95457012	1.98099991	h	1.000	0
-3.34878368	2.65487625	0.38852769	h	1.000	0

Diastereomer **2a**/**3b**_9

atomic coordinates			atom	charge	isotop
-3.30172950	-0.81239326	-3.18286573	o	8.000	0
-2.67585221	-2.88126544	-2.34458321	c	6.000	0
-0.71507237	-3.12749675	-0.32144242	c	6.000	0
-0.75815812	-5.35680667	1.15707931	c	6.000	0
-2.47421843	-7.56608549	0.58090181	c	6.000	0
-0.96149266	-9.35830176	-0.74833155	o	8.000	0
-4.77628280	-6.89598861	-1.03764862	c	6.000	0
-6.85838306	-5.62571470	0.52345414	c	6.000	0
-3.94877173	-5.26156447	-3.26280114	c	6.000	0
0.91746204	-5.59226655	3.19855045	c	6.000	0
2.56284658	-3.64188021	3.80893200	c	6.000	0
2.61386919	-1.46793926	2.32606390	c	6.000	0
4.21692387	0.44011722	3.00863298	o	8.000	0
1.05276643	-1.17295301	0.18953953	c	6.000	0
1.32923336	1.13345774	-1.50705659	c	6.000	0
3.97296022	1.64765222	-2.04014833	o	8.000	0
4.64381300	4.06310016	-1.40349960	c	6.000	0
6.73762956	4.96279877	-1.68979311	o	8.000	0
0.53422558	0.83166847	-3.38468848	h	1.000	0
0.41744050	3.55778739	-0.42783400	c	6.000	0
2.38218876	5.28594194	-0.37889009	c	6.000	0
2.07907669	7.73672776	0.51797393	c	6.000	0
4.13944510	9.28101196	0.48736037	o	8.000	0
3.82480570	11.79793820	1.36551610	c	6.000	0
-0.32956824	8.41324972	1.37723241	c	6.000	0
-2.33834712	6.67300092	1.31430453	c	6.000	0
-1.97400792	4.22750633	0.40137783	c	6.000	0
-3.09385963	-8.45387883	2.34911856	h	1.000	0
-5.57658182	-8.64455220	-1.81810551	h	1.000	0
-1.93507956	-10.91033383	-0.89081690	h	1.000	0
-7.51449597	-6.89579964	2.02030621	h	1.000	0
-6.21719898	-3.87601727	1.42107405	h	1.000	0
-8.48694904	-5.16915687	-0.66952997	h	1.000	0
-5.59472319	-4.73565369	-4.40608545	h	1.000	0
-2.63276645	-6.26595391	-4.50737477	h	1.000	0
0.93749314	-7.31097247	4.32879565	h	1.000	0
3.80042823	-3.84823830	5.43352955	h	1.000	0
5.46452106	-0.19029542	4.19916044	h	1.000	0
3.28434402	11.82855176	3.36257868	h	1.000	0
5.64650169	12.76113161	1.19241719	h	1.000	0
2.45569911	12.82783894	0.20503529	h	1.000	0
-0.72225333	10.29031469	2.10572183	h	1.000	0
-4.19897147	7.24483205	1.97646456	h	1.000	0
-3.53303198	2.89392660	0.32956824	h	1.000	0

Diastereomer **2a**/**3b**_10

atomic coordinates			atom	charge	isotop
-3.29303676	-0.86795121	-3.26317909	o	8.000	0
-2.64807323	-2.90620982	-2.36631506	c	6.000	0
-0.70373401	-3.07307264	-0.31917474	c	6.000	0
-0.72546586	-5.26836749	1.20980267	c	6.000	0
-2.40203089	-7.51714159	0.67047483	c	6.000	0
-0.84962087	-9.31521600	-0.60395647	o	8.000	0
-4.70012684	-6.92017710	-0.98190170	c	6.000	0
-6.81435244	-5.64555683	0.53157996	c	6.000	0
-3.87847392	-5.32600414	-3.23804573	c	6.000	0
0.93522546	-5.43107291	3.26998210	c	6.000	0
2.54565008	-3.44232513	3.84880522	c	6.000	0
2.57701953	-1.30126542	2.31737116	c	6.000	0
4.14946064	0.64307380	2.97235024	o	8.000	0
1.02876691	-1.07884465	0.16232747	c	6.000	0
1.27858870	1.19392897	-1.58264564	c	6.000	0
3.92080378	1.74969743	-2.09740704	o	8.000	0
4.53647656	4.18555441	-1.49250570	c	6.000	0
6.61744298	5.12814981	-1.73098914	o	8.000	0
0.51268270	0.83733765	-3.46292314	h	1.000	0
0.30897022	3.62222705	-0.56219352	c	6.000	0
2.24575054	5.37721571	-0.51684010	c	6.000	0
1.86081332	7.82044263	0.33958379	c	6.000	0
3.70121761	9.62399725	0.33315872	o	8.000	0
5.24039954	9.41423765	2.53941398	c	6.000	0
-0.57126421	8.51453904	1.09434040	c	6.000	0
-2.54621699	6.74556641	1.03972732	c	6.000	0
-2.11195793	4.27758408	0.20730296	c	6.000	0
-3.02337284	-8.37375444	2.45324247	h	1.000	0
-5.46754462	-8.69859837	-1.72758763	h	1.000	0
-1.80090901	-10.88236589	-0.72754456	h	1.000	0
-7.46385131	-6.88994148	2.05299847	h	1.000	0
-6.20718343	-3.86562378	1.39272816	h	1.000	0
-8.43951691	-5.24228927	-0.68483675	h	1.000	0
-5.52253565	-4.85168288	-4.40627443	h	1.000	0
-2.53752425	-6.33946426	-4.44822635	h	1.000	0
0.97113026	-7.12313369	4.43972258	h	1.000	0
3.77113747	-3.59407013	5.48870956	h	1.000	0
5.40669544	0.03817247	4.16684613	h	1.000	0
4.13226414	9.81202501	4.24262414	h	1.000	0
6.13329514	7.55436919	2.69304871	h	1.000	0
6.73762956	10.83342198	2.40373164	h	1.000	0
-0.93919389	10.43884716	1.71171393	h	1.000	0
-4.43405340	7.30927171	1.62535345	h	1.000	0
-3.64943911	2.91849304	0.13114699	h	1.000	0

Diastereomer **2a**/**3b**_11

atomic coordinates			atom	charge	isotop
-3.29020217	-0.69730894	-3.12485114	o	8.000	0
-2.66923816	-2.78734605	-2.33626842	c	6.000	0
-0.69919867	-3.08800148	-0.32843440	c	6.000	0
-0.72981223	-5.35983023	1.08413588	c	6.000	0
-2.44511664	-7.55455817	0.45315633	c	6.000	0
-0.93994978	-9.30028717	-0.94448512	o	8.000	0
-4.76173191	-6.83835196	-1.12400910	c	6.000	0
-6.82984819	-5.61437634	0.49151777	c	6.000	0
-3.95406296	-5.14005508	-3.30815457	c	6.000	0
0.95620142	-5.65198189	3.10954435	c	6.000	0
2.60026316	-3.71671336	3.77000364	c	6.000	0
2.63862460	-1.49911974	2.35308698	c	6.000	0
4.23978955	0.39287406	3.08271024	o	8.000	0
1.06637246	-1.14479609	0.23319220	c	6.000	0
1.32072959	1.21811747	-1.38687001	c	6.000	0
3.96124392	1.77728743	-1.90805648	o	8.000	0
4.60639642	4.17459400	-1.19033849	c	6.000	0
6.67356784	5.13570871	-1.47228563	o	8.000	0
0.52723359	0.97226410	-3.27300566	h	1.000	0
0.38852769	3.59709370	-0.22431049	c	6.000	0
2.34004787	5.33356304	-0.12283220	c	6.000	0
1.99063751	7.73181448	0.86587251	c	6.000	0
3.89642632	9.45184320	1.09131684	o	8.000	0
4.20142811	10.83474479	-1.20564527	c	6.000	0
-0.39003947	8.36014842	1.81678271	c	6.000	0
-2.38445644	6.61649811	1.69073797	c	6.000	0
-2.00424354	4.21786873	0.65365627	c	6.000	0
-3.04869517	-8.49904329	2.19737355	h	1.000	0
-5.56864497	-8.56386089	-1.94792970	h	1.000	0
-1.90975723	-10.85061849	-1.12382013	h	1.000	0
-7.47122124	-6.92660217	1.95813422	h	1.000	0
-6.18148316	-3.89000125	1.43203446	h	1.000	0
-8.46975253	-5.12588214	-0.67293148	h	1.000	0
-5.61078586	-4.58050718	-4.41912456	h	1.000	0
-2.65128577	-6.10910665	-4.59430218	h	1.000	0
0.98587012	-7.40338007	4.18801106	h	1.000	0
3.84729344	-3.97031461	5.38061722	h	1.000	0
5.43485236	-0.24037316	4.32444928	h	1.000	0
5.81185272	12.10936507	-0.96772875	h	1.000	0
4.59827060	9.59395061	-2.81285735	h	1.000	0
2.52448514	11.98539903	-1.59322810	h	1.000	0
-0.69976559	10.20867852	2.65714392	h	1.000	0
-4.23714394	7.13522794	2.41412514	h	1.000	0
-3.55192924	2.87276167	0.54461907	h	1.000	0

Diastereomer **2a**/**3b**_12

atomic coordinates			atom	charge	isotop
-2.89789503	-0.42254276	-3.40660930	o	8.000	0
-2.65960056	-2.46949411	-2.34061479	c	6.000	0
-0.70430093	-2.86010050	-0.31898577	c	6.000	0
-0.94542998	-5.04708056	1.21320418	c	6.000	0
-2.99634976	-6.99708896	0.75248895	c	6.000	0
-2.15882314	-9.41083615	1.60399954	o	8.000	0
-3.62128219	-7.15563698	-2.06660450	c	6.000	0
-5.64612374	-9.14022736	-2.64240405	c	6.000	0
-4.49055621	-4.57068060	-2.96762592	c	6.000	0
0.70486785	-5.37702674	3.26204525	c	6.000	0
2.61972734	-3.65170678	3.72805172	c	6.000	0
2.92283941	-1.57470879	2.15013040	c	6.000	0
4.94031103	-0.06973089	2.73575652	o	8.000	0
1.28104535	-1.08300205	0.11224973	c	6.000	0
1.58888173	1.29729699	-1.47663200	c	6.000	0
4.24848229	1.97854326	-1.72210743	o	8.000	0
4.66724561	4.48658779	-1.23191247	c	6.000	0
6.70134682	5.52896072	-1.41540487	o	8.000	0
1.00949170	1.00306663	-3.43419930	h	1.000	0
0.47243153	3.64112431	-0.39344098	c	6.000	0
2.28826938	5.52083490	-0.31237173	c	6.000	0
1.75801222	7.92645627	0.57409880	c	6.000	0
3.50109561	9.82298542	0.59280709	o	8.000	0
4.94824788	9.75627808	2.87030502	c	6.000	0
-0.70826935	8.46483924	1.33357973	c	6.000	0
-2.56643706	6.57756975	1.25477815	c	6.000	0
-1.98099991	4.15021654	0.38814975	c	6.000	0
-4.66063156	-6.46248543	1.86856120	h	1.000	0
-1.91108004	-7.70403550	-3.11143408	h	1.000	0
-3.63847870	-10.48249984	1.79335010	h	1.000	0
-4.97734966	-11.04582720	-2.19548382	h	1.000	0
-7.37106576	-8.79062803	-1.55297694	h	1.000	0
-6.14312172	-9.11736168	-4.65174985	h	1.000	0
-6.31716549	-4.07235982	-2.12386320	h	1.000	0
-4.72337047	-4.60620745	-5.02667152	h	1.000	0
0.52855640	-7.01541930	4.49452464	h	1.000	0
3.91211104	-3.94725995	5.29822516	h	1.000	0
5.67957190	0.51967469	1.15008733	h	1.000	0
6.37895954	11.24424844	2.75503173	h	1.000	0
3.74846076	10.14480578	4.51209909	h	1.000	0
5.92259068	7.94667634	3.11237894	h	1.000	0
-1.19392897	10.35437641	1.97646456	h	1.000	0
-4.48602087	7.01523033	1.84720730	h	1.000	0
-3.44704944	2.71685926	0.29971056	h	1.000	0

Diastereomer **2a**/**3b**_13

atomic coordinates			atom	charge	isotop
-3.40056218	-0.78877169	-3.20516450	o	8.000	0
-2.85669900	-2.87276167	-2.34647294	c	6.000	0
-0.87229758	-3.16944867	-0.34638680	c	6.000	0
-0.92842245	-5.40801825	1.11947376	c	6.000	0
-2.64693940	-7.60576974	0.49775386	c	6.000	0
-1.25326637	-9.38910430	-0.97453177	o	8.000	0
-5.00588453	-6.86537504	-0.99928718	c	6.000	0
-6.99841176	-5.59018785	0.67009689	c	6.000	0
-4.24375798	-5.20468372	-3.22859710	c	6.000	0
0.72376511	-5.66709970	3.17719655	c	6.000	0
2.38559027	-3.73579959	3.79967234	c	6.000	0
2.47421843	-1.56355940	2.31548143	c	6.000	0
4.09862701	0.32333214	3.00485353	o	8.000	0
0.92483197	-1.24457363	0.17196508	c	6.000	0
1.24627439	1.05956944	-1.52236337	c	6.000	0
3.90228447	1.51348166	-2.05054183	o	8.000	0
4.62586060	3.91381180	-1.41257029	c	6.000	0
6.74103106	4.76362164	-1.69224975	o	8.000	0
0.45051071	0.77611052	-3.40226293	h	1.000	0
0.38739386	3.50241842	-0.44314078	c	6.000	0
2.39012561	5.18692029	-0.39174023	c	6.000	0
2.14030382	7.64356427	0.50474585	c	6.000	0
4.23487627	9.14136120	0.47810071	o	8.000	0
3.97125947	11.66943682	1.34151658	c	6.000	0
-0.25379022	8.37318753	1.36116973	c	6.000	0
-2.30074157	6.67791421	1.29521829	c	6.000	0
-1.98950367	4.22486072	0.38248057	c	6.000	0
-3.21820361	-8.57028596	2.24083725	h	1.000	0
-5.86608786	-8.58710453	-1.77709846	h	1.000	0
0.53025715	-9.24189463	-0.55520154	h	1.000	0
-7.59991159	-6.86953244	2.18206677	h	1.000	0
-6.29770131	-3.85693104	1.55486666	h	1.000	0
-8.67459885	-5.10244953	-0.44162900	h	1.000	0
-5.93147239	-4.61376636	-4.27531641	h	1.000	0
-3.02091620	-6.21908871	-4.55726354	h	1.000	0
0.69976559	-7.37371137	4.32747285	h	1.000	0
3.60351876	-3.95009454	5.43900976	h	1.000	0
5.28216249	-0.28780529	4.26832442	h	1.000	0
5.81298656	12.59313496	1.16690589	h	1.000	0
2.62615241	12.72125839	0.17272097	h	1.000	0
3.42758526	11.72216018	3.33725635	h	1.000	0
-0.60603517	10.25856729	2.08947019	h	1.000	0
-4.14927167	7.29131932	1.95416580	h	1.000	0
-3.57876335	2.92737475	0.30726947	h	1.000	0

Diastereomer **2b**/**3a**_1

atomic coordinates			atom	charge	isotop
-1.04955389	-2.59043658	-1.86175819	o	8.000	0
-0.34959933	-1.23474706	-3.61126664	c	6.000	0
-0.24377467	-2.22458560	-6.27634741	c	6.000	0
1.05767972	-0.73623730	-8.08273662	c	6.000	0
2.25255355	1.75839017	-7.39789987	c	6.000	0
4.75700760	1.29370651	-6.52390153	o	8.000	0
0.77573258	3.13751230	-5.33809838	c	6.000	0
-1.79278318	4.09711523	-6.26595391	c	6.000	0
0.51740702	1.43297933	-3.03206558	c	6.000	0
1.31109199	-1.59568475	-10.57396258	c	6.000	0
0.23791652	-3.87167090	-11.29243646	c	6.000	0
-1.10360006	-5.30483920	-9.54179417	c	6.000	0
-2.13765820	-7.46252850	-10.51764874	o	8.000	0
-1.37061836	-4.56727909	-6.99482128	c	6.000	0
-2.72460714	-6.26028474	-5.10018186	c	6.000	0
-4.72696095	-7.70309064	-6.31905522	o	8.000	0
-4.67915088	-10.20716674	-5.65254881	c	6.000	0
-6.25896193	-11.73992361	-6.29373289	o	8.000	0
-3.74789384	-5.16424358	-3.68383213	h	1.000	0
-1.16123671	-8.28626012	-3.93384289	c	6.000	0
-2.35989000	-10.59758416	-4.20879805	c	6.000	0
-1.37269706	-12.83917730	-3.25524224	c	6.000	0
-2.71856002	-14.99648865	-3.65737596	o	8.000	0
-1.71076907	-17.29231693	-2.69569433	c	6.000	0
0.92615478	-12.68478667	-1.96285854	c	6.000	0
2.15561060	-10.35040798	-1.65842366	c	6.000	0
1.11134794	-8.13545998	-2.63956946	c	6.000	0
2.39258226	2.97877530	-9.06709496	h	1.000	0
1.87063990	4.80009335	-4.74869280	h	1.000	0
5.50892963	0.00850377	-7.60369105	h	1.000	0
-2.73046529	5.19901454	-4.78554246	h	1.000	0
-1.55694536	5.33167331	-7.91001565	h	1.000	0
-3.06683654	2.55868919	-6.80169127	h	1.000	0
-0.82165292	2.26691547	-1.68865927	h	1.000	0
2.34533910	1.27084083	-2.06773834	h	1.000	0
2.33003232	-0.48811626	-11.97613937	h	1.000	0
0.41214927	-4.53118532	-13.23186239	h	1.000	0
-3.70216247	-7.82346619	-9.60774561	h	1.000	0
-3.02016031	-18.81430236	-3.19023566	h	1.000	0
0.11640713	-17.72619805	-3.56459041	h	1.000	0
-1.56223659	-17.23468029	-0.63268031	h	1.000	0
1.82320777	-14.34491108	-1.15726828	h	1.000	0
3.93648851	-10.26688209	-0.63286928	h	1.000	0
2.06717142	-6.34135399	-2.35818924	h	1.000	0

Diastereomer **2b**/**3a**_2

atomic coordinates			atom	charge	isotop
-0.99683054	-2.64410481	-1.92090662	o	8.000	0
-0.24698721	-1.33830405	-3.68761158	c	6.000	0
-0.20786987	-2.33891404	-6.35004673	c	6.000	0
1.09131684	-0.88269108	-8.18289211	c	6.000	0
2.33324486	1.60097598	-7.55115666	c	6.000	0
4.85187185	1.04218396	-6.78903011	o	8.000	0
0.95865807	2.99937332	-5.43428544	c	6.000	0
-1.62044016	4.01434523	-6.26954439	c	6.000	0
0.74757566	1.28803733	-3.12938648	c	6.000	0
1.29805288	-1.77294106	-10.66825992	c	6.000	0
0.16194953	-4.02681742	-11.36160043	c	6.000	0
-1.18996055	-5.41727791	-9.58639170	c	6.000	0
-2.29280472	-7.55304638	-10.53579011	o	8.000	0
-1.39650761	-4.66176540	-7.03904088	c	6.000	0
-2.74142570	-6.32245673	-5.11152022	c	6.000	0
-4.78610938	-7.74787715	-6.28050481	o	8.000	0
-4.76683417	-10.24363845	-5.58187305	c	6.000	0
-6.38614050	-11.75712011	-6.16806610	o	8.000	0
-3.72578405	-5.20279399	-3.68666671	h	1.000	0
-1.18542520	-8.36033739	-3.95633063	c	6.000	0
-2.42565247	-10.65503183	-4.17988524	c	6.000	0
-1.45301042	-12.90134929	-3.22254998	c	6.000	0
-2.84158119	-15.04127516	-3.56931472	o	8.000	0
-1.83379024	-17.34636310	-2.63030981	c	6.000	0
0.87645498	-12.76925743	-1.98288963	c	6.000	0
2.14994142	-10.45188627	-1.73420167	c	6.000	0
1.11890684	-8.23183601	-2.71685926	c	6.000	0
2.42716425	2.80586536	-9.23565853	h	1.000	0
2.10534389	4.63719896	-4.87606034	h	1.000	0
5.76347574	2.63446720	-6.68358339	h	1.000	0
-2.48914726	5.11926810	-4.75001561	h	1.000	0
-1.41446001	5.25948578	-7.90963771	h	1.000	0
-2.93909106	2.50464302	-6.77806970	h	1.000	0
-0.49794284	2.16184670	-1.72248537	h	1.000	0
2.60895590	1.05163259	-2.24820718	h	1.000	0
2.34949650	-0.70940319	-12.07950739	h	1.000	0
0.30670255	-4.70409527	-13.29649102	h	1.000	0
-3.83179768	-7.89092942	-9.57599821	h	1.000	0
-3.17322812	-18.85436456	-3.08573380	h	1.000	0
-0.03590480	-17.80310991	-3.54720493	h	1.000	0
-1.62913290	-17.28626981	-0.57220907	h	1.000	0
1.76538215	-14.43448410	-1.17900013	h	1.000	0
3.95689755	-10.38574586	-0.75456765	h	1.000	0
2.11252485	-6.45076913	-2.48574576	h	1.000	0

Diastereomer **2b**/**3a**_3

atomic coordinates			atom	charge	isotop
-1.09982061	-2.52202850	-1.91372566	o	8.000	0
-0.29423036	-1.29219473	-3.70972137	c	6.000	0
-0.22506638	-2.38540130	-6.33492892	c	6.000	0
1.09906472	-0.99739745	-8.20273423	c	6.000	0
2.34817369	1.50063152	-7.64432016	c	6.000	0
4.86132048	0.95450067	-6.85498155	o	8.000	0
0.97283101	2.97291715	-5.57884949	c	6.000	0
-1.59417297	3.97995221	-6.45908393	c	6.000	0
0.73737114	1.33414665	-3.22462867	c	6.000	0
1.32904439	-1.97306306	-10.65427594	c	6.000	0
0.19275207	-4.24545873	-11.28298783	c	6.000	0
-1.18164575	-5.56977881	-9.47527581	c	6.000	0
-2.28430095	-7.73408215	-10.35966764	o	8.000	0
-1.41200337	-4.72790581	-6.95683779	c	6.000	0
-2.77185029	-6.32718104	-4.98698727	c	6.000	0
-4.79952643	-7.80343510	-6.13216130	o	8.000	0
-4.73565369	-10.28804702	-5.40442777	c	6.000	0
-6.28371734	-11.86483450	-6.01726596	o	8.000	0
-3.77831843	-5.16858995	-3.60994383	h	1.000	0
-1.21396007	-8.31876341	-3.75337405	c	6.000	0
-2.41828253	-10.63008745	-3.96086598	c	6.000	0
-1.40973570	-12.81914620	-2.93814619	c	6.000	0
-2.61008974	-15.09551030	-3.05587613	o	8.000	0
-1.97665354	-16.38127996	-5.34301167	c	6.000	0
0.85510108	-12.64774804	-1.60059804	c	6.000	0
2.09192683	-10.31563702	-1.37364193	c	6.000	0
1.05862458	-8.13545998	-2.45267555	c	6.000	0
2.45456528	2.65052988	-9.36604964	h	1.000	0
2.12858752	4.61886862	-5.06446604	h	1.000	0
5.77802663	2.54602802	-6.78808525	h	1.000	0
-2.91962688	2.46495877	-6.93264929	h	1.000	0
-2.46514774	5.13400796	-4.97772761	h	1.000	0
-1.36986247	5.17595988	-8.13319231	h	1.000	0
-0.49869873	2.26615958	-1.84701832	h	1.000	0
2.59346015	1.09755294	-2.33229999	h	1.000	0
2.39995219	-0.96149266	-12.08895602	h	1.000	0
0.35564646	-4.98812110	-13.19236711	h	1.000	0
-3.86448994	-7.98919518	-9.44126074	h	1.000	0
0.03760555	-16.85881376	-5.38175106	h	1.000	0
-3.05474230	-18.14458342	-5.39743578	h	1.000	0
-2.47799788	-15.27106586	-7.01579724	h	1.000	0
1.65823468	-14.32242334	-0.72300922	h	1.000	0
3.85749796	-10.19715119	-0.32673365	h	1.000	0
2.01955032	-6.33473995	-2.23138862	h	1.000	0

Diastereomer **2b**/**3a**_4

atomic coordinates			atom	charge	isotop
-1.12967828	-2.47459637	-1.85325442	o	8.000	0
-0.38909461	-1.18769288	-3.63734486	c	6.000	0
-0.25700275	-2.27031698	-6.26482008	c	6.000	0
1.06712835	-0.84905395	-8.10900381	c	6.000	0
2.26597061	1.66333694	-7.49900022	c	6.000	0
4.76286575	1.21944027	-6.59344345	o	8.000	0
0.78196867	3.11615839	-5.49607949	c	6.000	0
-1.77539770	4.05686406	-6.47306790	c	6.000	0
0.49926564	1.48872625	-3.13789025	c	6.000	0
1.34151658	-1.79448394	-10.56678162	c	6.000	0
0.26777419	-4.09050119	-11.21798125	c	6.000	0
-1.09452938	-5.45790702	-9.43162313	c	6.000	0
-2.12972135	-7.64507605	-10.33906962	o	8.000	0
-1.38271261	-4.63341951	-6.91337409	c	6.000	0
-2.75049639	-6.26463111	-4.97394816	c	6.000	0
-4.74132287	-7.75392427	-6.16466459	o	8.000	0
-4.65458444	-10.24628407	-5.46622181	c	6.000	0
-6.16731021	-11.83724450	-6.12819288	o	8.000	0
-3.78946782	-5.12890570	-3.60144007	h	1.000	0
-1.18731493	-8.24884355	-3.73485473	c	6.000	0
-2.35705541	-10.57358464	-3.98788906	c	6.000	0
-1.33811508	-12.76018675	-2.97064948	c	6.000	0
-2.50766658	-15.04977893	-3.13165415	o	8.000	0
-1.81734962	-16.30701373	-5.41784483	c	6.000	0
0.89969861	-12.57367078	-1.59058249	c	6.000	0
2.10024163	-10.22776475	-1.31487144	c	6.000	0
1.05749074	-8.04928847	-2.38918075	c	6.000	0
2.42149507	2.82684132	-9.20712367	h	1.000	0
1.88084442	4.79177856	-4.95353911	h	1.000	0
5.51894517	-0.10147829	-7.62598981	h	1.000	0
-2.71893796	5.20940803	-5.03555323	h	1.000	0
-1.52179646	5.23756495	-8.15379032	h	1.000	0
-3.05247462	2.50785555	-6.96949895	h	1.000	0
-0.83922738	2.37670856	-1.82887695	h	1.000	0
2.32077267	1.34189453	-2.15901211	h	1.000	0
2.37784239	-0.73850497	-11.99579253	h	1.000	0
0.45825859	-4.81577808	-13.13208485	h	1.000	0
-3.73485473	-7.92362168	-9.47055149	h	1.000	0
0.20276761	-16.76092594	-5.42332503	h	1.000	0
-2.87370653	-18.08184451	-5.50836271	h	1.000	0
-2.30017465	-15.18697305	-7.08968554	h	1.000	0
1.70774551	-14.24664532	-0.71450545	h	1.000	0
3.84162426	-10.09718468	-0.22941275	h	1.000	0
1.98591319	-6.23798597	-2.12272937	h	1.000	0

Diastereomer **2b**/**3a**_5

atomic coordinates			atom	charge	isotop
-1.05711280	-2.72252844	-1.85306545	o	8.000	0
-0.36736276	-1.32904439	-3.57687363	c	6.000	0
-0.26588447	-2.26313602	-6.26311933	c	6.000	0
0.98511423	-0.71167086	-8.05212306	c	6.000	0
2.14672889	1.78786990	-7.32854692	c	6.000	0
4.67291478	1.34718576	-6.50594913	o	8.000	0
0.68256908	3.09385963	-5.21318749	c	6.000	0
-1.91523744	4.03437632	-6.07698130	c	6.000	0
0.48849421	1.33017823	-2.94608304	c	6.000	0
1.22208589	-1.51178091	-10.56470292	c	6.000	0
0.18292549	-3.79154652	-11.31983749	c	6.000	0
-1.10567876	-5.28991037	-9.58412403	c	6.000	0
-2.10855642	-7.44589891	-10.59512752	o	8.000	0
-1.35247699	-4.61395533	-7.01825389	c	6.000	0
-2.63711282	-6.37877057	-5.14118892	c	6.000	0
-4.60601848	-7.87052038	-6.36421967	o	8.000	0
-4.47298176	-10.37667517	-5.72587019	c	6.000	0
-6.01915568	-11.96045465	-6.32377953	o	8.000	0
-3.68458802	-5.33734249	-3.70178452	h	1.000	0
-0.99966512	-8.37148677	-4.01717982	c	6.000	0
-2.13293389	-10.70888903	-4.31670141	c	6.000	0
-1.04766417	-12.90834128	-3.40056218	c	6.000	0
-2.07605313	-15.23875154	-3.78455453	o	8.000	0
-3.87412755	-15.81171651	-1.85552209	c	6.000	0
1.28369096	-12.73939976	-2.18225574	c	6.000	0
2.43963644	-10.37950976	-1.84418373	c	6.000	0
1.29313959	-8.17835676	-2.75351995	c	6.000	0
2.23838061	3.04850620	-8.97090790	h	1.000	0
1.76103578	4.75889732	-4.60110519	h	1.000	0
5.43145085	0.10525775	-7.63090310	h	1.000	0
-2.84725037	5.08279638	-4.55480690	h	1.000	0
-1.72475304	5.31390989	-7.69250817	h	1.000	0
-3.17247223	2.48933624	-6.63274976	h	1.000	0
-0.83129053	2.11781608	-1.55618947	h	1.000	0
2.33626842	1.16123671	-2.02125107	h	1.000	0
2.20247581	-0.35507954	-11.95459650	h	1.000	0
0.34487502	-4.40495162	-13.27532609	h	1.000	0
-3.64169123	-7.87241010	-9.66179178	h	1.000	0
-4.70428424	-17.65438846	-2.29053705	h	1.000	0
-2.94986250	-15.94399734	-0.00755890	h	1.000	0
-5.40310496	-14.41879937	-1.78919270	h	1.000	0
2.20965677	-14.44072020	-1.49798591	h	1.000	0
4.24659257	-10.26177983	-0.87021888	h	1.000	0
2.20039711	-6.36081817	-2.45777781	h	1.000	0

Diastereomer **2b**/**3a**_6

atomic coordinates			atom	charge	isotop
-0.99739745	-2.77922023	-1.91296977	o	8.000	0
-0.25908145	-1.43373522	-3.65473034	c	6.000	0
-0.22790097	-2.37633061	-6.33908632	c	6.000	0
1.01856239	-0.85377827	-8.15473519	c	6.000	0
2.22647533	1.63650283	-7.48388241	c	6.000	0
4.76853493	1.10473390	-6.78222709	o	8.000	0
0.86946299	2.95798832	-5.30748482	c	6.000	0
-1.73930393	3.95330707	-6.07017829	c	6.000	0
0.72697764	1.18410240	-3.04529366	c	6.000	0
1.20753500	-1.68317907	-10.66259074	c	6.000	0
0.10544672	-3.94215769	-11.39278092	c	6.000	0
-1.19222822	-5.40083729	-9.63193410	c	6.000	0
-2.26464780	-7.53603885	-10.61648142	o	8.000	0
-1.37685446	-4.70844164	-7.06530807	c	6.000	0
-2.65204166	-6.44302125	-5.15592878	c	6.000	0
-4.66573382	-7.91644072	-6.32831488	o	8.000	0
-4.56331067	-10.41598148	-5.65878491	c	6.000	0
-6.15011370	-11.97972985	-6.20094734	o	8.000	0
-3.65926569	-5.37797160	-3.70556398	h	1.000	0
-1.02234184	-8.45028835	-4.04665954	c	6.000	0
-2.20058608	-10.77143896	-4.29364675	c	6.000	0
-1.13175698	-12.97807217	-3.37486190	c	6.000	0
-2.20738910	-15.29544333	-3.70556398	o	8.000	0
-3.97560584	-15.81001575	-1.73325681	c	6.000	0
1.22983377	-12.83426401	-2.21286930	c	6.000	0
2.43358932	-10.49081463	-1.93224497	c	6.000	0
1.30315514	-8.28115786	-2.84082530	c	6.000	0
2.26729342	2.88353311	-9.13947147	h	1.000	0
2.00121998	4.59864855	-4.72696095	h	1.000	0
5.65651724	2.70948933	-6.66393024	h	1.000	0
-2.59912932	5.00172713	-4.50624094	h	1.000	0
-1.58170077	5.24625769	-7.67852420	h	1.000	0
-3.04245908	2.43699082	-6.59835674	h	1.000	0
-0.49510825	2.00858991	-1.58888173	h	1.000	0
2.60876693	0.94051670	-2.21060163	h	1.000	0
2.21967232	-0.56805168	-12.06268883	h	1.000	0
0.23697166	-4.57200341	-13.34467904	h	1.000	0
-3.77094850	-7.94157408	-9.63269000	h	1.000	0
-4.84506883	-17.64437291	-2.12499704	h	1.000	0
-3.01675880	-15.93209206	0.09769884	h	1.000	0
-5.47944990	-14.39083143	-1.65634496	h	1.000	0
2.14351635	-14.54295438	-1.53086714	h	1.000	0
4.26719058	-10.39235990	-1.00741300	h	1.000	0
2.25066383	-6.47665838	-2.59686165	h	1.000	0

Diastereomer **2b**/**3a**_7

atomic coordinates			atom	charge	isotop
0.11036001	-2.58287768	-1.72872147	o	8.000	0
0.17290994	-1.20129890	-3.59482602	c	6.000	0
-0.04081808	-2.28524581	-6.20189220	c	6.000	0
0.93276882	-0.82184190	-8.21331670	c	6.000	0
1.88519079	1.82963284	-7.80305715	c	6.000	0
4.49565847	1.64425071	-7.18832924	o	8.000	0
0.45466811	3.16434641	-5.67333580	c	6.000	0
-2.28713554	3.76131090	-6.38708536	c	6.000	0
0.61756250	1.60740105	-3.25146279	c	6.000	0
1.05408924	-1.85457723	-10.64860676	c	6.000	0
0.18651597	-4.28873346	-11.10195206	c	6.000	0
-0.86360484	-5.68713080	-9.13304641	c	6.000	0
-1.67165174	-8.09048450	-9.63174513	o	8.000	0
-1.07241958	-4.71902410	-6.66052873	c	6.000	0
-2.39787349	-6.23685213	-4.60960896	c	6.000	0
-4.78308582	-7.23935185	-5.52820483	o	8.000	0
-4.93823233	-9.80465507	-5.23151783	c	6.000	0
-6.75350326	-11.08456658	-5.81676601	o	8.000	0
-2.97839736	-5.07013522	-3.01260140	h	1.000	0
-1.04823109	-8.52720021	-3.71463466	c	6.000	0
-2.56265761	-10.63065437	-4.07538338	c	6.000	0
-1.79864133	-13.04855895	-3.38941279	c	6.000	0
-3.43438828	-14.98722900	-3.83349844	o	8.000	0
-2.66867125	-17.45540030	-3.10727668	c	6.000	0
0.59715346	-13.28666445	-2.29242677	c	6.000	0
2.13765820	-11.15448645	-1.90522189	c	6.000	0
1.31562733	-8.75245556	-2.61235741	c	6.000	0
1.71681619	2.93039832	-9.55143177	h	1.000	0
1.38894871	4.97923939	-5.29425674	h	1.000	0
5.17369221	3.35086238	-7.12407855	h	1.000	0
-3.20119607	4.85848589	-4.88853254	h	1.000	0
-2.35818924	4.89023329	-8.12034217	h	1.000	0
-3.41643588	2.05564409	-6.68944154	h	1.000	0
-0.74152853	2.31056814	-1.85533312	h	1.000	0
2.51333576	1.80865688	-2.43566801	h	1.000	0
1.86402586	-0.77252004	-12.19856014	h	1.000	0
0.34298529	-5.06956830	-12.99394587	h	1.000	0
-1.66447078	-8.39435246	-11.44191380	h	1.000	0
-2.36140178	-17.57086257	-1.06429376	h	1.000	0
-4.19934941	-18.76006722	-3.58764507	h	1.000	0
-0.98398040	-18.05066403	-4.15097243	h	1.000	0
1.33357973	-15.10854941	-1.70396605	h	1.000	0
3.98770009	-11.38087564	-1.03727067	h	1.000	0
2.49765103	-7.10366951	-2.29280472	h	1.000	0

Diastereomer **2b**/**3a**_8

atomic coordinates			atom	charge	isotop
0.05272336	-2.50672172	-1.70869037	o	8.000	0
0.13776104	-1.17937808	-3.61258945	c	6.000	0
-0.05102261	-2.33778020	-6.18979795	c	6.000	0
0.93522546	-0.92898937	-8.23391471	c	6.000	0
1.88122237	1.73457962	-7.89149634	c	6.000	0
4.48772162	1.56922858	-7.25598144	o	8.000	0
0.43595982	3.12636292	-5.80901814	c	6.000	0
-2.30168643	3.70046172	-6.55678277	c	6.000	0
0.58430332	1.63706975	-3.34424834	c	6.000	0
1.07789979	-2.02937690	-10.63802430	c	6.000	0
0.21958618	-4.47713916	-11.02957555	c	6.000	0
-0.84262888	-5.82394697	-9.03081222	c	6.000	0
-1.64047126	-8.24298540	-9.47357505	o	8.000	0
-1.07260855	-4.78705424	-6.58777428	c	6.000	0
-2.40751109	-6.24932432	-4.50321738	c	6.000	0
-4.77949534	-7.29755541	-5.41519921	o	8.000	0
-4.91177617	-9.85341001	-5.05388357	c	6.000	0
-6.68566209	-11.18698974	-5.64612374	o	8.000	0
-3.01335729	-5.03989960	-2.94797277	h	1.000	0
-1.05049876	-8.50414555	-3.52981945	c	6.000	0
-2.54583905	-10.62290649	-3.85768693	c	6.000	0
-1.74497311	-12.99526868	-3.09574935	c	6.000	0
-3.20062915	-15.11081708	-3.31098916	o	8.000	0
-2.97291715	-16.16792988	-5.78293992	c	6.000	0
0.60093291	-13.20257163	-1.90389908	c	6.000	0
2.12102861	-11.05716555	-1.55751228	c	6.000	0
1.29729699	-8.68499234	-2.37444089	c	6.000	0
1.72248537	2.78602324	-9.67086246	h	1.000	0
1.36608302	4.95202733	-5.47415866	h	1.000	0
5.16254283	3.27829690	-7.22725760	h	1.000	0
-3.42739629	1.98591319	-6.81983264	h	1.000	0
-3.22670737	4.83713198	-5.09470166	h	1.000	0
-2.36253561	4.78157404	-8.32065314	h	1.000	0
-0.78159073	2.38011007	-1.97570867	h	1.000	0
2.47591918	1.85892360	-2.52391822	h	1.000	0
1.89841887	-0.98889369	-12.21103233	h	1.000	0
0.39438584	-5.30899660	-12.89813675	h	1.000	0
-1.63744770	-8.57538822	-11.27920838	h	1.000	0
-1.04029424	-16.83122376	-6.11817733	h	1.000	0
-4.23771086	-17.79989737	-5.89216609	h	1.000	0
-3.52887458	-14.82754714	-7.25749322	h	1.000	0
1.24646336	-15.03201550	-1.22869993	h	1.000	0
3.94310255	-11.23914618	-0.62379860	h	1.000	0
2.46023445	-7.01806492	-2.07756491	h	1.000	0

Diastereomer **2b**/**3a**_9

atomic coordinates			atom	charge	isotop
-1.77955510	-2.63333337	-2.01803854	o	8.000	0
-1.00042102	-1.33490254	-3.77094850	c	6.000	0
-0.56332736	-2.40278678	-6.36289687	c	6.000	0
0.85774669	-0.90536779	-8.07536669	c	6.000	0
1.85552209	1.70283222	-7.41509638	c	6.000	0
4.32104778	1.98818087	-8.46446130	o	8.000	0
1.99989717	2.17167327	-4.56387759	c	6.000	0
2.64183714	4.92424836	-3.95009454	c	6.000	0
-0.50776941	1.44299488	-3.37146040	c	6.000	0
1.29351754	-1.81829449	-10.52690840	c	6.000	0
0.37019735	-4.15399599	-11.26975974	c	6.000	0
-1.03689273	-5.60492771	-9.59130499	c	6.000	0
-1.90257627	-7.82875743	-10.58265532	o	8.000	0
-1.53332379	-4.81294349	-7.09818930	c	6.000	0
-2.96668106	-6.50859475	-5.26609982	c	6.000	0
-4.79234548	-8.08651607	-6.58966400	o	8.000	0
-4.68746568	-10.56092347	-5.82564773	c	6.000	0
-6.14142096	-12.18816665	-6.52919277	o	8.000	0
-4.15418496	-5.41822277	-3.98014118	h	1.000	0
-1.41672768	-8.41495047	-3.89850501	c	6.000	0
-2.48158836	-10.78958033	-4.18309777	c	6.000	0
-1.47625406	-12.94651374	-3.06759243	c	6.000	0
-2.68530084	-15.17884723	-3.49467054	o	8.000	0
-1.64236098	-17.39303934	-2.38955870	c	6.000	0
0.69787586	-12.63697660	-1.59908625	c	6.000	0
1.79089346	-10.23664647	-1.28520274	c	6.000	0
0.73170196	-8.10975970	-2.43094370	c	6.000	0
0.60660209	3.09121401	-8.31933033	h	1.000	0
3.51016629	0.99172828	-3.76112192	h	1.000	0
4.71222109	3.78190891	-8.50301171	h	1.000	0
4.50869758	5.44014359	-4.67499348	h	1.000	0
1.25194356	6.22022254	-4.77023568	h	1.000	0
2.66829330	5.23000605	-1.90295422	h	1.000	0
-2.09532834	2.52146158	-4.15021654	h	1.000	0
-0.44465256	1.79712955	-1.33036720	h	1.000	0
2.37859828	-0.70524579	-11.87560594	h	1.000	0
0.72546586	-4.84336808	-13.17309191	h	1.000	0
-3.50959938	-8.24166259	-9.77649815	h	1.000	0
-1.65917955	-17.27323070	-0.32389906	h	1.000	0
-2.83137667	-18.99363737	-2.93682338	h	1.000	0
0.26664036	-17.75416600	-3.10160750	h	1.000	0
1.59984214	-14.22340169	-0.66178209	h	1.000	0
3.47709609	-10.03293399	-0.12566679	h	1.000	0
1.58113386	-6.26349727	-2.14672889	h	1.000	0

Diastereomer **2b**/**3a**_10

atomic coordinates			atom	charge	isotop
0.04667624	-2.49141493	-1.67939962	o	8.000	0
0.04837699	-1.08583664	-3.52811869	c	6.000	0
-0.09845473	-2.16562615	-6.14066507	c	6.000	0
0.87834471	-0.67501018	-8.13073566	c	6.000	0
1.78522428	1.98666908	-7.66945351	c	6.000	0
4.37452703	1.91637127	-6.93775156	o	8.000	0
0.24736515	3.29190293	-5.59680189	c	6.000	0
-2.47913172	3.82915207	-6.41014002	c	6.000	0
0.35318981	1.74005982	-3.16623614	c	6.000	0
1.04161705	-1.67996653	-10.57434053	c	6.000	0
0.23035762	-4.12905160	-11.05074049	c	6.000	0
-0.80672409	-5.56467655	-9.10073209	c	6.000	0
-1.55146516	-7.98258113	-9.62286342	o	8.000	0
-1.06731732	-4.61773478	-6.62462394	c	6.000	0
-2.39522787	-6.17014480	-4.59978238	c	6.000	0
-4.74642513	-7.20741547	-5.56467655	o	8.000	0
-4.85678514	-9.77857685	-5.29973694	c	6.000	0
-6.63350565	-11.08664528	-5.93808643	o	8.000	0
-3.01808161	-5.02194720	-3.00466455	h	1.000	0
-1.02064108	-8.44254048	-3.69781610	c	6.000	0
-2.48820240	-10.57075005	-4.10656386	c	6.000	0
-1.69319462	-12.98033984	-3.42588451	c	6.000	0
-3.28283224	-14.94452118	-3.92137070	o	8.000	0
-2.49746206	-17.40173208	-3.17870833	c	6.000	0
0.68351394	-13.18367437	-2.28222225	c	6.000	0
2.17488581	-11.02674096	-1.84361682	c	6.000	0
1.32299727	-8.63396973	-2.54697288	c	6.000	0
1.66314797	3.10066264	-9.41253690	h	1.000	0
1.12911137	5.12644906	-5.18673132	h	1.000	0
5.19069974	0.52723359	-7.82346619	h	1.000	0
-3.46726951	4.91461076	-4.95051555	h	1.000	0
-2.51257987	4.94786994	-8.15095573	h	1.000	0
-3.56232274	2.09910779	-6.74122004	h	1.000	0
-1.09830883	2.38275568	-1.83586894	h	1.000	0
2.19869636	2.01992826	-2.26275807	h	1.000	0
1.81848346	-0.56313839	-12.11767986	h	1.000	0
0.41177132	-4.89401274	-12.94764758	h	1.000	0
-1.61193639	-8.24865457	-11.43851229	h	1.000	0
-3.99677077	-18.72869777	-3.69554843	h	1.000	0
-0.78026792	-17.97035067	-4.18347572	h	1.000	0
-2.23592396	-17.51473770	-1.12930034	h	1.000	0
1.44148309	-14.99705557	-1.69432845	h	1.000	0
4.00905399	-11.22705193	-0.93579238	h	1.000	0
2.46495877	-6.96685334	-2.18206677	h	1.000	0

Diastereomer **2b**/**3a**_11

atomic coordinates			atom	charge	isotop
0.13549336	-2.70835550	-1.71983975	o	8.000	0
0.16742974	-1.28936014	-3.55854328	c	6.000	0
-0.05895946	-2.32228445	-6.18564055	c	6.000	0
0.86511662	-0.80105491	-8.17760087	c	6.000	0
1.78276763	1.85552209	-7.72387763	c	6.000	0
4.40400676	1.69697407	-7.15015677	o	8.000	0
0.36415023	3.12277244	-5.54596826	c	6.000	0
-2.39617274	3.69403665	-6.20756138	c	6.000	0
0.58524818	1.51650522	-3.16132285	c	6.000	0
0.97169718	-1.78012202	-10.63575663	c	6.000	0
0.13813898	-4.21805770	-11.13048693	c	6.000	0
-0.86247101	-5.67560347	-9.17896675	c	6.000	0
-1.63858153	-8.08084689	-9.71753870	o	8.000	0
-1.05522307	-4.76343267	-6.68358339	c	6.000	0
-2.31585938	-6.35325926	-4.64702554	c	6.000	0
-4.69237896	-7.39752192	-5.55163744	o	8.000	0
-4.77684972	-9.96925022	-5.30483920	c	6.000	0
-6.57076674	-11.29659386	-5.84908033	o	8.000	0
-2.90299729	-5.23454139	-3.01864853	h	1.000	0
-0.89743094	-8.62886747	-3.81951446	c	6.000	0
-2.36933863	-10.75443143	-4.20634140	c	6.000	0
-1.51083604	-13.14701369	-3.58310972	c	6.000	0
-2.86879324	-15.28996312	-4.03588810	o	8.000	0
-4.58031820	-15.77259918	-2.00651121	c	6.000	0
0.92218635	-13.38493021	-2.58760200	c	6.000	0
2.41223541	-11.23177625	-2.16524820	c	6.000	0
1.49930871	-8.82955639	-2.77279516	c	6.000	0
1.57244112	2.99049161	-9.44579608	h	1.000	0
1.27707692	4.94276768	-5.14081097	h	1.000	0
5.05936378	3.41190053	-7.06681985	h	1.000	0
-3.30456409	4.74396849	-4.67215889	h	1.000	0
-2.50917836	4.85943075	-7.91398408	h	1.000	0
-3.50449712	1.97854326	-6.53164941	h	1.000	0
-0.75966991	2.17318505	-1.72928839	h	1.000	0
2.49122596	1.72305229	-2.37122835	h	1.000	0
1.74383928	-0.65233346	-12.17248192	h	1.000	0
0.28062433	-4.95429501	-13.04137800	h	1.000	0
-1.56393735	-8.37904568	-11.52732942	h	1.000	0
-5.69279998	-17.44368400	-2.50067459	h	1.000	0
-3.54607109	-16.18399256	-0.26059323	h	1.000	0
-5.88838663	-14.19864628	-1.70302119	h	1.000	0
1.66598256	-15.24612148	-2.13841409	h	1.000	0
4.29610339	-11.43643359	-1.36872864	h	1.000	0
2.64920707	-7.16073924	-2.43831363	h	1.000	0

Diastereomer **2b**/**3a**_12

atomic coordinates			atom	charge	isotop
-1.77275209	-2.55453179	-1.99857436	o	8.000	0
-1.09906472	-1.22189692	-3.76849186	c	6.000	0
-0.64874298	-2.28108842	-6.36081817	c	6.000	0
0.72773353	-0.75022128	-8.08009100	c	6.000	0
1.68752544	1.87082887	-7.41585227	c	6.000	0
4.17516092	2.23781369	-8.39302965	o	8.000	0
1.77331900	2.36914965	-4.56671218	c	6.000	0
2.33532356	5.14723604	-3.99412516	c	6.000	0
-0.72508792	1.57848824	-3.39602684	c	6.000	0
1.15178808	-1.63272338	-10.54429388	c	6.000	0
0.28497070	-3.99110159	-11.28166502	c	6.000	0
-1.06259301	-5.48530805	-9.58998218	c	6.000	0
-1.87196271	-7.73200345	-10.57566334	o	8.000	0
-1.56355940	-4.71108725	-7.09214218	c	6.000	0
-2.95572065	-6.43810797	-5.25684016	c	6.000	0
-4.76248780	-8.03700525	-6.57927051	o	8.000	0
-4.60488464	-10.51651491	-5.84171040	c	6.000	0
-6.02784842	-12.16530096	-6.55791660	o	8.000	0
-4.14946064	-5.37230243	-3.95519680	h	1.000	0
-1.36570508	-8.32632232	-3.91003234	c	6.000	0
-2.38729102	-10.71814869	-4.21144366	c	6.000	0
-1.33868199	-12.86544449	-3.11748120	c	6.000	0
-2.50653274	-15.11610832	-3.56099993	o	8.000	0
-1.42617631	-17.31858413	-2.46836028	c	6.000	0
0.83318025	-12.52756146	-1.65199859	c	6.000	0
1.88065545	-10.10965687	-1.31827295	c	6.000	0
0.77913409	-7.99316360	-2.44322692	c	6.000	0
0.44616434	3.25221868	-8.33822759	h	1.000	0
3.30796560	1.24495158	-3.73050836	h	1.000	0
5.12134680	0.68181319	-8.13772765	h	1.000	0
4.16382256	5.71585464	-4.77722767	h	1.000	0
0.88250210	6.38727433	-4.79140061	h	1.000	0
2.40051911	5.47359175	-1.95133121	h	1.000	0
-2.33891404	2.58665713	-4.21371133	h	1.000	0
-0.70505682	1.96323648	-1.36003590	h	1.000	0
2.15995697	-0.47054181	-11.91321149	h	1.000	0
0.62398757	-4.66252129	-13.19482376	h	1.000	0
-3.48219835	-8.16985299	-9.78745857	h	1.000	0
-1.44393974	-17.21049179	-0.40194475	h	1.000	0
-2.58873583	-18.93581175	-3.02393976	h	1.000	0
0.48811626	-17.64361702	-3.18324367	h	1.000	0
1.76651599	-14.10491586	-0.72962326	h	1.000	0
3.56307863	-9.88459049	-0.15684727	h	1.000	0
1.59039351	-6.13329514	-2.13860307	h	1.000	0

Diastereomer **2b**/**3a**_13

atomic coordinates			atom	charge	isotop
0.06689631	-2.61500302	-1.66768331	o	8.000	0
0.03798350	-1.17389787	-3.48881239	c	6.000	0
-0.11829686	-2.20379862	-6.12157884	c	6.000	0
0.80993662	-0.65705778	-8.09199628	c	6.000	0
1.68072242	2.00896785	-7.58762837	c	6.000	0
4.27966278	1.96437032	-6.88975251	o	8.000	0
0.15004426	3.24730539	-5.46905640	c	6.000	0
-2.59478295	3.75809836	-6.23609624	c	6.000	0
0.31218276	1.64765222	-3.07307264	c	6.000	0
0.96073677	-1.60910180	-10.55752196	c	6.000	0
0.18481522	-4.06121043	-11.07511795	c	6.000	0
-0.80388950	-5.55428305	-9.14325093	c	6.000	0
-1.51612728	-7.97313250	-9.70506651	o	8.000	0
-1.05049876	-4.66252129	-6.64503298	c	6.000	0
-2.31415862	-6.28522912	-4.63455334	c	6.000	0
-4.65477341	-7.36520761	-5.58735326	o	8.000	0
-4.69313485	-9.94071535	-5.37022373	c	6.000	0
-6.44793454	-11.29697180	-5.96794410	o	8.000	0
-2.94494921	-5.18465262	-3.00957784	h	1.000	0
-0.86965197	-8.54061726	-3.79683775	c	6.000	0
-2.29242677	-10.68999177	-4.23204168	c	6.000	0
-1.40142090	-13.07180259	-3.61296740	c	6.000	0
-2.70816652	-15.23516106	-4.11487866	o	8.000	0
-4.44936018	-15.77335507	-2.12480806	c	6.000	0
1.01402704	-13.27268047	-2.56775987	c	6.000	0
2.45229760	-11.09552700	-2.09287169	c	6.000	0
1.50686762	-8.70540138	-2.69890686	c	6.000	0
1.51877289	3.15622059	-9.30557840	h	1.000	0
1.00873581	5.08657583	-5.03252967	h	1.000	0
5.10887460	0.61529483	-7.82441106	h	1.000	0
-3.58065308	4.79706979	-4.74151184	h	1.000	0
-2.66829330	4.91196514	-7.95253449	h	1.000	0
-3.65510829	2.01822751	-6.58985297	h	1.000	0
-1.12816650	2.24386081	-1.70944626	h	1.000	0
2.16675999	1.93318984	-2.19019259	h	1.000	0
1.70018660	-0.44805407	-12.08649938	h	1.000	0
0.35413468	-4.78214095	-12.99035539	h	1.000	0
-1.50195433	-8.23561546	-11.52222716	h	1.000	0
-5.79371135	-14.22850395	-1.83114462	h	1.000	0
-5.51894517	-17.46031359	-2.65865570	h	1.000	0
-3.44175821	-16.18342564	-0.36320536	h	1.000	0
1.78295661	-15.12366722	-2.11819402	h	1.000	0
4.32048086	-11.27221639	-1.25364432	h	1.000	0
2.61538097	-7.01882081	-2.31850499	h	1.000	0

Diastereomer **2b**/**3a**_14

atomic coordinates			atom	charge	isotop
-0.00396842	-2.41639281	-1.66295900	o	8.000	0
0.01587370	-1.06372684	-3.55041746	c	6.000	0
-0.10771439	-2.21778259	-6.13253925	c	6.000	0
0.88099032	-0.78177970	-8.15624697	c	6.000	0
1.78106688	1.89350559	-7.76375085	c	6.000	0
4.36507840	1.84758524	-7.01239574	o	8.000	0
0.22733405	3.25429738	-5.73928724	c	6.000	0
-2.49424952	3.76433446	-6.58626249	c	6.000	0
0.31841885	1.77124031	-3.26582470	c	6.000	0
1.06372684	-1.85438826	-10.56942724	c	6.000	0
0.25945940	-4.31689038	-10.98308829	c	6.000	0
-0.78971655	-5.70073683	-9.00152147	c	6.000	0
-1.52784358	-8.13357025	-9.46733896	o	8.000	0
-1.06845116	-4.68557595	-6.55432612	c	6.000	0
-2.40429856	-6.18261699	-4.49376875	c	6.000	0
-4.74359054	-7.26448520	-5.44770250	o	8.000	0
-4.83335253	-9.82619795	-5.11567762	c	6.000	0
-6.56944393	-11.18680077	-5.75629478	o	8.000	0
-3.04869517	-4.99208953	-2.93890208	h	1.000	0
-1.02271978	-8.42061965	-3.51659136	c	6.000	0
-2.47365151	-10.56338012	-3.88716666	c	6.000	0
-1.64387276	-12.92742751	-3.13089826	c	6.000	0
-3.05871072	-15.06565263	-3.38638923	o	8.000	0
-2.76863776	-16.10292330	-5.86022971	c	6.000	0
0.68426983	-13.10279409	-1.89955271	c	6.000	0
2.15825622	-10.93319952	-1.50743454	c	6.000	0
1.30655665	-8.56915213	-2.31926088	c	6.000	0
1.66957304	2.95779934	-9.53820369	h	1.000	0
1.10378903	5.10074878	-5.37494804	h	1.000	0
5.19088872	0.43747160	-7.85464668	h	1.000	0
-3.49391465	4.88872151	-5.16443255	h	1.000	0
-2.51692624	4.83391945	-8.35788075	h	1.000	0
-3.57271623	2.02408566	-6.87652443	h	1.000	0
-1.14177253	2.45040788	-1.96323648	h	1.000	0
2.15806724	2.07680902	-2.35856719	h	1.000	0
1.85060880	-0.77951203	-12.13733301	h	1.000	0
0.45712475	-5.13268515	-12.85731867	h	1.000	0
-1.59303913	-8.42855650	-11.27883043	h	1.000	0
-0.81957422	-16.73239108	-6.16390870	h	1.000	0
-4.00451865	-17.75416600	-6.00309301	h	1.000	0
-3.31892601	-14.76159570	-7.33610582	h	1.000	0
1.34983138	-14.92581290	-1.22643226	h	1.000	0
3.96464543	-11.09061371	-0.53894989	h	1.000	0
2.43113267	-6.88408333	-1.98099991	h	1.000	0

Table S6. ECD calculation of donghaecyclinone B (**2c**).

total energy = -1222.730359

kinetic energy = 1211.487262306

potential energy = -2434.217620952

Parameters of Level DFT

DFT settings (Functional B3LYP / Gridsize M3)

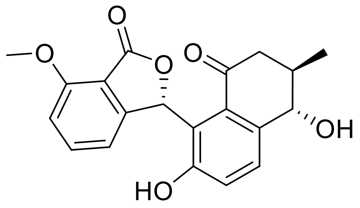

2c

Geometry optimization options (Energy 10-6 Hartree, Gradient norm ｜dE / dxyz｜= 10-3 Hartree/Bohr)

Energy minimized coordinates of donghaecyclinone B at the basis set def-SVP all atoms (Å).

Atom	X	Y	Z	Atom	X	Y	Z
C	4.1323	-1.1919	-0.3498	H	7.7648	0.1202	2.4882
C	3.3464	-2.274	-2.3756	H	7.272	1.7848	2.1046
C	4.6697	-2.6022	-2.6974	H	8.4133	0.9441	1.0381
C	5.7209	-2.2157	-1.8732	H	1.6212	-1.0762	-0.9646
C	5.4699	-1.5125	-0.6932	O	1.0828	-0.466	-2.1483
H	4.8702	-3.1648	-3.6154	C	0.6258	-2.1639	-0.6167
H	6.7506	-2.4538	-2.139	C	1.6169	-0.3204	-0.1708
O	2.3159	-2.6659	-3.1609	C	-0.3943	-2.1268	-1.562
H	2.6495	-3.1124	-3.9502	O	-0.0926	-1.0566	-2.548
C	3.8944	-0.5504	0.9821	C	-0.6903	-0.6869	-3.5189
C	5.0654	0.1523	1.6609	C	-1.4859	-3.0152	-1.4859
C	6.343	0.2768	0.8311	C	-1.5038	-3.93	-0.4177
C	6.6278	-1.0988	0.2011	C	-0.4664	-3.9421	0.5275
O	2.8218	-0.6077	1.5601	O	0.6158	-3.0614	0.4499
O	7.8157	-1.1159	-0.5692	H	-2.326	-4.6375	-0.3095
H	8.5686	-1.1167	0.034	H	1.4112	-3.0625	1.1966
H	6.7141	-1.8356	1.0315	H	-0.5177	-4.6625	1.349
C	3.0559	-1.5347	-1.209	C	-2.4352	-2.9267	-2.4359
C	7.5147	0.8066	1.6598	H	-3.5511	-3.7889	-2.3919
H	6.1643	0.9745	-0.0066	H	-4.1388	-3.6521	-1.4663
H	5.2675	-0.4184	2.5874	H	-3.2532	-4.8501	-2.4728
H	4.6934	1.1337	1.9987	H	-4.1774	-3.527	-3.2547

Table S7. ECD calculation of *ent*-donghaecyclinone B (**2d**).

total energy = -1222.730359

kinetic energy = 1211.487262306

potential energy = -2434.217620952

Parameters of Level DFT

DFT settings (Functional B3LYP / Gridsize M3)

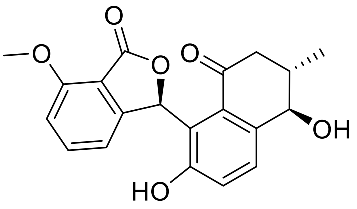

2d

Geometry optimization options (Energy 10-6 Hartree, Gradient norm ｜dE / dxyz｜= 10-3 Hartree/Bohr)

Energy minimized coordinates of *ent*.- donghaecyclinone B at the basis set def-SVP for all atoms (Å).

Atom	X	Y	Z	Atom	X	Y	Z
C	0.6169	2.6457	-0.8909	H	3.3379	4.2192	0.641
C	-0.191	1.3569	-0.7784	H	1.9914	4.9718	-0.2347
C	0.2405	0.3399	0.2323	H	4.2778	2.1331	0.7968
O	-1.1274	1.1754	-1.5387	H	-2.4036	0.2637	-0.0974
C	1.7084	2.8444	0.1607	O	-2.8964	-0.8747	1.5394
H	-0.1092	3.4758	-0.8968	C	-2.6022	-1.8107	-0.5764
H	1.0617	2.6392	-1.904	C	-3.5872	-2.5265	0.0973
C	2.5029	1.5301	0.2652	C	-3.7586	-1.9418	1.4527
H	1.2364	3.0041	1.147	O	-4.4829	-2.2468	2.3578
C	2.5933	4.0525	-0.1532	C	-4.2471	-3.609	-0.5194
C	1.5814	0.3971	0.6882	C	-3.8733	-3.93	-1.8371
O	3.5724	1.6053	1.1899	C	-2.8805	-3.1889	-2.4971
H	2.9043	1.2998	-0.7479	C	-2.2275	-2.1157	-1.8842
C	-0.6348	-0.6893	0.6665	O	-5.1891	-4.2581	0.1903
C	-0.1106	-1.6998	1.5006	H	-4.3536	-4.7561	-2.3614
C	1.2214	-1.6454	1.9311	H	-2.621	-3.463	-3.5238
C	2.0549	-0.6027	1.5406	H	-1.4702	-1.5303	-2.4076
C	-2.1248	-0.7022	0.3394	C	-5.8939	-5.3283	-0.3994
O	-0.923	-2.7215	1.8581	H	-6.4543	-5.0059	-1.2959
H	1.6003	-2.4343	2.5896	H	-6.6057	-5.684	0.3575
H	3.0838	-0.5523	1.8968	H	-5.2209	-6.1594	-0.6783
H	3.1274	3.9229	-1.1116	H	-0.4704	-3.3077	2.4784

Table S8. ECD calculation of donghaecyclinone C (**3c**).

total energy = -1222.729104885

kinetic energy = 1211.475176285

potential energy = -2434.204281170

Parameters of Level DFT

DFT settings (Functional B3LYP / Gridsize M3)

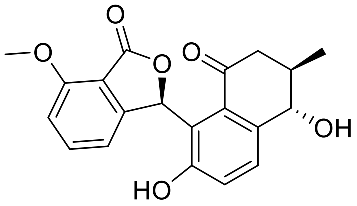

3c

Geometry optimization options (Energy 10-6 Hartree, Gradient norm │dE / dxyz│= 10-3 Hartree/Bohr)

Energy minimized coordinates of donghaecyclinone C at the basis set def-SVP for all atoms (Å).

Atom	X	Y	Z	Atom	X	Y	Z
C	1.6812	0.975	-1.5572	H	5.3866	-4.841	-1.2105
C	0.79	0.1742	-0.6823	H	7.2009	-3.5341	-0.0951
C	1.296	-1.118	-0.59	O	3.0201	-3.8529	-1.9069
O	2.6872	0.1301	-1.9837	H	3.326	-4.7406	-2.1347
C	-0.37	0.5515	0.0227	C	4.585	0.263	0.2816
C	-0.9948	-0.4293	0.8152	C	5.8074	1.1602	0.4438
C	-0.4682	-1.7274	0.8879	C	7.157	0.5107	0.1204
C	0.6818	-2.0997	0.185	C	7.1484	-0.9246	0.6829
H	-1.8939	-0.1904	1.3835	O	3.4956	0.6409	0.6599
H	-0.9794	-2.4639	1.5146	O	8.3744	-1.6045	0.4814
H	1.0797	-3.1146	0.2416	H	9.0256	-1.2443	1.0954
O	1.6339	2.1196	-1.9061	H	6.9349	-0.8589	1.7734
O	-0.8018	1.8183	-0.1106	C	3.787	-1.8167	-0.9639
C	-1.952	2.2424	0.5876	C	2.5013	-1.2034	-1.5039
H	-1.822	2.1566	1.6816	H	2.2165	-1.8201	-2.3734
H	-2.8486	1.6701	0.2876	C	8.33	1.346	0.6377
H	-2.0995	3.2989	0.3268	H	7.2576	0.4119	-0.975
C	4.8026	-1.1125	-0.2766	H	5.7864	1.5265	1.4859
C	4.0183	-3.1705	-1.2752	H	5.6265	2.0483	-0.1863
C	5.2315	-3.7889	-0.9484	H	8.3092	1.4306	1.7389
C	6.2423	-3.0673	-0.3218	H	8.2942	2.3686	0.2296
C	6.0418	-1.7257	0.016	H	9.296	0.9083	0.3397

Table S9. ECD calculation of *ent*-donghaecyclinone C (**3d**).

total energy = -1222.729104885

kinetic energy = 1211.475176285

potential energy = -2434.204281170

Parameters of Level DFT

DFT settings (Functional B3LYP / Gridsize M3)

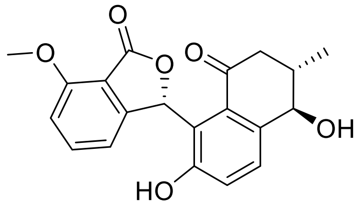

3d

Geometry optimization options (Energy 10-6 Hartree, Gradient norm │dE / dxyz│= 10-3 Hartree/Bohr)

Energy minimized coordinates of *ent*.-donghaecyclinone C at the basis set def-SVP for all atoms (Å).

Atom	X	Y	Z	Atom	X	Y	Z
C	-2.2706	1.0815	0.1424	H	-0.0931	-1.7961	-2.5788
C	-3.4726	0.5223	-0.2975	C	0.989	-2.3173	-0.8225
C	-1.0489	0.4713	-0.2207	C	2.3354	-1.9709	-0.862
C	-3.4677	-0.6307	-1.0764	C	2.48	-0.7001	-1.6137
C	-1.0232	-0.6704	-1.0539	O	3.4436	-0.0455	-1.8905
C	-2.2562	-1.2216	-1.4554	C	0.5374	-3.4579	-0.1618
C	-2.268	2.3514	0.9918	C	1.4995	-4.2424	0.4821
C	-1.1332	3.2776	0.5221	C	3.3072	-2.7605	-0.216
C	0.1995	2.5373	0.7044	C	2.8622	-3.911	0.4615
C	-1.1369	4.6285	1.239	H	-0.5202	-3.7279	-0.1454
H	-1.3029	3.4536	-0.5554	H	1.1892	-5.143	1.0197
C	0.1906	1.0427	0.4029	O	4.59	-2.3635	-0.2906
O	1.1351	0.3489	0.7173	H	3.5718	-4.556	0.9798
C	0.2305	-1.2987	-1.649	C	5.5933	-3.1125	0.3595
O	-2.225	-2.3443	-2.2293	H	6.5414	-2.5941	0.1639
H	-4.4106	-1.0702	-1.4186	H	5.6585	-4.1423	-0.0366
H	-4.4139	1.0092	-0.0396	H	5.4275	-3.1584	1.4511
H	-3.1215	-2.5752	-2.506	H	0.5648	2.6384	1.742
H	-0.9947	4.5002	2.3263	H	0.9893	2.9751	0.0691
H	-2.0896	5.1527	1.0793	H	-2.0567	2.0797	2.0477
H	-0.3191	5.2688	0.8707	O	-3.5106	3.0314	0.9176
O	1.2123	-0.3277	-2.0174	H	-4.0421	2.801	1.688
